# Development of Prolinol Containing Inhibitors of Hypoxanthine–Guanine–Xanthine Phosphoribosyltransferase: Rational Structure-Based Drug Design

**DOI:** 10.1021/acs.jmedchem.4c00021

**Published:** 2024-04-23

**Authors:** Dianne T. Keough, Magdalena Petrová, Gordon King, Michal Kratochvíl, Radek Pohl, Eva Doleželová, Alena Zíková, Luke W. Guddat, Dominik Rejman

**Affiliations:** † School of Chemistry and Molecular Biosciences, The University of Queensland, Brisbane, QLD 4072, Australia; ‡ Institute of Organic Chemistry and Biochemistry, Czech Academy of Sciences, Flemingovo nam. 2 , Praha 6 CZ-16610, Czech Republic; § The Centre for Microscopy and Microanalysis, The University of Queensland, Brisbane 4072, Australia; ∥ University of Chemical Technology Prague, Technická 5 , Prague 6 CZ-166 28, Czech Republic; ⊥ Institute of Parasitology, Biology Centre of the Czech Academy of Sciences, Branišovská 31, České Budějovice CZ-37005, Czech Republic

## Abstract

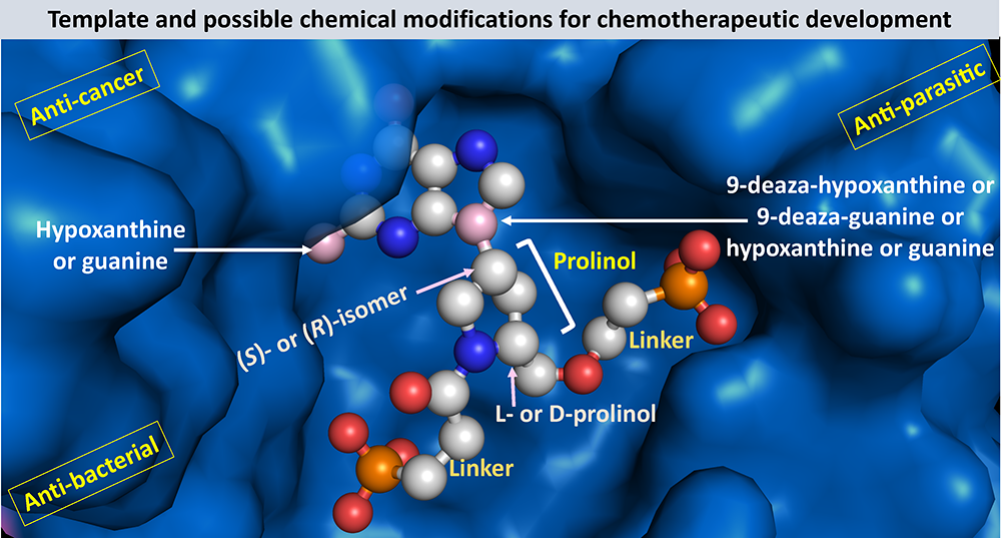

Inhibition of hypoxanthine–guanine–xanthine phosphoribosyltransferase activity decreases the pool of 6-oxo and 6-amino purine nucleoside monophosphates required for DNA and RNA synthesis, resulting in a reduction in cell growth. Therefore, inhibitors of this enzyme have potential to control infections, caused by *Plasmodium falciparum* and *Plasmodium vivax*, *Trypanosoma brucei*, *Mycobacterium tuberculosis*, and *Helicobacter pylori*. Five compounds synthesized here that contain a purine base covalently linked by a prolinol group to one or two phosphonate groups have *K*_i_ values ranging from 3 nM to >10 μM, depending on the structure of the inhibitor and the biological origin of the enzyme. X-ray crystal structures show that, on binding, these prolinol-containing inhibitors stimulated the movement of active site loops in the enzyme. Against *TBr* in cell culture, a prodrug exhibited an EC_50_ of 10 μM. Thus, these compounds are excellent candidates for further development as drug leads against infectious diseases as well as being potential anticancer agents.

## Introduction

**Figure 1:**
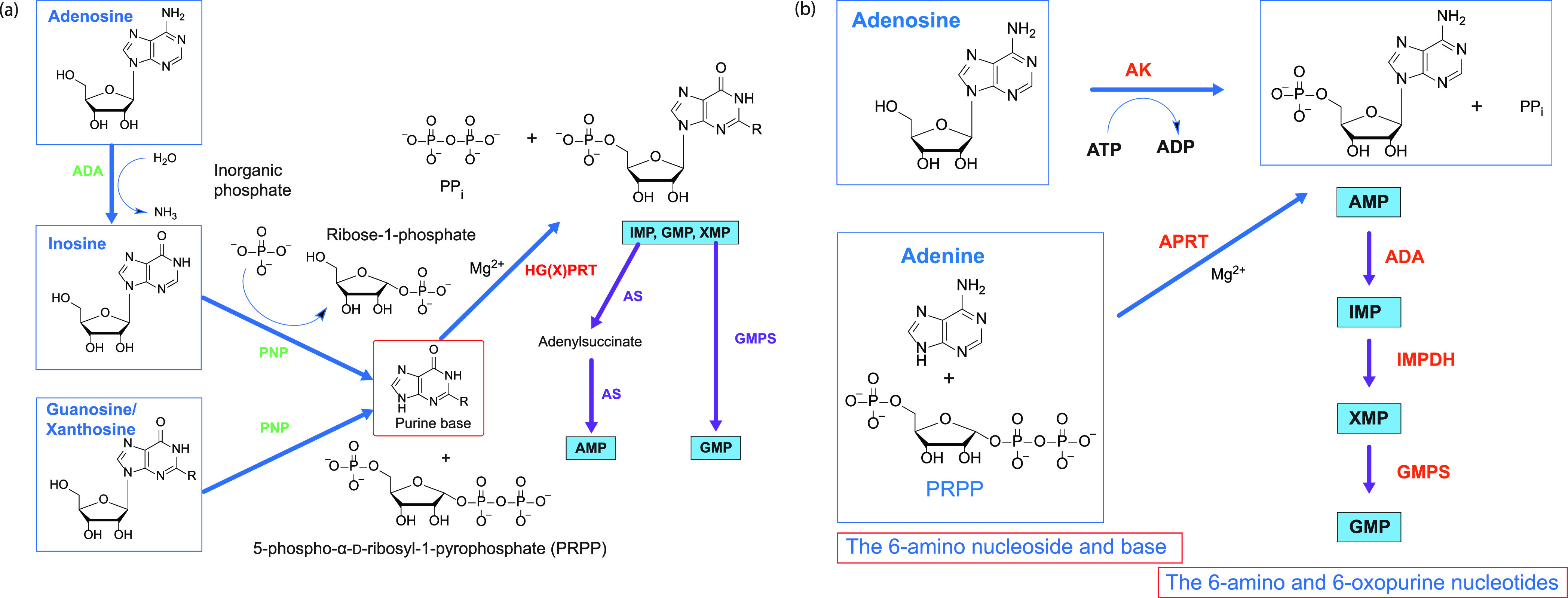
6-Oxo purine and the 6-amino purine nucleoside monophosphate synthetic pathways via salvage. (a) The first pathway for the synthesis of the 6-oxo purine and the 6-amino purine nucleoside monophosphates. (b) The second salvage pathway for the synthesis of the 6-amino and 6-oxopurine nucleoside monophosphates in *TBr* and *Hp.* ADA, AMP deaminase; IMPDH, IMP dehydrogenase; GMPS, GMP synthase; PNP, purine nucleoside phosphorylase. R = H (hypoxanthine) or −NH_2_ (guanine) or = O (xanthine). The molecules in the rectangles are imported from the host cell.

Infectious diseases are a scourge in the world today prompting the search for previously untried drug targets, which will result in new drug therapeutics. A recognized way to reduce the replication and survival of any cell is to halt DNA/RNA production. This approach proved successful in the development of the antivirals adefovir, cidofovir, and tenofovir.^1^ These drugs act by selective inhibition of the polymerases and by inserting foreign nucleotides into the growing DNA chain, causing premature chain termination.^1^ Nucleic acid production can also be restricted by inhibiting the synthesis of the precursor molecules, the purine nucleoside monophosphates. These essential metabolites are synthesized by two pathways: *de novo* and/or the salvage of purine bases or nucleosides. Humans possess the enzymatic machinery to use both routes while other organisms may have to rely completely on salvage. These include the parasites, *Plasmodium falciparum* (*Pf*) and *Plasmodium vivax* (*Pv*), which have circumvented the energetically demanding *de novo* pathway^2,3^ and have evolved to salvage the purine bases and nucleosides they need from the host cell (Figure 1). The key enzyme in the salvage pathway is hypoxanthine–guanine–(xanthine) phosphoribosyltransferase (HG(X)PRT)^4^ (Figure 1). The xanthine in brackets in the abbreviated name acknowledges the fact that only three of these enzymes *P. falciparum* (*Pf)*,^5^*Helicobacter pylori* (Hp),^6^ and *Escherichia coli* (Ec)^7^ are able to recognize xanthine as a substrate while the other four enzymes studied here cannot (human,^8^*Pv*,^9^*Trypanosoma brucei* (TBr),^10^ and *Mycobacterium tuberculosis* (*Mt*)).^11^ Thus, it is proposed that inhibition of HG(X)PRT activity will be effective in arresting the proliferation of these cells. It has previously been reported that *Plasmodium* cells can transport AMP but how relevant this is to the overall mononucleotide pool is yet to be ascertained.^12^ Purine nucleoside phosphorylase (PNP) is also a centrally important enzyme in the salvage pathway, using purine nucleosides and phosphate as substrates to produce the purine base and ribose-1-phosphate (Figure 1).^13^

In contrast, *TBr* and *Hp*, while also being unable to synthesize the purine ring,^14−18^ possess two additional enzymes, adenine phosphoribosyltransferase (APRT) and adenosine kinase (AK).^17−20^ Therefore, this parasite and this bacterium are also able to utilize adenine and adenosine as a source of their nucleotides (Figure 1).

However, in these two organisms, studies have shown that HG(X)PRT activity is crucial for growth and function. In *TBr*, this was demonstrated by double RNAi silencing^10^ while, in *Hp*, this was demonstrated by the need for exogenous purine bases/nucleosides.^18^*TBr* parasites cause Human and Animal African Trypanosomiases (HAT and AAT). Although HAT caused by *Trypanosoma brucei gambiense* and *Trypanosoma brucei rhodesiense* is progressing toward elimination of transmission and as public health problem, respectively,^21^ AAT continues to represent a significant economic burden due to lower meat and milk production, especially in affected areas of Sub-Saharan Africa. *Mtb* does indeed possess both *de novo* and salvage, but Griffin et al. used random transposon mutagenesis to identify that the *hpt* gene is essential for growth.^22^ In addition, human HGPRT is also being recognized more widely as a target for drug discovery against human cancers.^23−26^ Taken together, these studies demonstrate the crucial role that HG(X)PRT activity plays in almost all organisms.

**Figure 2:**
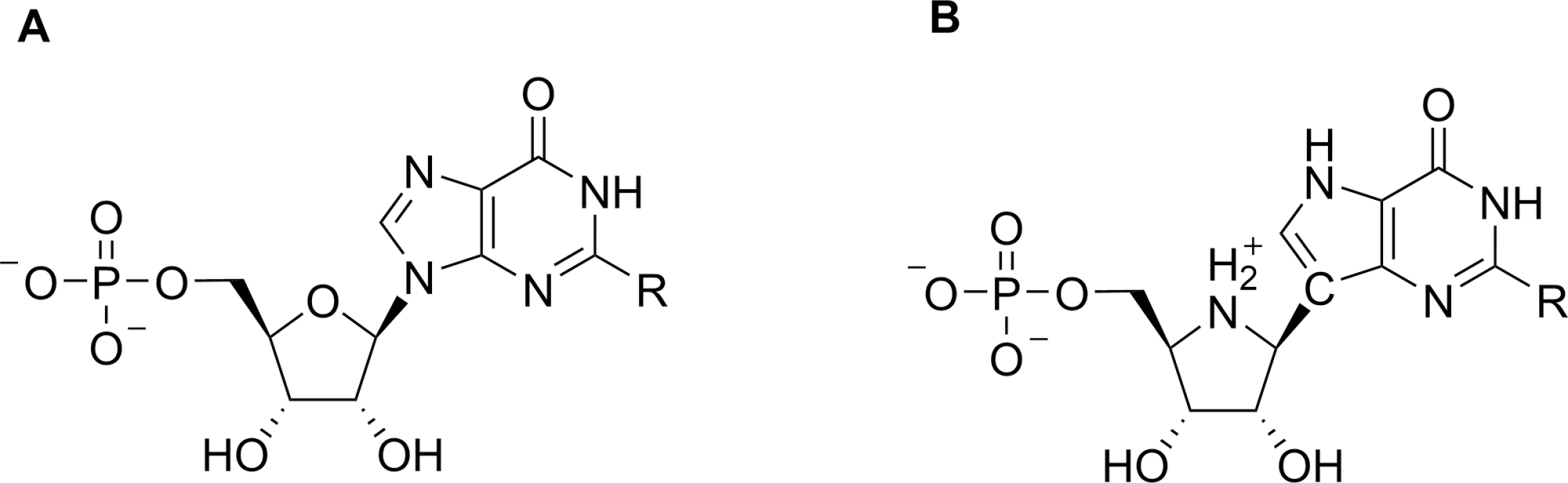
(A) The nucleoside monophosphates, IMP, GMP, and XMP. R is −NH_2_ (guanine), −H (hypoxanthine), or =O (xanthine). (B) The transition-state analogues, ImmHP or ImmGP.

Using target-based design, compounds have been synthesized and examined for their ability to inhibit HG(X)PRT from several sources, including human, *Pf*, *Pv*, *Escherichia coli*, *Mt*, *Hp* and *TBr.* Noting that *TBr* possesses three isoforms identified as HGPRT1, HGPRT2, and HGXPRT, HGPRT1 is localized in the cytoplasm whereas the other two enzymes are found in the glyocosome of *TBr.*(10) The scaffolds of these inhibitors are based on mimicking the substrates, products, and transition-state analogues of the enzymatic reaction. Figure 2A shows the structure of the nucleoside monophosphate product of the reaction catalyzed by HG(X)PRT together with that of the transition-state analogues of the catalytic reaction, (1*S*)-1-(9-deazahypoxanthin-9-yl)-1,4-dideoxy-1,4-imino-d-ribitol 5-phosphate (ImmHP) and (1*S*)-1-(9-deazaguanin-9-yl)-1,4-dideoxy-1,4-imino-d-ribitol 5-phosphate (ImmGP; Figure 2B).^27^ The transition-state analogues are not ideal as drug leads since the phosphate group is susceptible to chemical/enzymatic hydrolysis *in vivo*.^27^

**Figure 3:**
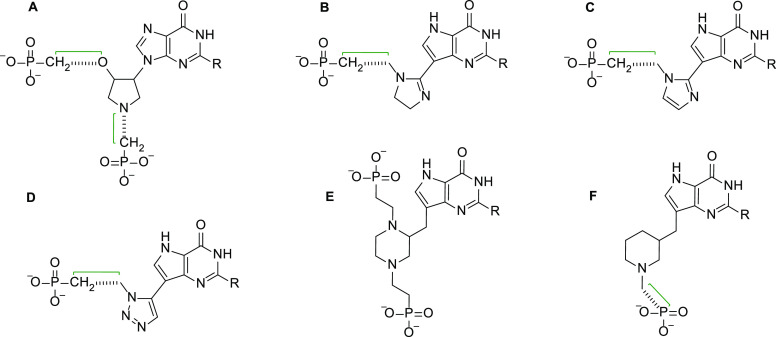
Phosphonate inhibitors of HG(X)PRT containing different heterocycle groups in the linker. The green bracket indicates where the linker can be further chemically modified to enhance affinity. (A) Pyrrolidines.^33,34^ (B) Imidazolines.^35^ (C) Imidazoles.^35^ (D) Triazoles.^35^ (E) Piperazines.^36^ (F) Piperidines.^36^

A major advance in inhibitor design was to replace the phosphate group with a stable phosphonate group.^28^ A second was to engineer into the design a different sequence of atoms into the linker connecting the purine base to the phosphonate group.^29−32^ One idea was to introduce different heterocycles into this linker representing, in part, the ribose ring of the mononucleotide product of the catalytic reaction (Figure 2A). The scaffolds for these inhibitor designs are given in Figure 3.

The *K*_*i*_ values for these compounds vary from 1.3 ± 0.5 and 2 ± 0.5 nM for [3*R*,4*R*]-4-hypoxanthin-9-yl-3-((*S*)-2-hydroxy-2-phosphonoethyl)oxy-1-*N*-(phosphonopropionyl)pyrrolidine with human HGPRT and *Pf*HGXPRT, respectively,^37^ to >10 μM for diisopropyl (3-(2-(4-oxo-4,5-dihydro-3*H*-pyrrolo[3,2-*d*]pyrimidin-7-yl)-1*H*-imidazol-1-yl)propyl)phosphonate with human HGPRT.^35^ The variability in the *K*_*i*_ values is attributed to three factors: (i) the organism from which the enzyme originates; (ii) the chemical structure of the heterocycle, which contributes to placing the functional groups in their optimal location; and (iii) the type of chemical groups attached to the heterocycle (Figure 3). The X-ray crystal structures of the best of these inhibitors with a HG(X)PRT from several organisms pointed the way for the design of the new inhibitors.

**Figure 4:**
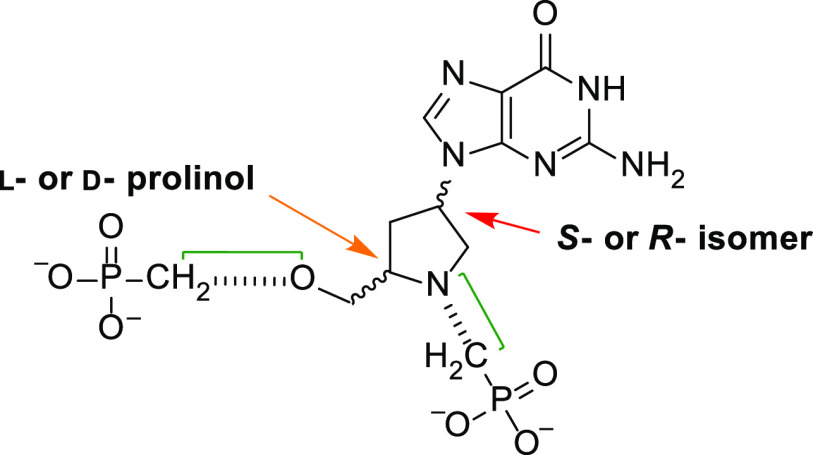
Scaffold for the design of the inhibitors containing a prolinol group in the linker. The arrows show the d- and l-enantiomers (orange arrow) and the isomeric attachment of the prolinol ring to the purine base (red arrow). The green brackets indicate areas where chemical modifications can be made. The base is also amenable to substitution with other purine bases.

These newly synthesized compounds contain a different heterocycle in the linker (Figure 4). This is a d- or l-prolinol group, which is attached to the purine ring as the (*S*) or (*R*) isomer. The chemical versatility of the prolinol group allows for the attachment of different groups to the nitrogen or carbon atom to enhance binding in the active site of HG(X)PRT from humans, parasitic protozoa, bacterial, or mycobacterial origins. The scaffold for this design is given in Figure 4.

Here, the *K*_*i*_ values for five such compounds have been measured against the H/G/X/PRTs from seven organisms. The / symbol signifies that these enzymes could have specificity for either one, two, or all three of the naturally occurring 6-oxopurine bases. The X-ray crystal structures of three of the prolinol inhibitors in complex with human HGPRT and *TBr*HGPRT1 have been obtained, providing structural insights into the reasons for the range of values of the inhibition constants not only between the compounds themselves but also between the enzymes from these organisms.

## Results and Discussion

### The Prolinols

**Figure 5:**
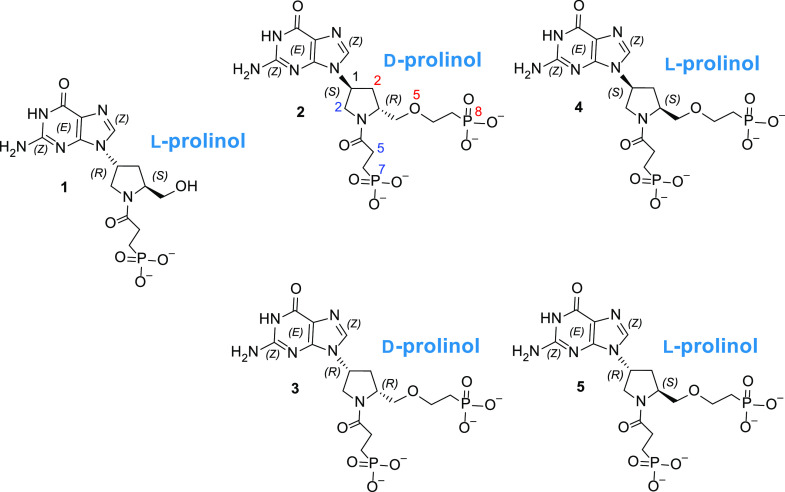
Chemical structure of the five prolinol compounds (**1**–**5**). The numerals show the number of atoms between the N^9^ of the purine ring and the phosphorus atom. There are seven atoms between the phosphorus atom at the termini of the linker, which is attached to the carbon atom of the five-membered ring (red numerals) and six between the phosphorus atom at the termini of the linker attached to the nitrogen atom of the five-membered ring (blue numerals).

The chemical structures of the five prolinols are given in Figure 5. These compounds all have guanine as the purine base and the chemical attachments to the carbon or nitrogen atom of the prolinol ring are identical. The differences between these molecules depend on whether the prolinol group exists as the l- or d-enantiomer and/or whether the prolinol is attached to the N^9^ atom of the purine ring as the (*S*) or (*R*) isomer (Figures 4 and 5).

### Synthesis

**Scheme 1:**
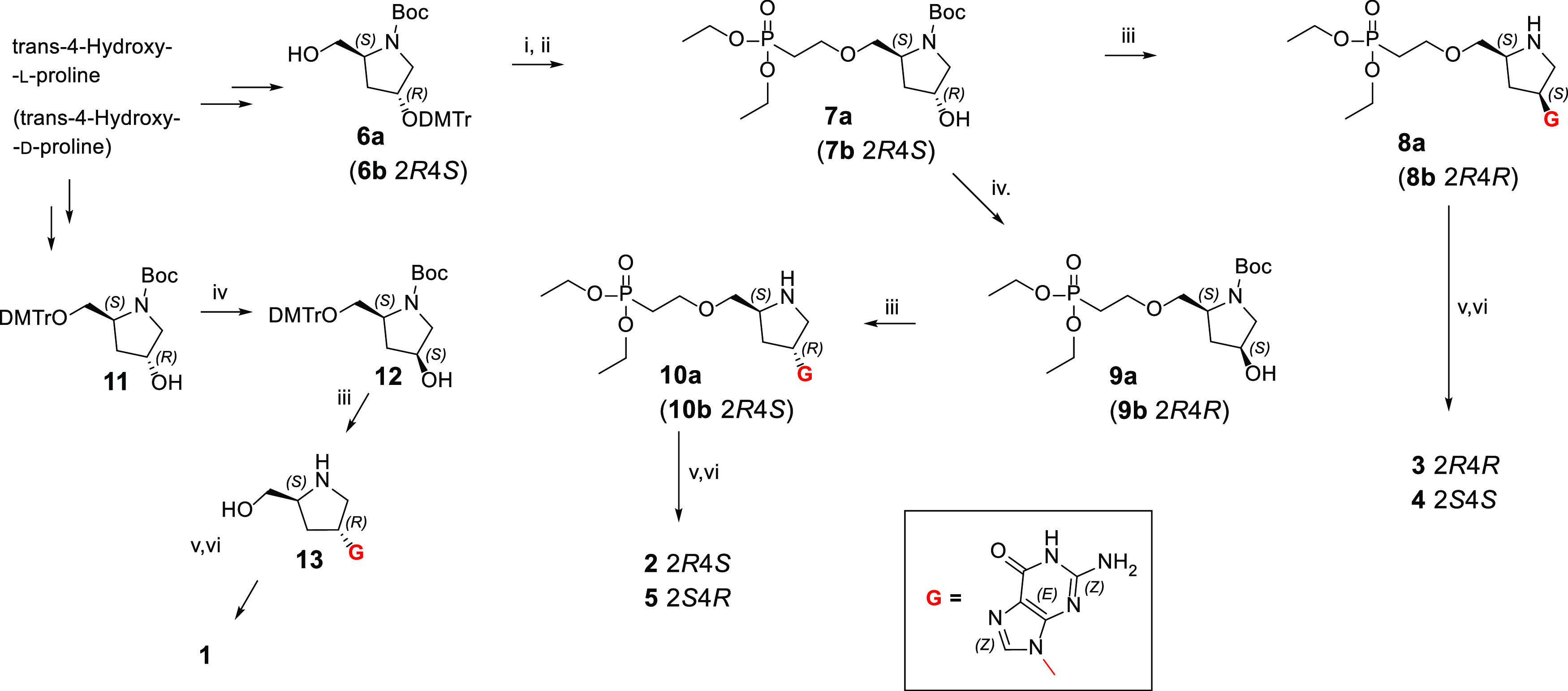
Synthesis of Prolinol Phosphonate and Bisphosphonates, **1**–**5** (see Figure 5)

The newly designed compounds were prepared, as outlined in Scheme 1. Michael addition of diethyl vinylphosphonate to protected pyrrolidine **6a** (or enantiomeric **6b**) afforded phosphonate **7a** (**7b**). In the next step, a guanine nucleobase was introduced by Mitsunobu reaction with 6-chloro-2-aminopurine and subsequent acidic hydrolysis yielded **8a** (**8b**). A second phosphonate function was added by reaction with diisopropyl 3-phosphonopropionic acid catalyzed by EDC. The phosphonate esters were removed by treatment with TMSBr resulting in the two bisphosphonates, **3** and **4**. Inversion of configuration of pyrrolidine carbon atom 4 of **7a** (**7b**) via Mitsunobu esterification with 4-nitrobenzoic acid followed by methanolic ammonia ammonolysis afforded phosphonate **9a** (**9b**). In the next step, the guanine nucleobase was introduced by Mitsunobu reaction with 6-chloro-2-aminopurine and subsequent acidic hydrolysis, yielding **10a** (**10b**). Reaction with diisopropyl 3-phosphonopropionic acid catalyzed by EDC and TMSBr promoted phosphonate ester groups removal to give the final compounds, **2** and **5**. Compound **1** was prepared from the protected hydroxyprolinol **11**. Mitsunobu esterification with 4-nitrobenzoic acid followed by methanolic ammonia ammonolysis afforded **12**, which then underwent Mitsunobu nucleosidation. The nucleoside, **13**, was reacted with diisopropyl 3-phosphonopropionic acid catalyzed by EDC, and subsequent TMSBr promoted phosphonate ester group removal to produce **1**.

### Inhibition Activity

Table 1 compares the Michaelis–Menten constants for the two substrates (PRPP and guanine), the *K*_*i*_ values for GMP (a nucleoside monophosphate product of the catalytic reaction) with the *K*_*i*_ values for the prolinol-containing inhibitors.

**Table 1:** Comparison of the Kinetic Constants of Seven H/G/X/PRTs for Their Naturally Occurring Substrates/Products with Those of the Five Prolinol Containing Inhibitors

**enzyme**	**PRPP***K*_m_**(μ**Μ) **(PRPP)**	**base (G)***K*_m_**(μ**Μ) **Guanine**	**GMP***K*_i_**(μΜ**) **GMP**	**1***K*_i_**(μ**Μ) l**-prolinol(***R***) isomer**	**2***K*_i_**(μ**Μ) d**-prolinol(***S***) isomer**	**3***K*_i_**(μ**Μ) d**-prolinol(***R***) isomer**	**4***K*_i_**(μ**Μ) l**-prolinol(***S***) isomer**	**5***K*_i_**(μ**Μ) l**-prolinol(***R***) isomer**
Mammalian								
human HGPRT	65 ± 5^38^	1.9 ± 0.4^38^	5.8 ± 0.2^39^	0.09 ± 0.2	0.14 ± 0.3	0.03 ± 0.01	0.009 ± 0.001	0.003 ± 0.005
Parasites								
*Pf* HGXPRT	69 ± 4^38^	0.83 ± 0.5^38^	10 ± 1^40^	0.5 ± 0.2	>10	2 ± 0.5	0.06 ± 0.03	0.01 ± 0.005
*Pv* HGPRT	73 ± 3^41^	1.9 ± 0.4^41^	26.1 ± 2^41^	2.7 ± 1	>10	1 ± 0.5	0.02 ± 0.005	0.06 ± 0.01
*TBr* HGPRT1	31 ± 3^42^	2.3 ± 0.4^42^	30.5 ± 0.2^42^	0.03 ± 0.01	>10	0.2 ± 0.06	0.07 ± 0.02	0.003 ± 0.0006
Mycobacteria and Bacteria								
*Mtb* HGPRT	465 ± 15^43^	4.4 ± 0.4^33^	20 ± 3^43^	6 ± 2	>10	2 ± 1	1.7 ± 0.2	0.3 ± 0.1
*Hp* XGHPRT	27 ± 4^34^	3.1 ± 0.2^34^	23 ± 4^34^	1 ± 0.2	>10	0.1 ± 0.04	7 ± 2	0.1 ± 0.03
*Ec* XGPRT	64 ± 3^44^	4.3 ± 0.3^45^	4.5 ± 0.3^44^	4 ± 2	>10	1 ± 0.4	9 ± 2	4 ± 2

The *K*_m_ values for the two substrates, PRPP and guanine, and for one of the products of the reaction, GMP, are similar for the human, protozoan parasite, bacterial, and mycobacterial enzymes (Table 1). The exception is the *K*_m_ for PRPP for *Mtb*HGPRT, which is significantly higher than those for the other six enzymes cf. 465 μM with 27–73 μM. The *K*_m_ for guanine for all these enzymes ranges between 0.8 and 4.4 μM (5-fold) and, for GMP, the *K*_i_ values lie between 4.5 to 30.5 μM (6.8-fold) (Table 1). The spread of *K*_i_ values for the prolinols presented here is much wider than the *K*_m_ values for the substrates and *K*_i_ values for the products (Table 1). For **1**, the range of inhibition constants between the seven enzymes is 200-fold, for **3**, the range is 33-fold, for **4**, it is 900-fold, and for **5**, it is around 1300-fold. One hypothesis to explain this data is that there are differences in the binding modes to the human, protozoan parasitic, bacterial, and mycobacterial enzymes which result in nonidentical interactions with active site side chain and main chain atoms. **2** is a very weak inhibitor of the enzymes from the pathogens having *K*_*i*_ values greater than 10 μM. It is a reasonable inhibitor of human HGPRT, though, demonstrating active site differences between the human enzyme and the enzymes from both the parasites and bacteria. These differences should be able to be exploited for selective drug design. Surprisingly, all the new compounds are comparatively weak inhibitors of *Ec*XGPRT, with the best, **3**, having a *K*_i_ of 1 μM. For human HGPRT, *Pf* HGXPRT and *Pv*HGPRT, the inhibitors with the lowest *K*_i_ values are **4** and **3** while, for *TBr*HGPRT1, **1**, **4**, and **5** have low *K*_i_ values (3–30 nM). **5** has the lowest *K*_i_ value for *Mtb*HGPRT, and **3** and **5** are the best inhibitors of *Hp*XGHPRT. In general, therefore, **5** is the most effective broad-spectrum inhibitor though the degree of inhibition varies with *K*_i_ values between 3 and 300 nM for six of the enzymes and a *K*_i_ for *Ec*XGPRT of 4 μM (Table 1).

Overall, the compounds with an l-prolinol in the linker are better inhibitors of these seven enzymes than those containing a d-prolinol group cf. **2** with **4** [(*S*) isomers] and **3** with **5** [(*R*) isomers]. There are only two exceptions to the preference for the l-prolinols. This is for the two bacterial enzymes, *Hp*XGHPRT and *Ec*XGPRT (Table 1). *Hp*XGHPRT has the same *K*_i_ values for **3** and **5** (0.1 μM) while, for *Ec*XGPRT, the *K*_i_ value is lower for **3** compared with **5** (1 μM *cf* 4 μM). **2** and **3** are d-prolinols and differ only in the isomeric attachment of prolinol group to the purine ring, (*S*) and (*R*), respectively. **4** and **5** are l-prolinols and also differ from each other only in the isomer that is attached.

The attachment of the prolinol group to the purine ring for both the l- and d-prolinols resulted in lower *K*_i_ values when this is as the (*R*)-isomer compared with the (*S*)-isomers (cf. **2** with **3** and **4** with **5**). Indeed, for the d-prolinols as the (*S*)-isomer (**2**), the compounds bind very weakly to the protozoan parasite and bacterial enzymes (*K*_i_ > 10 μM), although this compound is a reasonable inhibitor of human HGPRT (*K*_i_ = 0.14 μM). The *K*_i_ ratio between these two isomers for the d-prolinols varies between 7 for the enzyme from *Mt* to 10 for the human and *Pf* enzymes to 17 for *Pv*HGPRT and 66 for *TBr*HGPRT1. Thus, for the d-prolinols, the biggest effect of the changes in the isomers is for *TBr*HGPRT1.

**1** is a l-prolinol with the five-membered ring attached to the purine base as the (*R*)-isomer. The effect of a second phosphonate group added to the five-membered ring can be determined by comparing inhibition constants for **1** and **5** (Figure 5 and Table 1). The absence of this second phosphonate group resulted in an increase in the *K*_i_ values. The scale of this increase depends on the species of the H/G/XPRT. For example, the greatest increase occurs between the human and the two *Plasmodium* enzymes (30–45-fold; *cf***1** with **5**). The increase in *K*_i_ values for the enzymes from *TBr*, *Mtb*, and *Hp* was less significant being of the order of 10–20-fold, while the *K*_i_ values for *Ec*XGPRT were the same (4 μM). The crystal structures of human HGPRT in complex with either **1** or **5** were determined to assess the reason for this effect. In general, the best inhibitors of these prolinol containing compounds are the l-prolinols with this five-membered ring attached to the N^9^ atom of the purine ring as the (*R*)-isomer (i.e., **5**).

The effect of one or two phosphonate groups was deducted by comparing the structures of human HGPRT in complex with **1** or **5**. Crystal structures of **5** in complex with human HGPRT or *TBr*HGPRT1 investigated any possible differences in the binding modes between the human enzyme and the enzyme from one of the parasites. The effect of the isomeric attachment at the N^9^ atom of the purine ring was examined by obtaining the crystal structures of two complexes, *TBr*HGPRT·**4** and *TBr*HGPRT1·**5**.

### The Crystal Structures of Human HGPRT in Complex with Compounds **1** or **5** and TBrHGPRT1 in Complex with Compounds **4** and **5**

The X-ray data collection and refinement statistics of the four complexes are presented in Table 2. The two human HGPRT structures refine as a tetramer in the asymmetric unit. The *TBr*HGPRT1·**4** complex refines as a dimer, while the *TBr*HGPRT1·**5** complex refines as two dimers.

**Table 2:** X-ray Collection and Refinement Statistics for the Four Enzyme Inhibitor Complexes

	humanHGPRT·**1**	*TBr*HGPRT1·**4**	humanHGPRT·**5**	***TBr***HGPRT1·5
**Crystal parameters**				
wavelength	0.95373	0.95373	0.95373	0.95373
space group	*P* 2_1_ 2_1_ 2_1_	*P* 4_2_ 2_1_ 2	*P* 2_1_	*P* 2_1_
unit cell length, *a*, *b*, *c*(Å)	74.46, 93.42, 129.32	93.03, 93.03, 105.70	55.64, 129.59, 64.33	56.86, 88.28, 94.31
unit cell angle, α, β, γ (°)	90, 90, 90	90, 90, 90	90, 103.80, 90	90, 106.05, 90
**Diffraction data**				
temperature (K)	100	100	100	100
resolution range^a^ (Å)	75.73–2.50 (2.59–2.50)	41.2–2.20 (2.25–2.20)	43.98–2.27 (2.41–2.27)	45.97–2.46 (2.55–2.46)
total reflections	73,412 (6943)	84,604 (5885)	129,885 (7590)	93,048 (7589)
unique reflections	31,919 (3156)	24,294 (1690)	43295 (2811)	37,219 (3162)
multiplicity	2.3 (2.2)	3.5 (2.5)	3.0 (2.7)	2.5 (2.4)
completeness (%)	99.90 (99.97)	99.96 (100)	98.97 (96.3)	97.22 (95.02)
mean I/σ(I)	7.32 (2.16)	19.31 (3.75)	9.41 (1.52)	6.57 (3.34)
Wilson *B*-factor (Å^2^)	37.40	27.77	28.01	27.49
R-pim	0.0448 (0.396)	0.019 (0.111)	0.040 (0.437)	0.091 (0.254)
CC1/2	0.997 (0.777)	0.998 (0.97)	0.997 (0.525)	0.989 (0.851)
**Refinement**				
reflections used in refinement	31887 (3146)	24173 (1670)	42442 (2791)	31799 (2550)
*R*_work_	0.200 (0.244)	0.163 (0.190)	0.358 (0.387)	0.229 (0.313)
*R*_free_	0.260 (0.339)	0.217 (0.253)	0.223 (0.289)	0.287 (0.367)
RMS (bonds Å)	0.006	0.012	0.004	0.006
RMS (°)	1.05	1.17	1.04	0.87
Ramachandran favored (%)	94.61	94.44	97.41	93.18
Ramachandran outliers (%)	0.66	0.29	0.12	0.29
clashscore^b^	11.3	5.30	6.56	8.92
**Components of the asymmetric unit**				
subunits	4	2	4	4
inhibitors	4	2	4	4
magnesium ions	3	2	8	0
water molecules	111	280	170	375

a The values in parentheses are for the outer resolution shell.

b A clash is defined as an atomic overlap of 0.4 A (Angstroms). The clash score is (1000x number of overlaps)/number of atoms in the structure.

**Figure 6:**
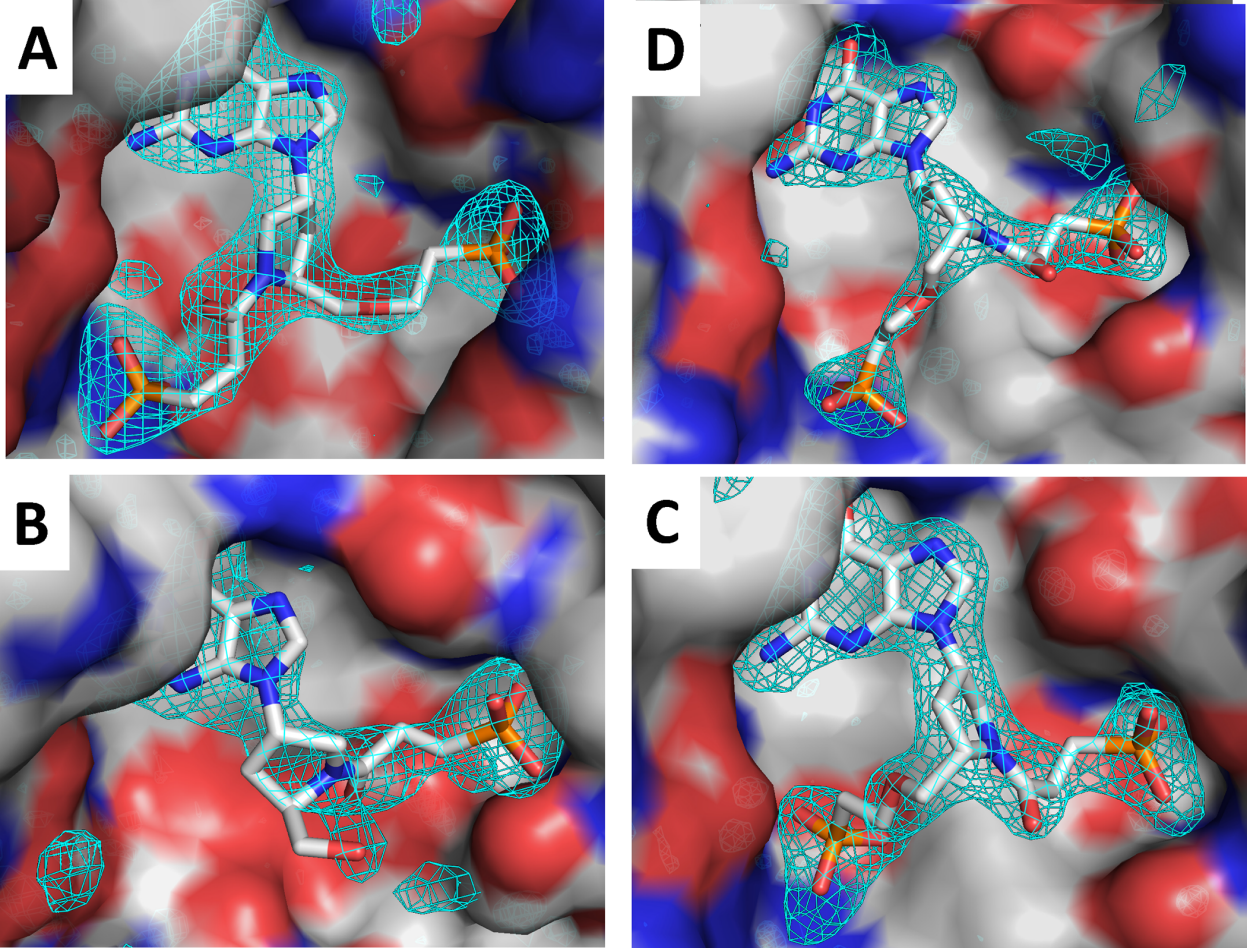
Connolly surface of human HGPRT and *TBr*HGPRT1 active sites with the electrostatic surface and *F*_o_–*F*_c_ omit electron density maps for **1**, **4**, or **5** overlaid. The maps are contoured at the 3.0 sigma level. (A) Human HGPRT·**5** complex. (B) Human HGPRT·**1** complex. (C) *TBr*HGPRT1·**4** complex. (D) *TBr*HGPRT1·**5** complex.

Figure 6 shows the electron density maps of the three inhibitors bound in the active site of human HGPRT or *TBr*HGPRT1. These structures clearly show the binding modes of the ligands to human HGPRT and *TBr*HGPRT1. They emphasize the movements that occur as the ligands adjust to obtain an optimal fit in active site of the HGPRTs from the two different species.

### Comparison of the Binding Mode of **1** and **5** in the Active Site of Human HGPRT

**Figure 7:**
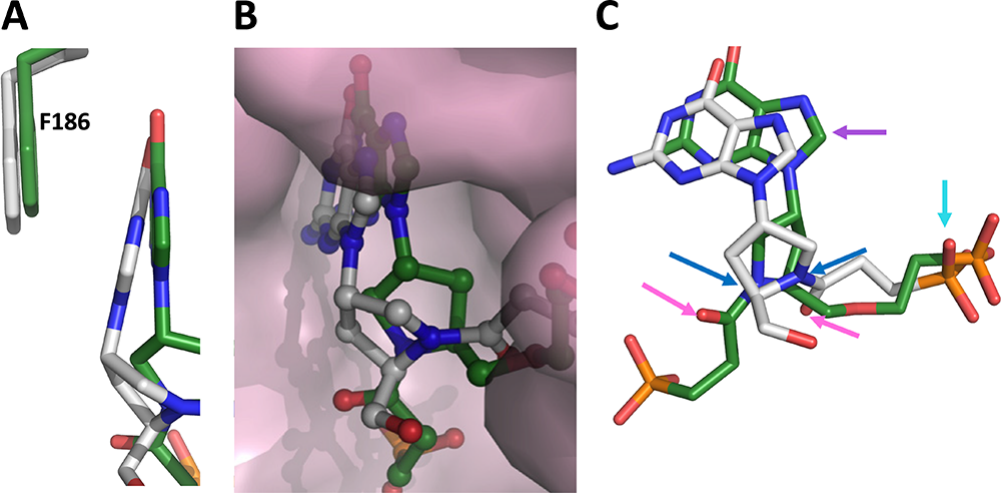
Comparison of the binding modes of **1** (silver) and **5** (green) in the active site of human HGPRT. (A) A side view of the binding of the purine ring of both compounds with F186. (B) Connolly surface of human HGPRT with **1** and **5** in their positions in the active site cavity. (**C**) The relative positions of **1** and **5** in the active site. The arrows highlight the differences in mode of binding of the functional groups: purple, purine base; cyan, phosphonate group; pink–carbonyl group; dark blue–nitrogen atom in the prolinol ring.

Figure 7 shows how compounds with one (**1**) or two (**5**) phosphonate groups bind in the active site of human HGPRT. Both contain a l-prolinol group in the linker, and this group is attached to the N^9^ atom of the purine ring as the *R-*isomer. Despite these commonalities, there are several differences in the binding modes of these two ligands (Figures 6A,B and 7). These include the purine base, the phosphonate group, and the carbonyl group (Figure 7B). In the human HGPRT·**5** complex, the purine ring forms a well-aligned π-stacking arrangement with the aromatic ring of F186 (Figure 7A). On the other hand, when **1** binds, the purine ring is tilted forward, away from the back of the active site and toward surrounding solvent (Figure 7A,B). Thus, the purine base is not anchored as effectively for **1** compared to **5**. This orientation of the purine ring is attributed to the contribution the second phosphonate group makes in placing the base in position.

**Figure 8:**
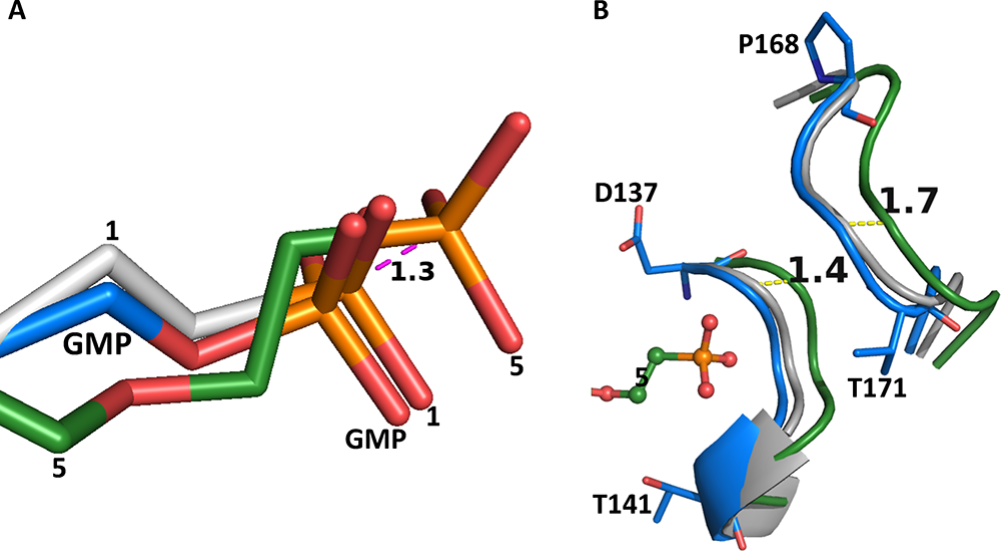
5′-Phosphate binding site in human HGPRT when three different ligands bind. (A) The position of the phosphate/phosphonate group in the human HGPRT, GMP, **1**, and **5** complexes. GMP is blue, **1** is silver, and **5** is green. (B) The position of the two flexible loops surrounding this binding site (residues 137–141 and residues 168–171). GMP is blue, **1** is silver, and **5** is green.

Another difference between these complexes is the positioning of phosphonate group in the 5′-phosphate binding pocket. For **5**, there are seven atoms in the linker between the phosphorus atom and N^9^ of the purine base but only six for **1** (Figure 5). In GMP, there are five atoms between N^9^ and the phosphorus atom. When **5** binds, it is the longer linker attached to the carbon atom of the prolinol group that positions the phosphonate group in this pocket while, in **1**, this site is occupied by the phosphonate group attached to the shorter linker, which comes off the prolinol nitrogen atom. On face value, it appears surprising that the human enzyme has been able to place the phosphonate group attached to the longer linker in this pocket as it was thought that there would be insufficient room in this cavity to accommodate it. However, the active sites of HG(X)PRTs, including human HGPRT, are known to be flexible.^46,47^ Since the electron density map (Figure 6A) clearly positions this phosphonate group in the 5′-phosphate binding pocket, this must mean either that the shape of the linker has to change to fit or there has to be a change in the structure of the enzyme itself. Figure 8 shows that it is the latter that occurs. The phosphonate group of **5** does push further into this pocket than the phosphonate group of **1** or **GMP** (Figure 8A), forcing the flexible loop (D137-T141) to move away (Figure 8B). The distance between the phosphorus atoms in **1** and **5** is 1.3 Å (Figure 8A), which is approximately the same distance that the loop moves. The movement of this loop triggers a further structural change, and this is between residues P168–V171 (Figure 8B). This loop also moves outward by approximately 1.7 Å. Figure 8B also demonstrates that residues 137–141 are adaptable, depending on the number of atoms connecting the phosphorus atom to the purine ring in the ligand. This movement in the structural complex is in the order GMP < **1** < **5** (Figure 7B). The structure of these two loops when **1** binds is a closer mimic of that when GMP binds.

It has previously been established based on crystal structures of human HGPRT with ImmGP^47^ and *Pf* HGXPRT in complex with ImmHP,^47^ and the acyclic immucillin phosphonate (AIP) inhibitor,^48^ that the side chain of the invariant aspartic acid, D137 (Figure 8), moves by at least 1 Å during catalysis, putting a carboxylate oxygen atom within 2.8 Å of the N^7^ atom of the purine base. In addition to these changes, a large flexible loop (R100 to T128, human HGPRT numbering)) also interacts with these transition-state analogues, resulting in sequestration of the active site from the solvent. However, when the prolinol inhibitors bind, neither does the large mobile loop cover the active site nor does a hydrogen bond form with the N^7^ atom of the purine base. In human HGPRT, the distance range is between 3.5 and 4.3 Å while, for *TBr*HGPRT1, it varies from 3.7 to 4.3 Å. For human HGPRT, the *K*_i_ values for the prolinol inhibitors are 90 nM (**1**), 9 nM (**4**), and 3 nM (**5**), and for the transition-state analogues identified above the *K*_i_ values range from 0.65 to 385 nM for *Pf*HGXPRT and human HGPRT.^48^ Thus, the prolinol-containing inhibitors are powerful inhibitors despite the fact that the enzyme structure has not folded as if it were progressing toward the transition state, as is the case for ImmG, ImmHP, and AIP.

The hydroxyl group in **1** (Figure 5) is not placed in the same position as either of the two hydroxyls of the ribose ring of GMP. The hydroxyl group, indeed, does not form any interactions in the active site and appears to make no contribution to the *K*_i_ value. It is possible, however, that it may help in shaping the 3D structure of the linker.

Compound **5** has two “wings” coming off the prolinol group, each of which has a phosphonate group at its termini. One of the factors that contributes to its low *K*_i_ value is the interactions that each of these phosphonyl oxygens make with side chain or main chain atoms in the active site.

### Comparison of the Binding Modes of **5** to Human HGPRT and *TBr*HGPRT1

**Figure 9:**
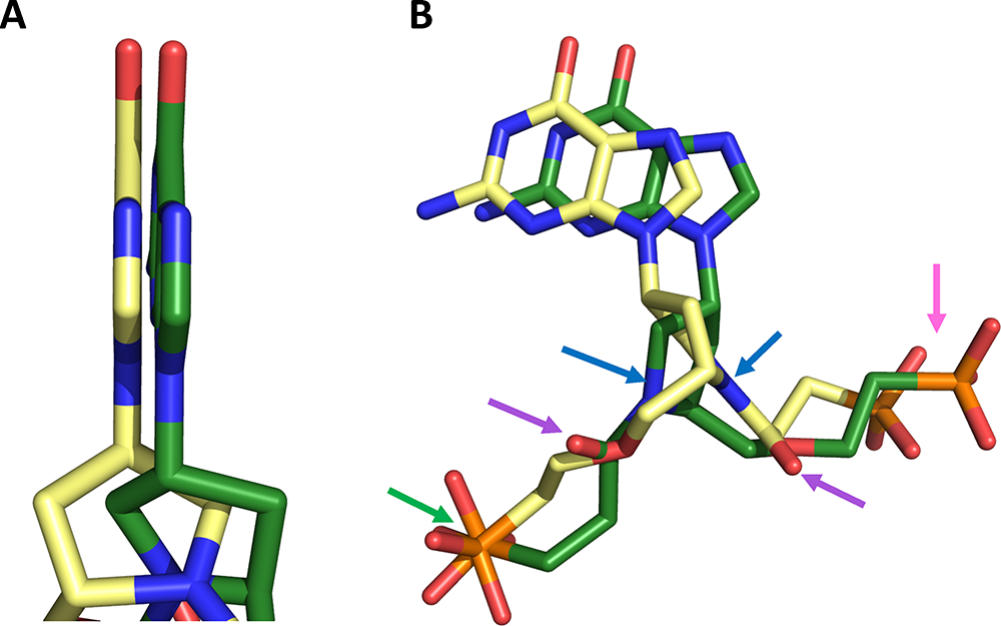
Comparison of the binding modes of **5** in the active site of human HGPRT (green) and *TBr*HGPRT1 (yellow). (A) The π-stacking arrangement of the purine ring. (B) The different conformations of the ligand. Human HGPRT (green) and *TBr*HGPRT1 (yellow). Arrows show the functional groups: pink, phosphonate group; dark blue, nitrogen in the prolinol ring; purple, carbonyl group; green, second phosphonate group.

**Figure 10:**
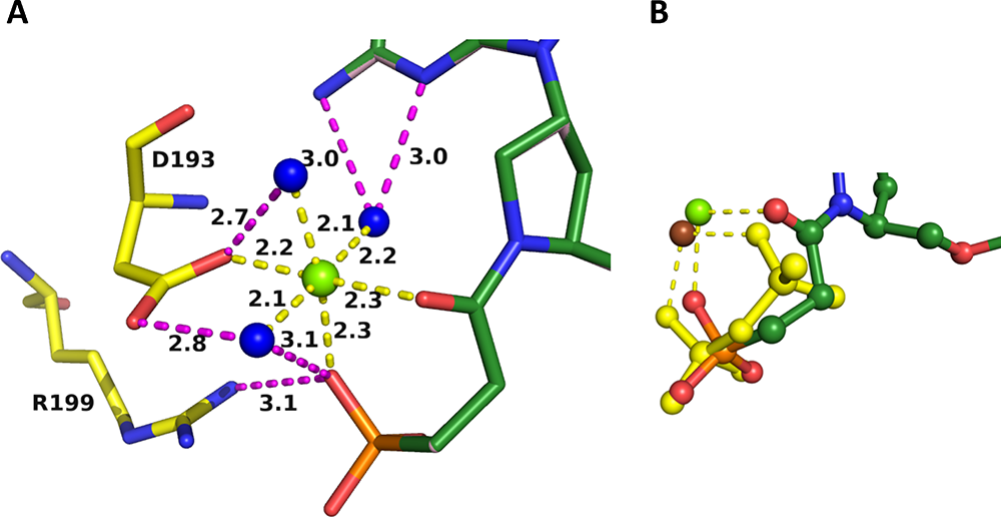
Binding site of the phosphonate group on the linker attached to the nitrogen in the prolinol ring in the structure of the human HGPRT·**5** complex. (A) Coordination of the phosphonyl oxygen and the carbonyl oxygen to a magnesium ion. Yellow dashed lines are the distances to a magnesium ion (green sphere). The blue spheres are water molecules, and the magenta dashes indicate hydrogen bonds. (B) Superimposition of the human HGPRT·**5** complex within the human. ImmGP.PP_i_ complex (PDB code: 1BZY). Yellow is the pyrophosphate in the transition-state complex.

**5** is a nanomolar inhibitor of both human HGPRT and *TBr*HGPRT1 (*K*_i_ = 3 nM). Thus, one prediction that could be made is that **5** would bind in a similar manner in each instance. However, this is not the case. In both these structures, the purine ring forms a parallel π-stacking arrangement with the aromatic side chain of the conserved aromatic amino acid, F158 in human HGPRT and F166 in *TBr*HGPRT1 (Figure 9A). However, the location of each of the two phosphonate groups in these enzymes is reversed (Figure 9B). In human HGPRT, it is the phosphonate group attached to the carbon atom of the prolinol ring that enters the 5′-phosphate binding pocket whereas, when **5** is bound to *TBr*HGPRT1, it is the group that is attached to the nitrogen atom (Figure 9B). A major driving force for binding of **5** could be the location of the carbonyl group in the shorter linker. In the human HGPRT·**5** complex, its position allows for coordination to a magnesium ion (Figure 10A). Perhaps, these interactions are not possible in the structure of *TBr*HGPRT1 and, hence, the change in orientation of the inhibitor. It is hypothesized that the loop surrounding this 5′-phosphate binding site is less flexible in *TBr* than the counterpart in the human enzyme (Figure 8B). Therefore, this loop is unable to move out to accommodate the longer linker and, as a result, the two phosphonate groups occupy alternate binding pockets in the human and *Tbr* enzymes. However, with this swap, the phosphorus atom in the pyrophosphate binding site is in the same location in both structures (Figure 10B).

**Figure 11:**
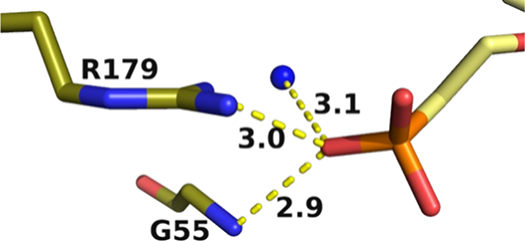
Interactions that the phosphonate group (attached to the nitrogen of the prolinol ring) of **5** makes when bound in the active site of *TBr*HGPRT1. The blue sphere is a water molecule.

The phosphonate groups that point down to the bottom of the active site of each enzyme superimpose on each other perfectly (Figure 9B; green arrow). Despite this, the phosphonyl oxygens form completely different interactions in the active site (cf. Figures 10 and 11).

In the human HGPRT·**5** complex, the phosphonyl and carbonyl oxygens are coordinated to a magnesium ion and this sets up a network of bonds which firmly anchors this inhibitor in place (Figure 10A). Superimposition of this complex onto that of the human enzyme in complex with the transition-state analogue, (1*S*)-1-(9-deazaguanyl-9-yl)-1,4-dideoxy-1,4-imino-d-ribitol 5-phosphate (ImmGP) together with pyrophosphate, shows that the phosphorus atom, the phosphonyl oxygens, and the carbonyl oxygen atoms of the inhibitor are located in a similar position as pyrophosphate (Figure 10B). These interactions may be one of the most important features of this ligand that contribute to the nanomolar *K*_i_ values for human HGPRT (3 nM; Table 1).

The carbonyl group in the *TBr*HGPRT1·**5** complex does not form any interactions with active site residues. However, **5** is still an excellent inhibitor of *TBr*HGPRT1with a *K*_i_ value of 3 nM. Therefore, it is not always the case that only identical binding modes produce the same inhibition constants. Many different factors can be the driving force for inhibitors with high affinity.

There are no magnesium ions in the *TBr*HGPRT1·**5** complex, and, as such, divalent metal ions do not play any role in anchoring this molecule in the active site. This is the opposite to the human HGPRT·**5** complex, which contains two magnesium ions (Table 2). In the case of the *TBr* enzyme, the phosphonate group is held in position by only three hydrogen bonds (Figure 11).

However, these bonds, together with the other interactions in the active site, are sufficient to result in a high affinity of the compound for the enzyme.

### Comparison of the Binding Modes of **4** and **5** in the Active Site of *TBr*HGPRT1

**Figure 12:**
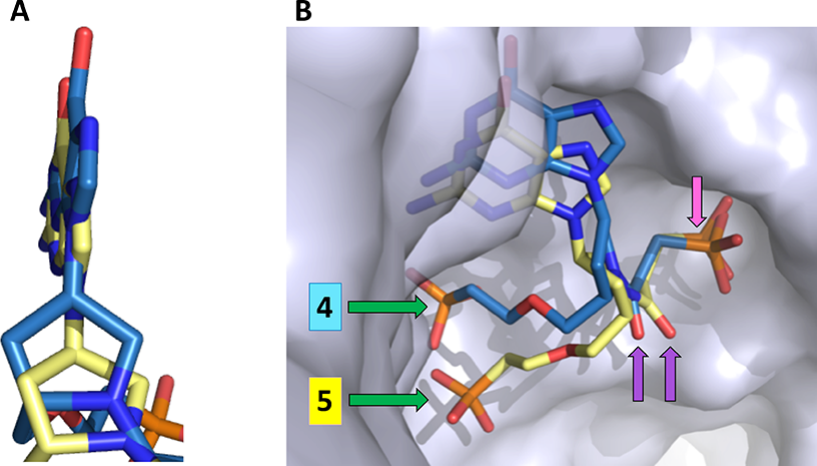
Comparison of the binding modes of **4** (blue) and **5** (yellow) in the active site of *TBr*HGPRT1. (A) The purine ring. (B) Connolly surface of the enzyme with the two ligands in the active site. The arrows show the location of three of the functional groups engineered into the scaffold.

Figure 12 compares the 3D structure of **4** with **5** when they are bound in the active site of *TBr*HGPRT1. There is only one difference in the chemical structure of these two ligands, and this is the isomeric attachment of the prolinol ring to the N^9^ atom of the purine ring (Figure 5). The (*R*) isomer does once again have the highest affinity (*cf. K*_i_ value of 3 nM with 70 nM) (Table 1).

**Figure 13:**
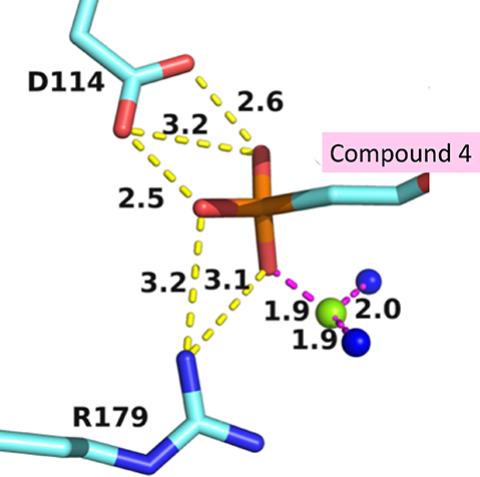
Interactions of the phosphonyl oxygens of the phosphonate group at the termini of the linker attached to the carbon atom of the prolinol ring in the*TBr*HGPRT1·**4** complex. The green sphere is a magnesium ion and blue spheres are water molecules.

Both purine rings form a π-stacking arrangement with the aromatic side chain of F166, and both phosphonate groups located in the 5′-phosphate binding pocket form identical interactions with active site side chain and main chain atoms (Figure 12A,B). As found for **5**, the carbonyl oxygen of **4** does not make any interactions in the active site. The difference in their binding, therefore, lies in the positioning of the phosphonate group attached to the nitrogen atom in the prolinol ring (Figure 12A). The interactions of the phosphonyl oxygens in the *TBr*HGPRT1·**5** complex are given in Figure 11 and those of **4** in Figure 13.

The large electron density found around the phosphonate group attached to the carbon atom in the prolinol ring when **4** binds has been assigned to a magnesium ion (Figure 13). The distances between the metal ion and water and the phosphonyl oxygen agree with the ligation chemistry. Thus, when this compound binds, a magnesium ion is found in an unpredicted locality and assists in binding this ligand.

### Other Interactions that Can Determine How Tightly the Ligands Bind to the Enzymes

Although the presence of magnesium ions is essential for catalysis to proceed in the H/G/X/PRTs, it does not play a direct role in catalysis. Rather, it assists in binding one of the two substrates (PRPP) and one of the two products of the reaction, i.e., PP_i_. It could be anticipated, therefore, that this ion could also play a role in helping the inhibitors to bind. One of the obvious ways this could be achieved is through coordination to the phosphonyl oxygens (Figures 10 and 13). Another possibility is through the OE1 and OD1 atoms of the two invariant acidic amino acid residues, glutamic and aspartic acid (133 and 134 in human HGPRT and 113 and 114 in *TBr*HGPRT1).

**Figure 14:**
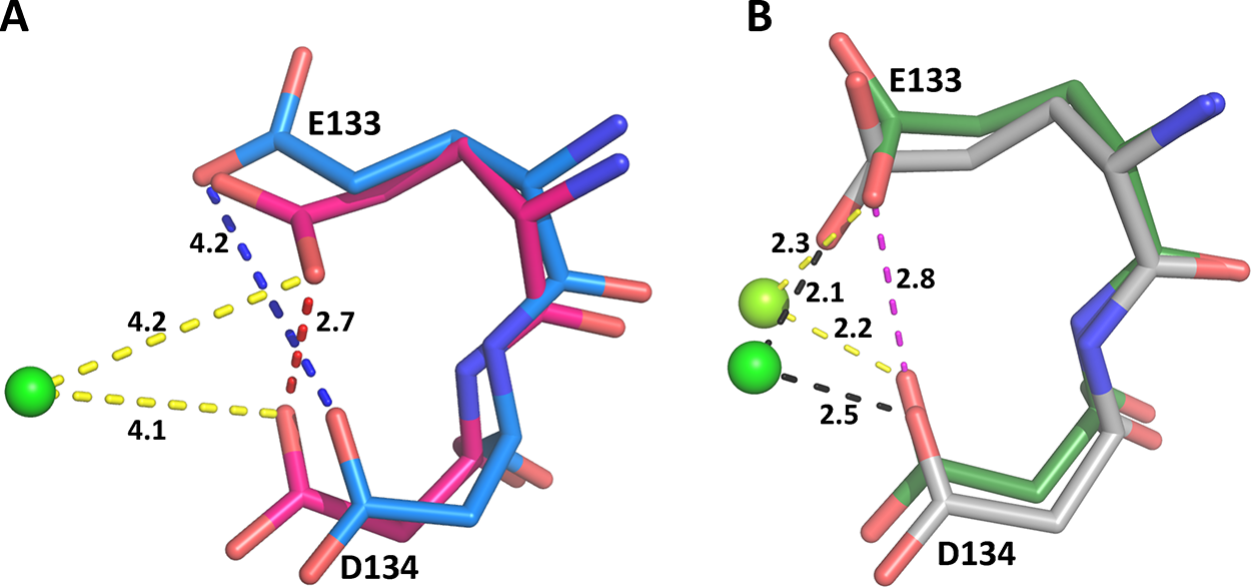
Movement of the side chains of E133 and D134 when four different ligands bind in the active site of human HGPRT. The green sphere is magnesium. (A) Blue is the human. GMP complex (PDB code: 1HMP). Red is when the transition-state analogue, (1*S*)-1-(9-deazaguanin-9-yl)-1,4-dideoxy-1,4-imino-d-ribitol 5-phosphate (ImmGP),^27^ is bound (PDB code: 1BZY). (B) Silver is when **1** and green is when **5** is bound in the active site.

**Figure 15:**
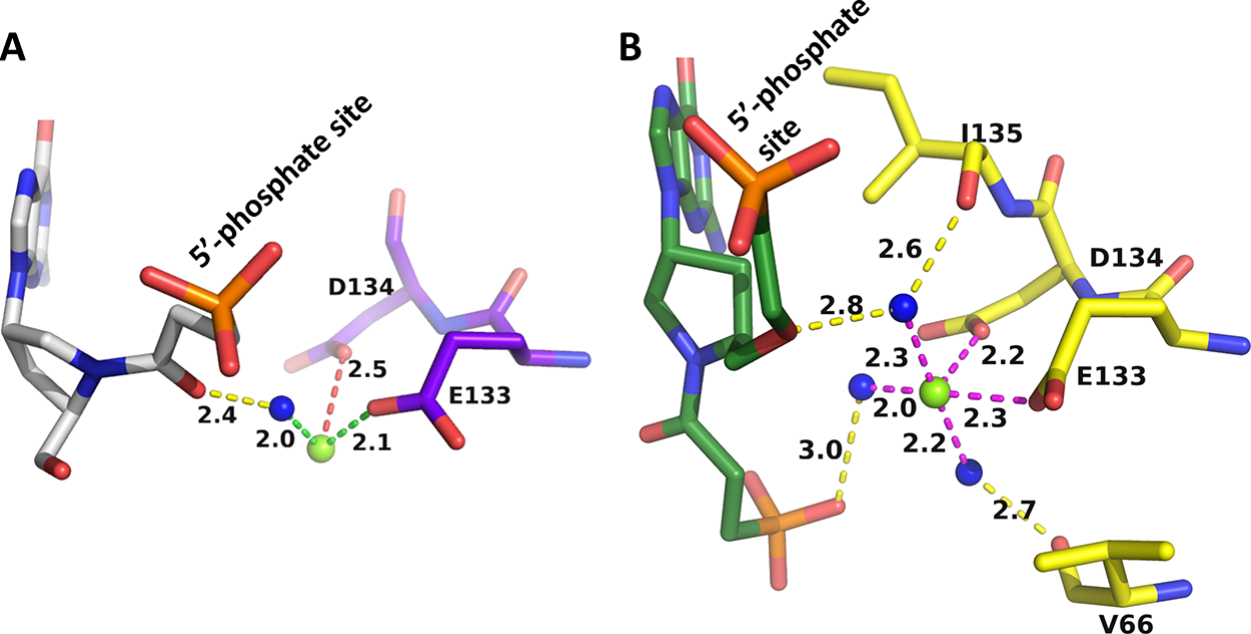
Assisted binding of **1** (A) and **5** (B) to human HGPRT via a divalent metal ion. Water molecules are shown as blue spheres and the divalent metal ion as green spheres. In (A), the coordination bonds are shown as green or red dashes and a hydrogen bond as yellow dashes. In (B), the coordination bond distances are shown as magenta dashes and hydrogen bonds as yellow dashes.

When ImmGP, PP_i_, and magnesium ion are bound in the active site of human HGPRT, the OE1 and OD1 atoms of the two acidic side chains are 2.7 Å apart (Figure 14A). In the transition-state complex, these oxygen atoms are not coordinated to a magnesium ion. Rather, they make hydrogen bonds to the hydroxyl groups of the ribose ring. To release the products of the reaction, the large mobile loop opens, the side chains move apart, and the distance between the OE1 and OD1 atoms becomes 4.2 Å (Figure 14A). This allows the release of the two products of the reaction, first pyrophosphate and the associated magnesium ion followed by the nucleotide, as has been proposed previously.^49−51^ When **1** and **5** bind to human HGPRT, the two side chains are 2.8 Å apart but, in this scenario, they coordinated directly to a magnesium ion (Figure 15B).

Thus, the two side chains of D133 and E134 are in the “closed” position, mimicking when ImmGP and pyrophosphate are bound. However, in the case of the inhibitors, a metal ion is in the vicinity (Figure 15B) and this allows a network of interactions to be established. In both cases, the tightness of binding is assisted via hydrogen bonds between water molecules and the inhibitor itself and via coordination to a divalent metal, which is then coordinated to the two invariant acidic amino acids, D134 and E133 (Figure 15A,B).

**Figure 16:**
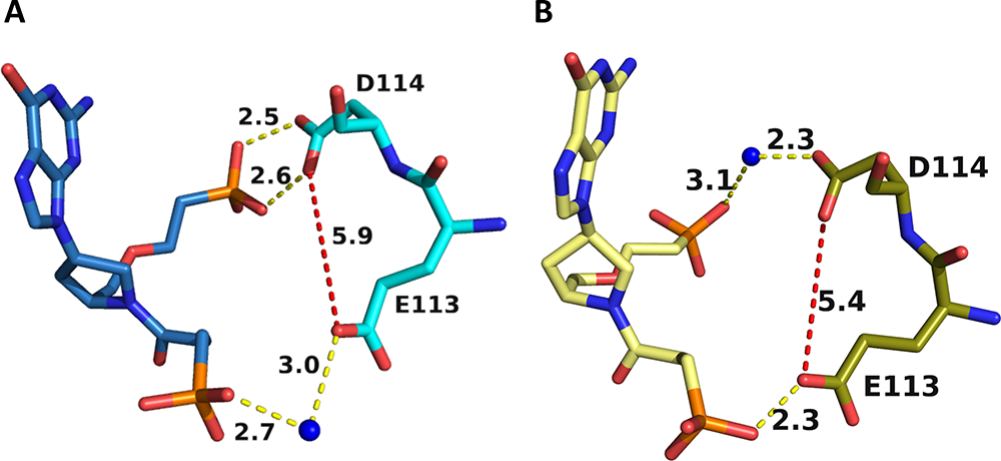
Position of E113 and D114 when **4** (blue; A) and **5** (yellow; B) bind in the active site of *TBr*HGPRT1.

In contrast, when **4** and **5** bind in the active site of *TBr*HGPRT1, the OD1 atoms of E113 and D114 are far apart (Figure 16) and there are no divalent metal ions in the vicinity. However, they can directly assist in binding the two ligands through several hydrogen bonds either directly to the phosphonyl oxygens or via a water molecule (Figure 16). Thus, although the precise interactions of the network differ, the OE1 and OD1 atoms of the invariant two acidic residues (ED) form bonds to the phosphonyl oxygens of the two phosphonate groups in both complexes and assist in anchoring these two inhibitors in the active site.

### Prodrugs of the Most Potent Inhibitors and Their Biological Activity

**Figure 17:**
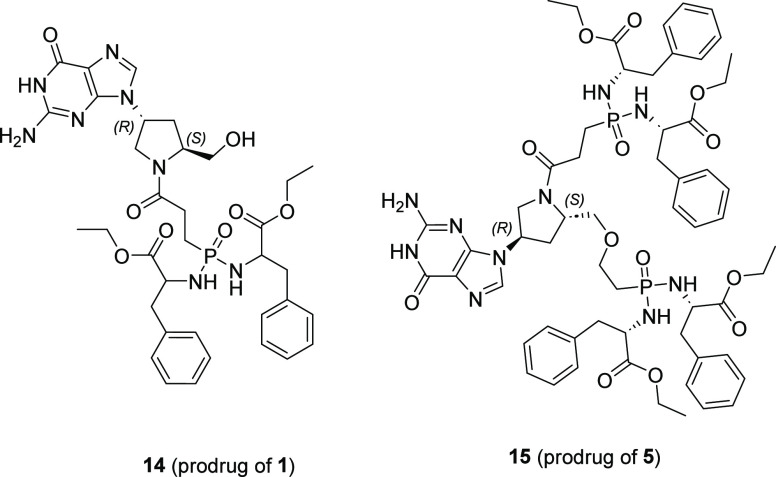
Structure of prodrugs **14** and **15**.

The prolinol inhibitors described here carry negative charges, making them unsuitable for biological testing against human pathogens. To begin to address this problem, phenylalanine amidate prodrugs **14** and **15** of inhibitors **1** and **5**, respectively (Figure 17), were prepared according to procedures described previously.^33^

**14** and **15** were tested for their antitrypanosomal activities against *Trypanosoma brucei brucei* and showed EC_50_ values of 9.98 ± 1.15 and 19.84 ± 0.27 μM, respectively. This result shows that in principle, if the prolinols can be delivered to the target pathogen, they can be effective in preventing the growth or killing a pathogen in culture.

The parent compounds, **1** and **5**, are both potent inhibitors of *TBr*HGPRT1 with *K*_i_ values of 30 and 3 nM, respectively, with the primary difference being that, while **1** possesses only one phosphonate group, **5** has two. Thus, prodrug **14** is significantly smaller (735.78 Da) than **15** (1195.26 Da). The lower EC_50_ value for prodrug **14** compared to **15** is thus likely due to **14** having a lower overall molecular weight, facilitating transport across the cell membrane. In addition, it has only one masking group to be removed, which may also contribute to its lower EC_50_ value. The studies suggest that both potent inhibition of the *TBr*HGPRT1 enzyme and effective prodrug design are needed. To date, prodrug design has focused on improving the bioavailability of drugs containing a phosphonate group against viral infections.^52^ More work is needed to improve potency for the bacterial and mycobacterial enzymes and for the delivery of compounds into more challenging environments such as *Plasmodium falciparum* within red blood cells and *Mycobacterium tuberculosis* within macrophages.

## Conclusions

The insertion of a prolinol group in the linker(s) connecting a purine base to one or two phosphonate groups allows for versatility in inhibitor design. The prolinol structure allows functional groups to be attached to this ring such that new modes of binding of H/G/X/PRT inhibitors can be explored. These newly designed compounds exhibit a wide range of *K*_i_ values for the human, parasitic, bacterial, and mycobacterial H/G/X/PRTs and, thus, can discriminate between enzymes in this class. For the human enzyme, the l-prolinol and (*R*)-isomer configuration is the preferred configuration (i.e., **5**). Key to this preference is the parallel π stacking of the purine base with the conserved aromatic amino acid in the enzyme (i.e., F186 in human HGPRT) (Figure 7) and the fact that the 5′-phosphate pocket can expand to bind the 5′-phosphonate of **5**. Such an expansion does not occur when GMP or **1** binds (Figure 8). The linker attached to the nitrogen of the prolinol also plays a critical role in affinity, with interactions to R199 and D193. In the latter, this is assisted by the presence of a Mg^2+^ and several water molecules (Figure 10A). The tail of the linker finds itself in a location that strongly mimics the location where pyrophosphate binds in the human ImmGP.PP_i_ complex (Figure 10B). The prolinol ring does not make many interactions with the enzyme but plays an essential role in that it positions the purine ring, the 5′-phosphate, and the linker in positions that allow strong interactions in all three sites. The *K*_i_ values for the parasitic enzymes follow a similar trend to the human enzyme, with **5** being the most potent followed by **4**. The *K*_i_ values of **5** for human and *Tbr*HGPRT1 are virtually identical at 3 nM. This might suggest identical modes of binding in the two enzymes, but this is not the case. The prolinol ring in *Tbr*HGPRT1 is rotated by ∼90° compared to its binding to the human counterpart (Figure 6). Nonetheless, the purine ring, the 5′-phosphate, and the linker all make highly favorable interactions with *Tbr*HGPRT1, although these are different to human HGPRT (Figures 6 and 9). The binding of all compounds in the series is substantially weaker to the bacterial and mycobacterial enzymes. Additional crystal structures will be needed to explain why this is the case. The X-ray crystal structures reveal that these inhibitors interact with the enzyme by a mechanism of “induced fit”. **5** is one of the most potent of these inhibitors, especially for the parasitic enzymes and, as such, is an excellent scaffold for further development as antiparasitic drug leads.

The *K*_i_ values of **1** for human HGPRT and *Tbr*HGPRT1 are 90 and 30 nM, respectively. For **5**, as mentioned above, both compounds have *K*_i_ values of 3 nM. Thus, neither compound has a strong selectivity for the parasite enzyme over the human counterpart. However, previous studies on phosphonate prodrugs that inhibit HGPRT have shown that they have low toxicity in human cell lines, with CC_50_ values usually >300 μM.^53,54^ This is proposed to be because humans possess both salvage and de novo pathways for the synthesis of nucleoside monophosphates. Thus, while it appears to be an advantage for an inhibitor to have selectivity for the parasite enzyme over the human enzyme in antiparasitic drug development, it may not be an absolute requirement.

In addition, nucleotide metabolism has long been recognized to provide multiple pathways for the development of new anticancer treatments.^55^ Recently, it has emerged that HGPRT is upregulated in malignant tumors and localized to the surface in some cancer cells,^56^ this likely due to the increased rate of DNA production compared to normally replicating human cells. Thus, human HGPRT inhibitors such as the prolinols synthesized here also have potential as anticancer therapeutic agents.

## Experimental Section

### Synthesis

#### General Conditions and Used Materials

Unless stated otherwise, all solvents were anhydrous. TLC was performed on silica gel-precoated aluminum plates TLC Silica gel 60 F_2__5__4_ (Supelco), and compounds were detected by UV light (254 nm), by heating (detection of the dimethoxytrityl group, orange color), by spraying with 1% solution of ninhydrine to visualize amines, and by spraying with 1% solution of 4-(4-nitrobenzyl)pyridine in ethanol followed by heating and treating with gaseous ammonia (blue color of mono- and diesters of phosphonic acid). Preparative column chromatography was carried out on silica gel (40–63 μm, Fluorochem), and elution was performed at the flow rate of 60–80 mL/min. The following solvent systems were used for TLC and preparative chromatography:toluene-ethyl acetate 1:1 (T), chloroform–ethanol 9:1 (C1), ethyl acetate–acetone–ethanol–water 6:1:1:0.5 (H3), and ethyl acetate–acetone–ethanol–water 4:1:1:1 (H1). The concentrations of solvents are stated in volume percent (%, *v*/*v*). The purity of intermediates was determined by LC-MS performed on Waters AutoPurification System with 2545 Quaternary Gradient Module and 3100 Single Quadrupole Mass Detector using a Luna C18 column (Phenomenex, 100 × 4.6 mm, 3 μm) at a flow rate of 1 mL/min. The usual conditions are as follows: mobile phase, A—50 mM NH_4_HCO_3_, B—50 mm NH_4_HCO_3_ in 50% *aq.* CH_3_CN, C—CH_3_CN, A → B/10 min, B → C/10 min, C/5 min. Preparative RP HPLC was performed on LC5000 Liquid Chromatograph (INGOS-PIKRON, CR) using the Luna C18 (2) column (250 × 21.2 mm, 5 μm) at a flow rate of 10 mL/min by a gradient of methanol in 0.1 M TEAB pH 7.5 (A = 0.1 M TEAB, B = 0.1 M TEAB in 50% aq. methanol, C = methanol) or without buffer (TEAB). All final compounds were lyophilized from water. The purity of the final compounds was greater than 95%. Purity of final compounds was determined via LC-MS analysis using ACQUITY UPLC coupled with Xevo G2-XS QTof (Waters) using a ZIC cHHILIC column (Sigma-Aldrich; 100 × 2.1 mm, 3 μm) with gradient elution: 70% B 0–2 min, 100% A in 9 min, hold until 10.5 min. A = 50% acetonitrile with 10 mM ammonium acetate; B = acetonitrile; flow = 0.3 mL/min. Detection was performed using full scan mode in ESI+ (or ESI−) mode of ionization. Mass spectra were recorded on LTQ Orbitrap XL (Thermo Fisher Scientific) using ESI ionization. Infrared (IR) spectra were recorded on a Thermo Scientific Nicolet 6700 spectrometer. Absorption maxima (ν_max_) are reported in wavenumbers (cm^–1^). Specific rotation values were determined with an Autopol IV (Rudolph Research Analytical, USA, 2001) polarimeter. Specific rotation values [α]_D_ were measured in H_2_O (concentration units: g/100 mL). NMR spectra were measured on a Bruker AVANCE III HD 400 MHz (^1^H at 400.1 MHz, ^13^C at 100.6 MHz, and ^31^P at 162.0 MHz), Bruker AVANCE III HD 400 MHz Prodigy (^1^H at 401.0 MHz, ^13^C at 100.8 MHz, and ^31^P at 162.0 MHz), Bruker AVANCE III HD 500 MHz (^1^H at 500.0 MHz, ^13^C at 125.7 MHz, and ^31^P at 202.4 MHz), and JEOL JNM-ECZR 500 MHz (^1^H at 500.2 MHz, ^13^C at 125.8 MHz, and ^31^P at 202.5 MHz) spectrometers. D_2_O (reference (dioxane) = ^1^H 3.75 ppm, ^13^C 69.3 ppm). Chemical shifts (in ppm, δ scale) were referenced to TMS as internal standard, and coupling constants (*J*) are given in Hz. All intermediates were determined by LC-MS.

##### General Method A: Reaction with Diethyl Vinylphosphonate and Subsequent Removal of the DMTr-Protecting Group

A mixture of hydroxy derivative (1 mmol), diethyl vinylphosphonate (1.5 mmol), and Cs_2_CO_3_ (1 mmol) in *t*-BuOH (2.5 mL/mmol) was stirred under an argon atmosphere at 50 °C for 2 days. The reaction mixture was concentrated in vacuo, and the product was obtained by column chromatography on silica gel using a linear gradient of EtOH in chloroform. This led to a partially pure product, but it was used for the next step (DMTr PG removal) without further purification and characterization. The product was dissolved in 2% TFA in CHCl_3_ (10 mL/mmol) and stirred for 10 min. Solid NaHCO_3_ (1 g/mmol) was added and the suspension stirred until the pH was neutral. The suspension was filtered and the filtrate dried in vacuo. The desired product was obtained by column chromatography on silica gel using a linear gradient of EtOH in chloroform.

##### General Method B: Inversion of Configuration

The mixture of the hydroxy derivative (1 mmol), triphenylphosphine (2.5 mmol), lutidine (1.5 mmol), and 4-nitrobenzoic acid (1.3 mmol) was coevaporated with THF (2 × 10 mL) and dissolved in the same solvent (10 mL/mmol). DIAD (2.5 mmol) was added under an argon atmosphere, and the reaction mixture was stirred at RT overnight. The reaction mixture was concentrated in vacuo, and 4-nitrobenzoic acid ester with inverted configuration was obtained by column chromatography on silica gel using a linear gradient of ethanol in chloroform. The product was dissolved in methanol and the solution saturated with gaseous ammonia at 0 °C. The mixture was left at RT overnight and concentrated *in vacuo*. The desired hydroxy derivative with inverted configuration was obtained by column chromatography on silica gel using a linear gradient of ethanol in chloroform.

##### General Method C: Mitsunobu Nucleosidation

DIAD (3.5 mmol) was added to the solution of diphenylpyridylphosphine or triphenylphosphine (3.5 mmol) in THF (5 mL/mmol), and the mixture was stirred at RT under an argon atmosphere for 30 min. The mixture was then added to the mixture of hydroxy derivative (1 mmol) and 2-amino-6-chloropurine (1.5 mmol) (coevaporated prior to the reaction with THF (2 × 10 mL) in THF (5 mL/mmol). The reaction mixture was stirred under an argon atmosphere at RT overnight. The reaction mixture was concentrated in vacuo, and the chloropurine product was obtained by column chromatography on silica gel using a linear gradient of ethanol in chloroform.

##### General Method D: Boc Group Removal and Nucleobase Hydrolysis

Protected chloropurine derivative (1 mmol) was stirred with EtOH (10 mL/mmol) and 3 M aq. HCl (10 mL/mmol) at 80 °C overnight. The reaction mixture was diluted with water:EtOH 1:1 (20 mL/mmol) and applied to a column of Dowex 50 in H^+^ form (20 mL/mmol). The resin was washed with 50% aq. ethanol (50 mL/mmol), and the crude product eluted with 3% ammonia in 50% aq. ethanol. After evaporation, the product was used in the crude form for the next reaction step or purified using HPLC on the reversed phase using a linear gradient of MeOH in water.

##### General Method E: Attachment of Phosphonopropionic Acid

EDC (3 mmol) was added to the mixture of starting material (1 mmol) and diisopropyl phosphonopropionic acid (1.2 mmol) in DMF (10 mL/mmol) and the reaction mixture stirred under argon at 90 °C for 4 h. The reaction mixture was concentrated in vacuo and the desired product obtained by column chromatography on silica gel using a linear gradient of the H1 system in ethyl acetate.

##### General Method F: Deesterification of Phosphonates

Di- or tetra-ester (1 mmol) was dissolved in MeCN (10 mL/mmol). TMSBr (5 or 7 mmol) was added and the reaction mixture stirred under an argon atmosphere at RT overnight. The solvent was removed in vacuo, the residue dissolved in 2 M aq. TEAB (5 mL/mmol) and EtOH (5 mL/mmol) and again concentrated. The target compound was obtained using preparative HPLC on a reversed phase with the linear gradient of MeOH in 0.1 M aq. TEAB. Fractions containing the desired product (according to LC-MS) were combined and evaporated. The residue was coevaporated with MeOH (3 × 10 mL/mmol) to remove all remaining TEAB. Finally, the product was converted to the sodium salt by chromatography on Dowex 50 in Na^+^ form. The final product was lyophilized from water to form a white solid.

##### General Method G Synthesis of Prodrugs

A mixture of phosphonate ester,^26^ (0.5 mmol), dry pyridine (8 mL), and BrSiMe_3_ (1 mL) was stirred overnight at room temperature under argon. After evaporation and codistillation with pyridine (2 × 5 mL) under an argon atmosphere, the residue was dissolved in dry pyridine (10 mL) and ethyl (l)-phenylalanine hydrochloride (1.75 g, 7.1 mmol) and triethylamine (3.1 mL) were added. The mixture was heated to 70 °C under an argon atmosphere, and then a solution of aldrithiol (2.31 g, 10.5 mmol) and triphenylphosphine (2.75 g, 10.5 mmol) in dry pyridine (8 mL) was added. The reaction mixture was heated at 70 °C for 3 days, the solvent was evaporated, and the residue was coevaporated with toluene (2 × 10 mL) and purified by column chromatography on silica gel (gradient CHCl_3_-MeOH) and the crude product further purified by preparative HPLC.

##### [2*S*,4*R*]-1-*N*-*tert*-Butyloxycarbonyl-4-dimethoxytrityloxy-2-hydroxymethylpyrrolidine (**6a**)

To a solution of [2*S*,4*R*]-1-*N*-*tert*-butyloxycarbonyl-2-(*tert*-butyldimethylsilyloxy)methyl-4-hydroxypyrrolidine (26.5 g, 80 mmol) in dry pyridine (800 mL), dimethoxytrityl chloride (32.5 g, 96 mmol) was added. The mixture was stirred overnight at room temperature. The reaction was quenched by the addition of dry MeOH (10 mL), and the reaction mixture was concentrated, diluted with ethyl acetate (500 mL), and washed with brine (3 × 100 mL). The organic phase was dried over Na_2_SO_4_, filtered, evaporated, and purified by chromatography on silica gel using a linear gradient of ethyl acetate in cyclohexane. To the obtained product, a freshly prepared 0.5 M solution of TBAF in THF (mL) was added and the mixture stirred for 10 min. Dowex 50 in Et_3_NH^+^ form was added and the mixture filtered and concentrated under vacuum. Compound **6a** was obtained by chromatography on silica gel using a linear gradient of ethyl acetate in toluene in 86% yield (35.8 g) in the form of a yellow foam.

^1^H NMR (500.0 MHz, DMSO-*d*_6_, *T* = 80 °C): 1.35 (s, 9H, (CH_3_)_3_C); 1.80 (ddd, 1H, *J*_gem_ = 12.9, *J*_3b,2_ = 6.2, *J*_3b,4_ = 4.6, H-3b); 1.86 (bddd, 1H, *J*_gem_ = 12.9, *J*_3a,4_ = 7.6, *J*_3a,2_ = 6.0, H-3a); 2.69, 2.77 (2 × bm, 2 × 1H, H-5); 3.26 (dt, 1H, *J*_gem_ = 10.6, *J*_Hb,2_ = *J*_Hb,OH_ = 5.5, C**H**_**b**_H_a_O); 3.32 (bddd, 1H, *J*_gem_ = 10.6, *J*_Ha,OH_ = 4.7, *J*_Ha,2_ = 3.8, CH_b_**H**_**a**_O); 3.756, 3.759 (2 × s, 2 × 3H, 2 × CH_3_O); 3.79 (bm, 1H, H-2); 4.19 (m, 1H, H-4); 4.33 (bdd, 1H, *J* = 5.5, 4.7, OH); 6.87–6.92 (m, 4H, H-*m*-C_6_H_4_OMe-DMTr); 7.23 (m, 1H, H-*p*-C_6_H_5_-DMTr); 7.25–7.30 (m, 4H, H-*o*-C_6_H_4_OMe-DMTr); 7.30–7.33 (m, 2H, H-*m*-C_6_H_5_-DMTr); 7.38–7.41 (m, 2H, H-*o*-C_6_H_5_-DMTr).

^13^C NMR (125.7 MHz, DMSO-*d*_6_, *T* = 80 °C): 27.79 ((**C**H_3_)_3_C); 35.07 (b, CH_2_–3); 52.22 (CH_2_–5); 54.79 (CH_3_O–DMTr); 56.94 (CH-2); 62.09 (CH_2_O); 71.22 (b, CH-4); 77.84 ((CH_3_)_3_**C**); 85.69 (C-DMTr); 112.99, 113.02 (CH-*m*-C_6_H_4_OMe-DMTr); 126.31 (CH-*p*-C_6_H_5_-DMTr); 127.39 (CH-*m*-C_6_H_5_-DMTr); 127.47 (CH-*o*-C_6_H_5_-DMTr); 129.36, 129.39 (CH-*o*-C_6_H_4_OMe-DMTr); 136.12, 136.27 (C-*i*-C_6_H_4_OMe-DMTr); 145.22 (C-*i*-C_6_H_5_-DMTr); 153.46 (CO); 158.01 (C-*p*-C_6_H_4_OMe-DMTr).

**IR** ν_max_ (CHCl_3_) 3626 (vw), 3361 (w), 3087 (w), 3061 (w), 2980 (m), 2957 (m), 2936 (m), 2839 (m), 1687 (s, sh), 1662 (s), 1608 (s), 1582 (w), 1509 (vs), 1495 (m), 1478 (m), 1464 (s), 1456 (s), 1446 (s), 1442 (s), 1412 (vs), 1393 (s, sh), 1368 (s), 1340 (m), 1302 (m), 1252 (vs), 1179 (vs), 1175 (vs), 1154 (s), 1117 (m), 1081 (s), 1070 (m, sh), 1035 (s), 1011 (m), 1002 (vw), 912 (w), 836 (m), 830 (s), 705 (m), 700 (m), 638 (w), 612 (w), 595 (m), 584 (m), 466 (w).

**HR-ESI** C_31_H_37_O_6_NNa (M+Na)^+^ calcd 542.25131, found 542.25101.

##### [2*R*,4*S*]-1-*N*-*tert*-Butyloxycarbonyl-4-dimethoxytrityloxy-2-hydroxymethylpyrrolidine (**6b**)

This compound was prepared using the same procedure as for its enantiomer **6a**. All the spectra were identical to those for **6a**.

##### [2*S*,4*R*]-1-*N*-*tert*-Butyloxycarbonyl-4-hydroxy-2-(diethylphosphono)ethoxymethyl-pyrrolidine (**7a**)

Compound **7a** was prepared from **6a** (7 g, 13.5 mmol) according to general procedure **A**. Yield 3.95 g, 77%.

^1^H NMR (500.0 MHz, DMSO-*d*_6_, *T* = 80 °C): 1.24 (t, 6H, *J*_vic_ = 7.0, C**H**_**3**_CH_2_O); 1.42 (s, 9H, (CH_3_)_3_C); 1.86 (ddd, 1H, *J*_gem_ = 13.0, *J*_3b,2_ = 7.9, *J*_3b,4_ = 4.4, H-3b); 1.96 (ddd, 1H, *J*_gem_ = 13.0, *J*_3a,4_ = 7.6, *J*_3a,2_ = 6.0, H-3a); 2.01 (dt, 2H, *J*_H,P_ = 18.3, *J*_vic_ = 7.2, PC**H**_**2**_CH_2_O); 3.21–3.30 (m, 2H, H-5); 3.44 (dd, 1H, *J*_gem_ = 9.6, *J*_Hb,2_ = 6.1, C**H**_**b**_H_a_O); 3.51 (dd, 1H, *J*_gem_ = 9.6, *J*_Ha,2_ = 3.2, CH_b_**H**_**a**_O); 3.60 (dt, 2H, *J*_H,P_ = 13.3, *J*_vic_ = 7.2, PC**H**_**2**_CH_2_O); 3.89 (dddd, 1H, *J*_2,3_ = 7.9, 6.0, *J*_2,CH2_ = 6.1, 3.2, H-2); 3.94–4.06 (m, 4H, C**H**_**3**_CH_2_O); 4.24 (m, 1H, H-4); 4.69 (bs, 1H, OH).

^13^C NMR (125.7 MHz, DMSO-*d*_6_, *T* = 80 °C): 15.98 (d, *J*_C,P_ = 5.6, **C**H_3_CH_2_O); 26.27 (d, *J*_C,P_ = 137.4, P**C**H_2_CH_2_O); 28.04 ((CH_3_)_3_C); 37.24 (CH_2_–3); 54.56 (CH_2_–5); 55.23 (CH-2); 60.77 (d, *J*_C,P_ = 6.2, CH_3_**C**H_2_O); 64.67 (PCH_2_**C**H_2_O); 67.80 (C-4); 71.09 (CH_2_O); 78.19 ((CH_3_)_3_C); 153.72 (CO).

^31^P[^1^H] NMR (202.3 MHz, DMSO-*d*_6_, *T* = 80 °C): 28.12.

**IR** ν_max_ (CHCl_3_) 3613 (w), 3401 (w, vbr), 2984 (s), 2932 (m), 2874 (m), 1686 (vs), 1478 (m), 1455 (m), 1443 (m), 1405 (vs), 1393 (vs, sh), 1368 (s), 1246 (s, br), 1165 (s), 1099 (s, sh), 1055 (vs), 1030 (vs), 963 (s), 463 (vw).

**HR-ESI** C_16_H_33_O_7_NP (M+H)^+^ calcd 382.19892, found 382.19906; C_16_H_32_O_7_NPNa (M+Na)^+^ calcd 404.18086, found 404.18095.

##### [2*R*,4*S*]-1-*N*-*tert*-Butyloxycarbonyl-4-hydroxy-2-(diethyl phosphono)ethoxymethyl-pyrrolidine (**7b**)

This compound was prepared using the same procedure as for its enantiomer **7a**. All the spectra were identical to those for **7a**.

##### [2*S*,4*S*]-4-Guanin-9-yl-2-(2-(diethyl phosphono)ethoxymethyl)pyrrolidine (**8a**)

**8a** was prepared from **7a** (2.97 g, 7.79 mmol) in the presence of triphenylphosphine (4.5 g, 27.27 mmol) according to general procedures **C** and **D**. Yield 1.27 g, 39%.

^1^H NMR (500.0 MHz, DMSO-*d*_6_): 1.209, 1.212 (2 × t, 2 × 3H, *J*_vic_ = 7.3, C**H**_**3**_CH_2_O); 1.67 (ddd, 1H, *J*_gem_ = 13.3, *J*_3′b,2′_ = 8.1, *J*_3′b,4′_ = 6.5, H-3′b); 1.99–2.13 (m, 2H, PC**H**_**2**_CH_2_O); 2.38 (dd, 1H, *J*_gem_ = 13.3, *J*_3′a,4′_ = 8.4, *J*_3′a,2′_ = 7.8, H-3′a); 3.00 (dd, 1H, *J*_gem_ = 11.1, *J*_5′b,4′_ = 4.8, H-5′b); 3.14 (dd, 1H, *J*_gem_ = 11.1, *J*_5′a,4′_ = 6.9, H-5′a); 3.28 (m, 1H, H-2′); 3.42 (dd, 1H, *J*_gem_ = 9.6, *J*_Hb,2′_ = 5.3, C**H**_**b**_H_a_O); 3.46 (dd, 1H, *J*_gem_ = 9.6, *J*_Ha,2′_ = 6.3, CH_b_**H**_**a**_O); 3.61 (dt, 2H, *J*_H,P_ = 13.0, *J*_vic_ = 7.3, PCH_2_C**H**_**2**_O); 3.92–4.03 (m, 4H, CH_3_C**H**_**2**_O); 4.73 (dddd, 1H, *J*_4′,3′_ = 8.4, 6.5, *J*_4′,5′_ = 6.9, 4.8, H-4′); 6.43 (bs, 2H, NH_2_); 7.81 (s, 1H, H-8).

^13^C NMR (125.7 MHz, DMSO-*d*_6_): 16.26 (d, *J*_C,P_ = 5.7, **C**H_3_CH_2_O); 25.94 (d, *J*_C,P_ = 136.9, P**C**H_2_CH_2_O); 35.44 (CH_2_–3′); 52.16 (CH_2_–5′); 53.78 (CH-4′); 57.07 (CH-2′); 60.98 (d, *J*_C,P_ = 6.1, CH_3_**C**H_2_O); 64.54 (PCH_2_**C**H_2_O); 73.43 (CH_2_O); 116.59 (C-5); 135.58 (CH-8); 150.88 (C-4); 153.34 (C-2); 156.84 (C-6).

^31^P[^1^H] NMR (202.4 MHz, DMSO-*d*_6_): 29.81.

##### [2*R*,4*R*]-4-Guanin-9-yl-2-(2-(diethyl phosphono)ethoxymethyl)pyrrolidine (**8b**)

This compound was prepared using the same procedure as for its enantiomer **8a**. All the spectra were identical to those for **8a**.

##### [2*S*,4*S*]-4-Guanin-9-yl-2-(2-phosphonoethoxymethyl)-1-*N*-(3-phosphonopropionyl)pyrrolidine (**4**)

Compound **4** was prepared from **8a** (0.40 g, 0.97 mmol) and diisopropyl phosphonopropionic acid according to general procedures **E** and **F**. Yield 220 mg, 39%.
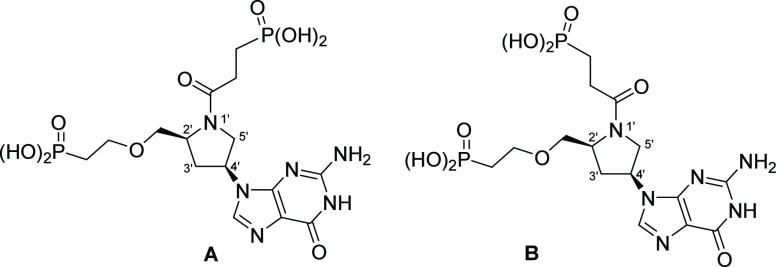


A:B ∼ 3:1

^1^H NMR (500.2 MHz, D_2_O, ref(dioxan) = 3.75 ppm): 1.77–2.00 (m, 8H, PC**H**_**2**_CH_2_O-A,B, PC**H**_**2**_CH_2_CO-A,B); 2.39–2.52 (m, 2H, H-3′b-A,B); 2.57–2.80 (m, 5H, H-3′a-A, PCH_2_C**H**_**2**_CO-A,B); 2.84 (dt, 1H, *J*_gem_ = 13.7, *J*_3′a,2′_ = *J*_3′a,4′_ = 8.4, H-3′a-B); 3.59 (dd, 1H, *J*_gem_ = 10.6, *J*_vic_ = 6.0, CH_a_**H**_**b**_O–B); 3.65–3.79 (m, 8H, H-5′b-B, C**H**_**a**_H_b_O–B, CH_2_O-A, PCH_2_C**H**_**2**_O-A,B); 3.89 (dd, 1H, *J*_gem_ = 11.1, *J*_5′b,4′_ = 8.8, H-5′b-A); 4.30 (dd, 1H, *J*_gem_ = 11.1, *J*_5′a,4′_ = 7.4, H-5′a-A); 4.35 (m, 1H, H-2′-A); 4.42 (dd, 1H, *J*_gem_ = 12.4, *J*_5′a,4′_ = 7.5, H-5′a-B); 4.49 (m, 1H, H-2′-B); 4.85 (m, 1H, H-4′-B); 4.90 (m, 1H, H-4′-A); 7.93 (s, 1H, H-8-B); 7.94 (s, 1H, H-8-A).

^13^C NMR (125.8 MHz, D_2_O, ref(dioxan) = 69.30 ppm): 25.97 (d, *J*_C,P_ = 134.8, P**C**H_2_CH_2_CO-A); 26.51 (d, *J*_C,P_ = 134.9, P**C**H_2_CH_2_CO-B); 30.53 (d, *J*_C,P_ = 2.0, PCH_2_**C**H_2_CO-A); 31.50 (d, *J*_C,P_ = 129.5, P**C**H_2_CH_2_O-A,B); 32.14 (d, *J*_C,P_ = 2.3, PCH_2_**C**H_2_CO-A); 35.18 (CH_2_–3′-A); 36.17 (CH_2_–3′-B); 52.02 (CH_2_–5′-B); 53.84 (CH_2_–5′-A); 54.17 (CH-4′-B); 54.80 (CH-4′-A); 58.61 (CH-2′-A); 59.00 (CH-2′-B); 69.66 (d, *J*_C,P_ = 1.4, PCH_2_**C**H_2_O-A); 69.70 (d, *J*_C,P_ = 1.4, PCH_2_**C**H_2_O–B); 72.32 (CH_2_O-A); 74.43 (CH_2_O–B); 118.85 (C-5-A); 118.94 (C-5-B); 140.42 (CH-8-A,B); 154.45 (C-4-A,B); 156.26 (C-2-B); 156.30 (C-2-A); 161.56 (C-6-A,B); 177.01 (d, *J*_C,P_ = 18.1, NCO-A); 177.60 (d, *J*_C,P_ = 17.5, NCO-B).

^31^P[^1^H] NMR (202.5 MHz, D_2_O): 21.26 (PCH_2_CH_2_CO-B); 21.35 (PCH_2_CH_2_CO-A); 24.27 (PCH_2_CH_2_O–B); 24.38 (PCH_2_CH_2_O-A).

**IR** ν_max_ (KBr) 3307 (m, br), 3113 (m, br), 2782 (m, vbr), 2368 (w, vbr), 1693 (vs), 1637 (s, br), 1613 (s, sh), 1575 (w, sh), 1534 (w), 1478 (w), 1416 (w), 1320 (w), 1070 (m, br), 898 (m, br), 781 (w), 695 (w), 640 (vw).

**HR-ESI** C_15_H_23_O_9_N_6_P_2_ (M-H)^−^ calcd 493.10072, found 493.09988.

[α]_D_^20^ −11.4 (c 0.342, H_2_O)

##### [2*R*,4*R*]-4-Guanin-9-yl-2-(2-phosphonoethoxymethyl)-1-*N*-(3-phosphonopropionyl)pyrrolidine (**3**)

Compound **3** was prepared from **8b** (0.15 g, 0.36 mmol) and diisopropyl phosphonopropionic acid according to general procedures **E** and **F**. Yield 15 mg, 7%. All the spectra were identical to those for **4**.

##### [2*S*,4*S*]-1-*N*-*tert*-Butyloxycarbonyl-4-hydroxy-2-(diethyl phosphono)ethoxymethylpyrrolidine (**9a**)

Compound **9a** was prepared from **7a** (1.95g, 4.8 mmol) according to general procedure **B**. Yield 1.36 g, 69%.

^**1**^**H NMR** (500.0 MHz, DMSO-*d*_6_): 1.25 (t, 6H, *J*_vic_ = 7.0, C**H**_**3**_CH_2_O); 1.42 (s, 9H, (CH_3_)_3_C); 1.84 (dtd, 1H, *J*_gem_ = 13.3, *J*_3b,2_ = *J*_3b,4_ = 3.5, *J*_3b,5b_ = 1.2, H-3b); 1.98–2.09 (m, 3H, H-3a, PC**H**_**2**_CH_2_O); 3.06 (ddd, 1H, *J*_gem_ = 11.3, *J*_5b,4_ = 3.5, *J*_5b,3b_ = 1.2, H-5b); 3.48 (dd, 1H, *J*_gem_ = 11.3, *J*_5a,4_ = 5.5, H-5a); 3.54 (dd, 1H, *J*_gem_ = 9.2, *J*_Hb,2_ = 7.9, CH_a_**H**_**b**_O); 3.57–3.68 (m, 3H, CH_a_**H**_**b**_O, OC**H**_**2**_CH_2_P); 3.81 (tt, 1H, *J*_2,3_ = 7.9, 3.5, *J*_2,CH2_ = 7.9, 3.5, H-2); 3.95–4.08 (m, 4H, CH_3_C**H**_**2**_O); 4.20 (tt, 1H, *J*_4,3_ = 5.5, 3.5, *J*_4,5_ = 5.5, 3.5, H-4); 4.69 (s, 1H, OH).

^**13**^**C NMR** (125.7 MHz, DMSO-*d*_6_): 15.92 (d, *J*_C,P_ = 5.6, **C**H_3_CH_2_O); 26.26 (d, *J*_C,P_ = 137.3, P**C**H_2_CH_2_O); 27.96 ((**C**H_3_)_3_C); 36.23 (CH_2_–3); 54.62 (CH_2_–5); 55.45 (CH-2); 60.74 (d, *J*_C,P_ = 6.3, CH_3_**C**H_2_O); 64.38 (d, *J*_C,P_ = 1.2, O**C**H_2_CH_2_P); 68.42 (CH-4); 71.27 (CH_2_O); 78.22 ((CH_3_)_3_**C**); 153.48 (CO).

^**31**^**P[**^**1**^**H] NMR** (202.3 MHz, DMSO-*d*_6_): 28.02.

**IR** ν_max_ (CHCl_3_) 3610 (vw), 3421 (m, br), 2983 (s), 2939 (m), 2875 (m), 1686 (vs), 1478 (m), 1469 (m), 1456 (m), 1444 (m), 1401 (vs, sh), 1394 (vs), 1367 (s), 1246 (s), 1167 (s), 1111 (s), 1088 (s), 1053 (vs), 1029 (vs), 963 (s), 463 (vw).

**HR-ESI** C_16_H_33_O_7_NP (M+H)^+^ calcd 382.19892, found 382.19871; C_16_H_32_O_7_NPNa (M+Na)^+^ calcd 404.18086, found 404.18061.

##### [2*R*,4*R*]-1-*N*-*tert*-Butyloxycarbonyl-4-hydroxy-2-(diethylphosphono)ethoxymethylpyrrolidine (**9b**)

This compound was prepared using the same procedure as for its enantiomer **9a**. All the spectra were identical to those for **9a**.

##### [2*S*,4*R*]-4-Guanin-9-yl-2-(2-(diethyl phosphono)ethoxymethyl)pyrrolidine (**10a**)

Compound **10a** was prepared from **9a** (2 g, 5.24 mmol) according to general procedures **C** and **D**. Yield 0.94 g, 43%.

^1^H NMR (500.0 MHz, DMSO-*d*_6_): 1.22 (t, 6H, *J*_vic_ = 7.1, C**H**_**3**_CH_2_O); 1.94 (ddd, 1H, *J*_gem_ = 13.7, *J*_3′b,4′_ = 8.1, *J*_3′b,2′_ = 7.0, H-3′b); 2.02–2.10 (m, 3H, H-3′a, PC**H**_**2**_CH_2_O); 2.98 (dd, 1H, *J*_gem_ = 11.3, *J*_5′b,4′_ = 4.6, H-5′b); 3.22 (dd, 1H, *J*_gem_ = 11.3, *J*_5′a,4′_ = 6.3, H-5′a); 3.32 (dd, 1H, *J*_gem_ = 9.5, *J*_Hb,2′_ = 5.8, C**H**_**b**_H_a_O); 3.35 (dd, 1H, *J*_gem_ = 9.5, *J*_Ha,2′_ = 6.1, CH_b_**H**_**a**_O); 3.55 (m, 1H, H-2′); 3.60 (dt, 2H, *J*_H,P_ = 13.2, *J*_vic_ = 7.3, PCH_2_C**H**_**2**_O); 3.93–4.03 (m, 4H, CH_3_C**H**_**2**_O); 4.74 (m, 1H, H-4′); 6.43 (bs, 2H, NH_2_); 7.76 (s, 1H, H-8).

^13^C NMR (125.7 MHz, DMSO-*d*_6_): 16.28 (d, *J*_C,P_ = 5.9, **C**H_3_CH_2_O); 25.93 (d, *J*_C,P_ = 136.9, P**C**H_2_CH_2_O); 35.16 (CH_2_–3′); 52.05 (CH_2_–5′); 54.24 (CH-4′); 56.31 (CH-2′); 61.00 (d, *J*_C,P_ = 6.1, CH_3_**C**H_2_O); 64.54 (PCH_2_**C**H_2_O); 73.52 (CH_2_O); 116.73 (C-5); 135.70 (CH-8); 150.88 (C-4); 153.32 (C-2); 156.85 (C-6).

^31^P[^1^H] NMR (202.4 MHz, DMSO-*d*_6_): 29.84.

**IR** ν_max_ (CHCl_3_) 3475 (w, vbr, sh), 3316 (w, vbr), 3130 (m, vbr), 2930 (m), 1692 (vs), 1658 (m), 1620 (w), 1576 (w), 1534 (w), 1479 (w), 1406 (w, sh), 1393 (w), 1376 (w), 1245 (w, sh), 1099 (vw), 1056 (m), 1029 (m), 964 (w), 638 (vw).

**HR-ESI** C_16_H_28_O_5_N_6_P (M+H)^+^ calcd 415.18533, found 415.18507; C_16_H_27_O_5_N_6_PNa (M+Na)^+^ calcd 437.16728, found 437.16689.

##### [2*R*,4*S*]-4-Guanin-9-yl-2-(2-(diethyl phosphono)ethoxymethyl)pyrrolidine (**10b**)

The titled compound was prepared using the same procedure as for its enantiomer **10a**. All the spectra were identical to those for **10a**.

##### [2*R*,4*S*]-4-Guanin-9-yl-2-(2-phosphonoethoxymethyl)-1-*N*-(3-phosphonopropionyl)pyrrolidine (**2**)

Compound **2** was prepared from **10b** (0.42 g, 1.00 mmol) and diisopropyl phosphonopropionic acid according to general procedures **E** and **F**. Yield 280 mg, 48%.
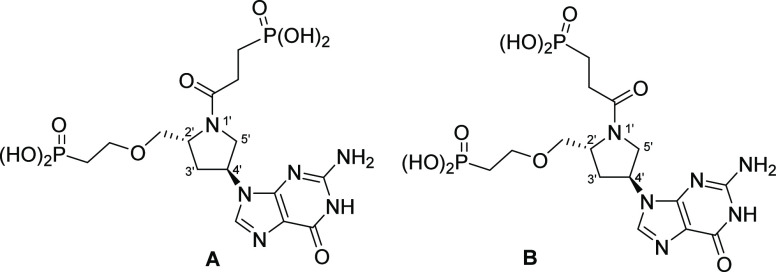


A:B ∼ 8:3

^1^H NMR (500.0 MHz, D_2_O, ref(dioxan) = 3.75 ppm): 1.67–1.88 (m, 4H, PC**H**_**2**_CH_2_CO-A,B); 1.92–2.09 (m, 4H, PC**H**_**2**_CH_2_O-A,B); 2.46–2.84 (m, 8H, H-3′-A,B, PCH_2_C**H**_**2**_CO-A,B); 3.64–3.84 (m, 8H, CH_2_O-A,B, PCH_2_C**H**_**2**_O-A,B); 3.87–3.94 (m, 3H, H-5′b-A, H-5′-B); 4.15 (dd, 1H, *J*_gem_ = 11.3, *J*_5′a,4′_ = 7.4, H-5′a-A); 4.51 (m, 1H, H-2′-A); 4.60 (m, 1H, H-2′-B); 5.21–5.30 (m, 2H, H-4′-A,B); 7.84 (s, 2H, H-8-A,B).

^13^C NMR (125.7 MHz, D_2_O, ref(dioxan) = 69.30 ppm): 26.04 (d, *J*_C,P_ = 134.3, P**C**H_2_CH_2_CO-A); 26.67 (d, *J*_C,P_ = 133.9, P**C**H_2_CH_2_CO-B); 30.88 (PCH_2_**C**H_2_CO-B); 31.86 (d, *J*_C,P_ = 128.7, P**C**H_2_CH_2_O-A,B); 32.07 (PCH_2_**C**H_2_CO-A); 35.51 (CH_2_–3′-A); 36.70 (CH_2_–3′-B); 53.25 (CH_2_–5′-B); 54.46 (CH_2_–5′-A); 54.62 (CH-4′-B); 55.47 (CH-4′-A); 58.95 (CH-2′-A); 59.78 (CH-2′-B); 70.16 (PCH_2_**C**H_2_O-A); 70.25 (PCH_2_**C**H_2_O–B); 72.62 (CH_2_O-A); 74.28 (CH_2_O–B); 118.98 (C-5-A); 119.04 (C-5-B); 140.63 (CH-8-A,B); 154.31 (C-4-A,B); 156.32 (C-2-B); 156.35 (C-2-A); 161.78 (C-6-A,B); 177.08 (d, *J*_C,P_ = 18.5, NCO-A); 177.41 (d, *J*_C,P_ = 18.5, NCO-B).

^31^P{^1^H} NMR (202.4 MHz, D_2_O): 20.81 (PCH_2_CH_2_CO-B); 20.89 (PCH_2_CH_2_CO-A); 23.68 (PCH_2_CH_2_O-A,B).

**IR** ν_max_ (KBr) 3433 (s), 3112 (m), 2924 (m), 2855 (m, w), 1693 (vs), 1628 (s), 1536 (w), 1480 (w), 1176 (m), 1074 (m, br), 896 (w, br), 783 (w), 694 (w), 641 (w).

**HR-ESI** C_15_H_23_O_9_N_6_P_2_ (M-H)^−^ calcd 493.10072, found 493.10061.

[α]_D_^20^ +13.9 (c 0.375, H_2_O)

##### [2*S*,4*R*]-4-Guanin-9-yl-2-(2-phosphonoethoxymethyl)-1-*N*-(3-phosphonopropionyl)pyrrolidine (**5**)

Compound **5** was prepared from **10a** (341 mg, 0.82 mmol) and diisopropyl phosphonopropionic acid according to general procedures **E** and **F**. Yield 138 mg, 29%. All the spectra were identical to those for **2**.

[α]_D_^20^ −11.4 (c 0.395, H_2_O)

##### [2*S*,4*R*]-1-*N*-*tert*-Butyloxycarbonyl-2-dimethoxytrityloxymethyl-4-hydroxypyrrolidine (**11**)

To a solution of [2*S*,4*R*]**-**1-*N*-*tert*-butyloxycarbonyl-4-hydroxy-2-(hydroxymethyl)pyrrolidine^57^ (50 g, 230 mmol) in dry pyridine (1,000 mL), dimethoxytrityl chloride (97 g, 253 mmol) was added. The mixture was stirred overnight at room temperature. The reaction was quenched by the addition of dry MeOH (10 mL), and the reaction mixture was concentrated, diluted with chloroform (500 mL), and washed with brine (3 × 100 mL). The organic phase was dried over Na_2_SO_4_, filtered, evaporated, and purified by chromatography on silica gel using a linear gradient of toluene in ethyl acetate. Compound **11** was obtained in 85% yield (111.7 g) in the form of a yellow foam.

^1^H NMR (500.0 MHz, DMSO-*d*_6_, *T* = 80 °C): 1.31 (bs, 9H, (CH_3_)_3_C); 1.95 (bm, 1H, H-3b); 2.03 (bm, 1H, H-3a); 2.99–3.01 (bm, 2H, CH_2_O); 3.29–3.39 (bm, 2H, H-5); 3.75 (s, 6H, CH_3_O); 3.97 (bm, 1H, H-2); 4.31 (bm, 1H, H-4); 4.69 (bm, 1H, OH); 6.85–6.90 (m, 4H, H-*m*-C_6_H_4_OMe-DMTr); 7.19–7.26 (m, 5H, H-*o*-C_6_H_4_OMe-DMTr, H-*p*-C_6_H_5_-DMTr); 7.28–7.32 (m, 2H, H-*m*-C_6_H_5_-DMTr); 7.34–7.38 (m, 2H, H-*o*-C_6_H_5_-DMTr).

^13^C NMR (125.7 MHz, DMSO-*d*_6_, *T* = 80 °C): 27.77 ((**C**H_3_)_3_C); 38.10 (b, CH_2_–3); 54.48 (b, CH_2_–5); 54.76 (CH_3_O–DMTr); 55.91 (b, CH-2); 55.29 (CH_3_O–DMTr); 64.01 (b, CH_2_O); 67.75 (b, CH-4); 77.88 ((CH_3_)_3_**C**); 85.01 (C-DMTr); 112.90 (CH-*m*-C_6_H_4_OMe-DMTr); 126.22 (CH-*p*-C_6_H_5_-DMTr); 127.32 (CH-*m*-C_6_H_5_-DMTr); 127.39 (CH-*o*-C_6_H_5_-DMTr); 129.25, 129.26 (CH-*o*-C_6_H_4_OMe-DMTr); 135.61, 135.68 (C-*i*-C_6_H_4_OMe-DMTr); 144.66 (C-*i*-C_6_H_5_-DMTr); 153.47 (CO); 157.86 (C-*p*-C_6_H_4_OMe-DMTr).

**IR** ν_max_ (KBr) 3430 (m, vbr), 3087 (w, sh), 3058 (w), 3035 (w), 2974 (m), 2872 (m), 2836 (m), 1694 (vs), 1672 (s), 1608 (s), 1583 (m), 1509 (vs), 1478 (m), 1446 (s), 1407 (s), 1393 (s, sh), 1366 (s), 1302 (s), 1251 (vs), 1175 (vs), 1161 (s, sh), 1115 (m), 1104 (m), 1086 (m, sh), 1074 (m), 1055 (m), 1035 (s), 1012 (m, sh), 914 (w), 829 (s), 772 (m), 727 (m), 702 (m), 635 (w), 596 (m), 585 (m), 548 (w), 465 w.

**HR-ESI** C_31_H_37_O_6_NNa (M+Na)^+^ calcd 542.25131, found 542.25110.

##### [2*S*,4*S*]-1-*N*-*tert*-Butyloxycarbonyl-2-dimethoxytrityloxymethyl-4-hydroxypyrrolidine (**12**)

Compound **12** was prepared from **11** (10.1 g, 19.4 mmol) according to general procedure **B**. Yield 8.05 g, 80%.

^1^H NMR (500.0 MHz, DMSO-*d*_6_, *T* = 80 °C): 1.29 (bs, 9H, (CH_3_)_3_C); 1.97 (bm, 1H, H-3b); 2.16 (bddd, 1H, *J*_gem_ = 13.7, *J*_3a,4_ = 8.3, *J*_3a,2_ = 6.2, H-3a); 3.01 (dd, 1H, *J*_gem_ = 11.2, *J*_5b,4_ = 4.2, H-5b); 3.16 (dd, 1H, *J*_gem_ = 8.3, *J*_Hb,2_ = 7.6, C**H**_**b**_H_a_O); 3.25 (dd, 1H, *J*_gem_ = 8.3, *J*_Ha,2_ = 4.5, CH_b_**H**_**a**_O); 3.55 (dd, 1H, *J*_gem_ = 11.2, *J*_5a,4_ = 5.8, H-5a) 3.755 (s, 6H, CH_3_O); 3.90 (m, 1H, H-2); 4.20 (bm, 1H, H-4); 4.70 (bm, 1H, OH); 6.85–6.89 (m, 4H, H-*m*-C_6_H_4_OMe-DMTr); 7.21 (m, 1H, H-*p*-C_6_H_5_-DMTr); 7.24–7.32 (m, 6H, H-*o*-C_6_H_4_OMe-DMTr, H-*m*-C_6_H_5_-DMTr); 7.38–7.41 (m, 2H, H-*o*-C_6_H_5_-DMTr).

^13^C NMR (125.7 MHz, DMSO-*d*_6_, *T* = 80 °C): 27.72 ((**C**H_3_)_3_C); 36.30 (b, CH_2_–3); 54.08 (CH_2_–5); 54.75 (CH_3_O–DMTr); 55.65 (CH-2); 64.19 (CH_2_O); 67.98 (b, CH-4); 77.94 ((CH_3_)_3_**C**); 85.04 (C-DMTr); 112.83, 112.84 (CH-*m*-C_6_H_4_OMe-DMTr); 128.15 (CH-*p*-C_6_H_5_-DMTr); 127.24 (CH-*m*-C_6_H_5_-DMTr); 127.49 (CH-*o*-C_6_H_5_-DMTr); 129.34, 129.36 (CH-*o*-C_6_H_4_OMe-DMTr); 135.79, 135.82 (C-*i*-C_6_H_4_OMe-DMTr); 144.81 (C-*i*-C_6_H_5_-DMTr); 153.20 (CO); 157.82 (C-*p*-C_6_H_4_OMe-DMTr).

**IR** ν_max_ (CHCl_3_) 3612 (w), 3421 (m, br), 3086 (w), 3061 (w), 2977 (s), 2960 (s, sh), 2937 (s), 2911 (m), 2880 (w, sh), 2839 (m), 1686 (vs), 1609 (s), 1584 (m), 1580 (m, sh), 1510 (vs), 1493 (m), 1465 (s), 1478 (m), 1447 (s), 1442 (s), 1401 (vs), 1392 (s), 1367 (s), 1340 (m), 1252 (vs), 1177 (vs), 1167 (s, sh), 1155 (s, sh), 1124 (s), 1088 (s), 1055 (s, sh), 1037 (vs), 1013 (m), 1005 (m), 936 (w), 915 (w), 830 (s), 704 (m), 635 (vw), 629 (w), 619 (vw), 596 (m), 585 (s), 461 (vw).

**HR-ESI** C_31_H_37_O_6_NNa (M+Na)^+^ calcd 542.25131, found 542.25126.

##### [2*S*,4*R*]-4-Guanin-9-yl-2-hydroxymethylpyrrolidine (**13**)

Compound **13** was prepared from **12** (2.43 g, 4.68 mmol) in the presence of diphenylpyridylphosphine (2.46 g, 9.35 mmol) according to general procedures **C** and **D**. Yield 0.66 g, 56.8%.

^1^H NMR (500.0 MHz, DMSO-*d*_6_): 1.97 (ddd, 1H, *J*_gem_ = 13.5, *J*_3′b,4′_ = 8.1, *J*_3′b,2′_ = 6.6, H-3′b); 2.02 (ddd, 1H, *J*_gem_ = 13.5, *J*_3′a,2′_ = 7.8, *J*_3′a,4′_ = 4.8, H-3′a); 2.97 (dd, 1H, *J*_gem_ = 11.2, *J*_5′b,4′_ = 4.8, H-5′b); 3.23 (dd, 1H, *J*_gem_ = 11.2, *J*_5′a,4′_ = 6.4, H-5′a); 3.32 (dd, 1H, *J*_gem_ = 10.6, *J*_Hb,2′_ = 5.6, C**H**_**b**_H_a_O); 3.36 (dd, 1H, *J*_gem_ = 10.3, *J*_Ha,2′_ = 5.4, CH_b_**H**_**a**_O); 3.42 (dddd, 1H, *J*_2′,3′_ = 7.8, 6.6, *J*_2′,CH2_ = 5.6, 5.4, H-2′); 4.74 (ddt, 1H, *J*_4′,3′_ = 8.1, 4.8, *J*_4′,5′_ = 6.4, 4.8, H-4′); 6.45 (bs, 2H, NH_2_); 7.77 (s, 1H, H-8).

^13^C NMR (125.7 MHz, DMSO-*d*_6_): 34.87 (CH_2_–3′); 52.13 (CH_2_–5′); 54.31 (CH-2′); 58.72 (CH-4′); 64.13 (CH_2_O); 116.69 (C-5); 135.65 (CH-8); 150.92 (C-4); 153.35 (C-2); 156.89 (C-6).

**IR** ν_max_ (KBr) 3495 (s), 3424 (s, br, sh), 3319 (s, br), 3192 (s, br), 3125 (s), 2910 (s, sh), 2883 (s), 2825 (s, sh), 2848 (s), 2786 (s), 2733 (s, br), 1727 (s), 1689 (s), 1632 (vs), 1606 (s), 1536 (s), 1485 (s), 1388 (s), 1570 (s), 1329 (m), 1051 (m), 777 (m), 733 (w), 676 (m), 631 (w, br).

**HR-ESI** C_10_H_15_O_2_N_6_ (M+H)^+^ calcd 251.12510, found 251.12488; C_10_H_14_O_2_N_6_Na (M+Na)^+^ calcd 273.10704, found 273.10688.

##### [2*S*,4*R*]-4-Guanin-9-yl-2-hydroxymethyl-1-*N*-(3-phosphonopropionyl)pyrrolidine (**1**)

Compound **1** was prepared from **13** (106 mg, 0.42 mmol) and diisopropyl phosphonopropionic acid according to general procedures **E** and **F**. Yield 141 mg, 78%.
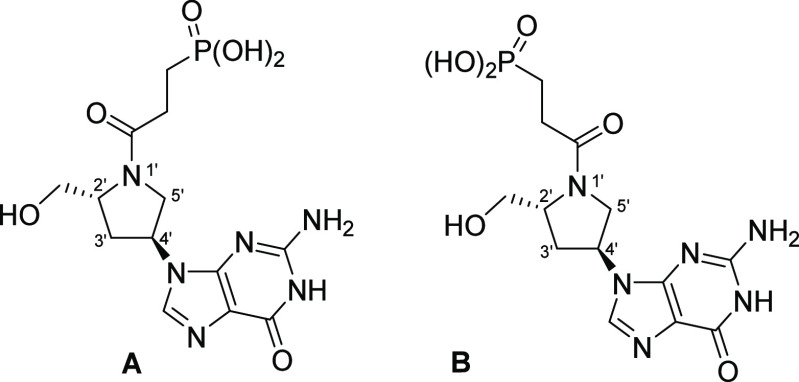


A:B ∼ 2:1

^1^H NMR (500.0 MHz, D_2_O, ref(*t*BuOH) = 1.24 ppm): 1.71–1.92 (m, 4H, PC**H**_**2**_CH_2_CO-A,B); 2.48–2.81 (m, 8H, H-3′-A,B, PCH_2_C**H**_**2**_CO-A,B); 3.68 (dd, 1H, *J*_gem_ = 11.7, *J*_vic_ = 3.2, CH_a_**H**_**b**_OH-A); 3.72 (dd, 1H, *J*_gem_ = 12.2, *J*_vic_ = 4.9, CH_a_**H**_**b**_OH-B); 3.82 (dd, 1H, *J*_gem_ = 12.2, *J*_vic_ = 5.2, C**H**_**a**_H_b_OH-B); 3.86–3.98 (m, 4H, H-5b-A, H-5-B, C**H**_**a**_H_b_OH-A); 4.13 (dd, 1H, *J*_gem_ = 11.5, *J*_5′a,4′_ = 7.1, H-5′a-A); 4.44 (m, 1H, H-2′-A); 4.52 (m, 1H, H-2′-B); 5.13–5.22 (m, 2H, H-4′-A,B); 7.80 (s, 1H, H-8-A); 7.81 (s, 1H, H-8-B).

^13^C NMR (125.7 MHz, D_2_O, ref(*t*BuOH) = 30.29 ppm): 23.85 (d, *J*_C,P_ = 135.0, P**C**H_2_CH_2_CO-A); 24.47 (d, *J*_C,P_ = 135.0, P**C**H_2_CH_2_CO-B); 28.68 (d, *J*_C,P_ = 2.5, PCH_2_**C**H_2_CO-B); 29.87 (d, *J*_C,P_ = 2.5, PCH_2_**C**H_2_CO-A); 33.06 (CH_2_-3′-A); 34.23 (CH_2_-3′-B); 51.36 (CH_2_-5′-B); 52.68 (CH-4′-B); 52.95 (CH_2_-5′-A); 53.62 (CH-4′-A); 58.76 (CH-2′-B); 59.40 (CH-2′-A); 62.19 (CH_2_O-A); 63.71 (CH_2_O–B); 116.87 (C-5-A); 116.93 (C-5-B); 138.35 (CH-8-A); 138.37 (CH-8-B); 152.11 (C-4-A); 152.13 (C-4-B); 154.19 (C-2-B); 154.22 (C-2-A); 156.54 (C-6-B); 159.56 (C-6-A); 174.90 (d, *J*_C,P_ = 17.7, NCO-A); 175.24 (d, *J*_C,P_ = 17.8, NCO-B).

^31^P[^1^H] NMR (202.4 MHz, D_2_O): 24.23 (P-A); 24.24 (P–B).

**IR** ν_max_ (KBr) 3401 (s, vbr), 3187 (s, br), 311 (s, br), 2953 (s, br), 2506 (m, br), 2308 (s, br), 1676 (vs, br), 1610 (vs, vbr), 1590 (vs, br), 1536 (s), 1444 (s, br), 1144 (s), 1056 (s, br), 781 (s), 726 (m), 643 (s),

**HR-ESI** C_13_H_19_O_6_N_6_PNa (M+Na)^+^ calcd 409.09959, found 409.09965; C_13_H_18_O_6_N_6_PNa_2_ (M+2Na)^+^ calcd 431.08153, found 431.08145.

##### Bis(l-phenylalanine ethyl ester) Prodrug of [2*S*,4*R*]-4-Guanin-9-yl-2-hydroxymethyl-1-*N*-(3-phosphonopropionyl)pyrrolidine **14**

Compound **14** was prepared according to general procedure G in 41% yield (0.18 g, 0.244 mmol).

A mixture of two rotamers ∼ 7:2, NMR of major rotamer:

^1^H NMR (600.1 MHz, CD_3_OD): 1.19, 1.23 (2 × t, 2 × 3H, *J*_vic_ = 7.1, C**H**_**3**_CH_2_O); 1.59–1.71 (m, 2H, PC**H**_**2**_CH_2_CO); 2.09–2.24 (m, 2H, PCH_2_C**H**_**2**_CO); 2.47 (ddd, 1H, *J*_gem_ = 12.9, *J*_3′b,4′_ = 7.3, *J*_3′b,2′_ = 3.2, H-3′b); 2.65–2.73 (m, 2H, H-3′a, H-3b-Phe); 2.83 (dd, 1H, *J*_gem_ = 13.6, *J*_3b,2_ = 8.8, H-3b-Phe); 2.96, 3.06 (2 × dd, 2 × 1H, *J*_gem_ = 13.6, *J*_3a,2_ = 5.4, H-3a-Phe); 3.59 (dd, 1H, *J*_gem_ = 11.2, *J*_6′b,2′_ = 2.9, H-6′b); 3.73 (dd, 1H, *J*_gem_ = 10.7, *J*_5′b,4′_ = 6.8, H-5′b); 3.83 (dd, 1H, *J*_gem_ = 11.2, *J*_6′a,2′_ = 4.5, H-6′a); 3.87 (m, 1H, H-2-Phe); 3.92 (dd, 1H, *J*_gem_ = 10.7, *J*_5′a,4′_ = 7.7, H-5′a; 4.04–4.18 (m, 5H, H-2-Phe, CH_3_C**H**_**2**_O); 4.35 (m, 1H, H-2′); 5.29 (m, 1H, H-4′); 7.09–7.29 (m, 10H, H-*o,m,p*-Phe); 7.80 (s, 1H, H-8).

^13^C NMR (150.9 MHz, CD_3_OD): 14.40, 14.49 (**C**H_3_CH_2_O); 24.72 (d, *J*_C,P_ = 118.2, P**C**H_2_CH_2_CO); 29.14 (d, *J*_C,P_ = 2.8, PCH_2_**C**H_2_CO); 33.48 (CH_2_–3′); 41.29 (d, *J*_C,P_ = 5.0, CH_2_–3-Phe); 41.47 (d, *J*_C,P_ = 6.5, CH_2_–3-Phe); 53.03 (CH_2_–5′); 54.45 (CH-4′); 55.56, 55.92 (CH-2-Phe); 59.90 (CH-2′); 62.28, 62.33 (CH_3_**C**H_2_O); 63.18 (CH_2_–6′); 118.18 (C-5); 127.92, 128.00 (CH-*p*-Phe); 129.45, 129.52 (CH-*m*-Phe); 130.59, 130.85 (CH-*o*-Phe); 137.96 (CH-8); 138.48, 138.60 (C-*i*-Phe); 153.08 (C-4); 155.13 (C-2); 159.38 (C-6); 172.85 (d, *J*_C,P_ = 13.5, NCO); 174.74 (d, *J*_C,P_ = 3.8, C-1-Phe); 174.85 (d, *J*_C,P_ = 1.9, C-1-Phe).

^31^P{^1^H} NMR (202.5 MHz, CD_3_OD): 32.16.

**IR** ν_max_ (CHCl_3_) 3485 (vw), 3386 (w), 3319 (w, vbr), 3110 (w, vbr), 3089 (vw), 3065 (vw), 3034 (vw), 2965 (vs), 1733 (m), 1690 (m), 1625 (m), 1606 (w, sh), 1589 (w, sh), 1573 (w, sh), 1534 (w), 1496 (w), 1480 (w, sh), 1473 (s), 1462 (m), 1455 (m), 1448 (m), 1443 (m), 1398 (m), 1391 (m), 1370 (w), 1316 (vw), 1239 (m), 1183 (m), 1165 (w), 1098 (vw), 1072 (w), 1061 (w), 1034 (w), 1022 (w), 919 (vw), 701 (w), 554 (vw).

**HR-ESI** C_35_H_44_O_8_N_8_P (M-H)1 calcd 735.30252, found 735.30214.

##### Tetra-(l-phenylalanine ethyl ester) Prodrug of [2*S*,4*R*]-4-Guanin-9-yl-2-(2-phosphonoethoxymethyl)-1-*N*-(3-phosphonopropionyl)pyrrolidine **15**

Compound **15** was prepared according to general procedure G in 12% yield (69 mg, 58 μmol).

A mixture of two rotamers ∼ 4:1, NMR of major rotamer:

^1^H NMR (500.0 MHz, CD_3_OD): 1.18, 1.19, 1.23, 1.26 (4 × t, 4 × 3H, *J*_vic_ = 7.1, C**H**_**3**_CH_2_O); 1.51–1.75 (m, 4H, PC**H**_**2**_CH_2_CO, PC**H**_**2**_CH_2_O); 2.01 (m, 1H, H-3′b); 2.04–2.20 (m, 2H, PCH_2_C**H**_**2**_CO); 2.33 (ddd, 1H, *J*_gem_ = 12.8, *J*_3′a,4′_ = 9.8, *J*_3′a,2′_ = 9.0, H-3′a); 2.69 (dd, 1H, *J*_gem_ = 13.6, *J*_vic_ = 8.2, H-3b-Phe); 2.77 (dd, 1H, *J*_gem_ = 13.4, *J*_vic_ = 7.5, H-3b-Phe); 2.82 (dd, 1H, *J*_gem_ = 13.5, *J*_vic_ = 8.7, H-3b-Phe); 2.91 (dd, 1H, *J*_gem_ = 13.5, *J*_vic_ = 7.9, H-3b-Phe); 2.93–2.99, 3.03–3.09 (2 × m, 2 × 2H, H-3a-Phe); 3.39–3.53 (m, 4H, OCH_2_, PCH_2_C**H**_**2**_O); 3.61 (dd, 1H, *J*_gem_ = 10.5, *J*_5′b,4′_ = 7.8, H-5′b); 3.78 (dd, 1H, *J*_gem_ = 10.5, *J*_5′a,4′_ = 7.8, H-5′a); 3.86 (ddd, 1H, *J*_2,3_ = 9.0, 8.7, *J*_H,P_ = 5.7, H-2-Phe); 3.93 (ddd, 1H, *J*_2,3_ = 9.2, 7.5, *J*_H,P_ = 5.4, H-2-Phe); 4.01–4.24 (m, 11H, H-2′, H-2-Phe, CH_3_C**H**_**2**_O); 5.13 (dq, 1H, *J*_4′,3′_ = 9.0, 7.8, *J*_4′,5′_ = 7.8, H-4′); 7.09–7.32 (m, 20H, H-*o,m,p*-Ph); 7.68 (s, 1H, H-8).

^13^C NMR (125.7 MHz, CD_3_OD): 14.43, 14.46, 14.50, 14.55 (**C**H_3_CH_2_O); 24.63 (d, *J*_C,P_ = 118.2, P**C**H_2_CH_2_CO); 29.09 (d, *J*_C,P_ = 2.0, PCH_2_**C**H_2_CO); 31.11 (d, *J*_C,P_ = 115.0, P**C**H_2_CH_2_O); 33.04 (CH_2_–3′); 41.21–41.51 (m, CH_2_-3-Phe); 52.07 (CH_2_-5′); 54.35 (CH-4′); 55.28, 55.53, 55.71, 55.85 (CH-2-Phe); 57.51 (CH-2′); 62.26, 62.30, 62.35, 62.43 (CH_3_**C**H_2_O); 66.71 (d, *J*_C,P_ = 5.0, PCH_2_**C**H_2_O); 71.75 (OCH_2_); 118.15 (C-5); 127.89, 127.93, 127.99 (CH-*p*-Ph); 129.43, 129.49, 129.51 (CH-*m*-Ph); 130.61, 130.86, 130.87 (CH-*o*-Ph); 138.41, 138.49, 138.63 (C-*i*-Ph); 138.38 (CH-8); 138.41, 138.49, 138.63 (C-*i*-Ph); 153.08 (C-4); 155.08 (C-2); 159.44 (C-6); 172.72 (d, *J*_C,P_ = 13.7, NCO); 174.49 (d, *J*_C,P_ = 5.4, C-1-Phe); 174.67 (d, *J*_C,P_ = 3.9, C-1-Phe); 174.78, 174.79 (2 × d, *J*_C,P_ = 3.6, C-1-Phe).

^31^P{^1^H} NMR (202.4 MHz, CD_3_OD): 30.88, 32.07.

**IR** ν_max_ (CHCl_3_) 3386 (w), 3340 (w), 3302 (w), 3235 (w, br), 3202 (v, br), 3111 (vw), 3088 (vw), 3066 (vw), 3031 (w), 2985 (m), 2929 (w), 2909 (w), 2875 (w), 1737 (vs), 1693 (s), 1635 (s), 1603 (m), 1595 (m, sh), 1571 (m), 1536 (w), 1496 (w), 1485 (w), 1476 (w), 1455 (w), 1444 (m), 1428 (m), 1394 (w), 1369 (m), 1341 (w), 1236 (m), 1198 (s, sh), 1180 (s), 1158 (m), 1116 (m), 1098 (m, sh), 1079 (w), 1030 (m), 990 (w), 966 (w), 910 (w), 857 (w), 702 (m), 508 (vw).

**HR-ESI** C_59_H_77_O_13_N_10_P_2_ (M+H)^+^ calcd 1195.51413, found 1195.51577.

### Determination of *K*_i_ Values

All the H/G/XPRTs were expressed and purified to homogeneity as previously described.^31,34,41−43,45^ The *K*_m_ for PRPP and the *K*_i_ values for the compounds were measured in a continuous spectrophotometric assay in 0.1 M Tris–HCl, 0.01 M MgCl_2_, pH 7.4, 25 °C. The substrates and inhibitor were added to the cuvette, the reaction initiated by the addition of enzyme and followed for 60 s at 257.5 nm. The concentration of guanine was fixed at 60 μM, and the concentration of PRPP varied between 5 and 1000 μM depending on the *K*_m(app)_ in the presence of the inhibitor. The Δε for the reaction is 5816.5 M^–1^ cm^–1^. GraphPad Prism was used to calculate the *K*_i_ value using the equation for competitive inhibitors, *K*_m(app)_ = *K*_m_(1+[*I*]_o_/*K*_i_).

### Crystallization and Structure Determination

The concentration of human enzyme incubated with the ligand was 10 mg mL^–1^ (0.4 mM), and the concentration of *Tbr*HGPRT1 was 8 mg mL^–1^ (0.3 mM). The concentration of the inhibitors was between 5 and 6 mM. The crystallization conditions are as follows: Human HGPRT (**1**): 0.3 M calcium acetate, 20% PEG 3350; human HGPRT (**5**): 0.3 M sodium acetate, 17.5% PEG 3350; *TBr*HGPRT1 with **4** or **5**: 0.2 M MgCl_2_, 0.1 M Bis–Tris, pH 5.0, 20% 3350. The crystals were obtained using the hanging drop method; for the human enzyme, 1 μL of enzyme and 1 μL of well solution formed the hanging drop. For *Tbr*HGPRT1, 2 μL of enzyme and 2 μL of well solution were used to form the hanging drop. The crystals were cryocooled in liquid nitrogen for transport to the Australian Synchrotron where they were robotically placed in the cryostream (100 K) of beamline MX1. X-ray data were collected remotely.^58^ All data were scaled and merged with XDS. The structure was solved by molecular replacement using the program PHASER within PHENIX 1.18.2. For human HGPRT, the starting model was PDB code: 5HIA, for *Tbr*HGPRT1, PDB code: 5JV5, and for *Tbr*HGPRT1, PDB code: 6AQO. Subsequent refinement and model building was with PHENIX 1.18.2^59^ and COOT 0.8.9.2.^60^ The pdb file of the ligand was generated using ELBOW in PHENIX.^59^

### *T. brucei* Drug Sensitivity Assay

Drug sensitivity assays using the resazurin sodium salt dye (Alamar Blue Assay) were performed according to the published protocol^61^ in a 96-well plate format. Tests were performed on bloodstream trypomastigotes *T. brucei brucei* strain 427 in HMI-11 with 10% fetal bovine serum. Bloodstream from parasites at 5 × 10^3^ per well was incubated with different drug concentrations (twofold serial dilutions) in a volume of 200 μL of the HMI-11 medium. The plates were left for 48 h at 37 °C and 5% CO_2_. Then, 20 μL of resazurin sodium salt solution (0.125 mg/mL in 1× PBS, pH 7. 4) was added to each well and cells were incubated for additional 24 h. The fluorescence signal was quantified using a Tecan Infinite M200 plate reader at excitation and emission wavelengths of 560 and 590 nm, respectively. The effective concentration 50% (EC_50_) values were calculated using nonlinear regression with a variable slope by GraphPad Prism 8.0 (GraphPad Prism Software, San Diego, California, USA).

## Supporting Information Available

NMR spectra and LC-MS traces for final compounds (PDF)
Molecular formula strings (CSV)

## Accession Codes

The coordinates and structure factors of all four complexes have been deposited in the Protein Data Bank with access codes 8TS4 for the *Tbr*HGPRT1·**4** complex, 8TPY for human HGPRT·**1** complex, 8TR1 for the *Tbr*HGPRT1·**5** complex, and 8TPV for the human HGPRT·**5** complex. The authors will release the atomic coordinates upon article publication.

## Abbreviations

AIP: acyclic nucleoside phosphonate
ANP: acyclic nucleoside phosphonate
*Ec*: *Escherichia coli*
HGXPRT: hypoxanthine–guanine–xanthine phosphoribosyltransferase
HG(X)PRT: denotes that the enzyme uses hypoxanthine and guanine as a substrate but as a collective may or may not use xanthine as a substrate
H/G/X/PRT: PRTs that could any one, two or all three 6-oxopurine bases as a substrate
*Hp*: *Helicobacter pylori*
ImmGP: (1*S*)-1-(9-deazaguanin-9-yl)-1,4-dideoxy-1,4-imino-d-ribitol 5-phosphate
ImmHP: (1*S*)-1-(9-deazahypoxanthin-9-yl)-1,4-dideoxy-1,4-imino-d-ribitol 5-phosphate
*Mtb*: *Mycobacterium tuberculosis*
*Pf*: *Plasmodium falciparum*
PRPP: 5-phospho-α-d-ribosyl-1-pyrophosphate
PRTase: phosphoribosyltransferase
*Pv*: *Plasmodium vivax*
*Tbr*: *Trypanosoma brucei*
