# Supplementary material for: Development of Prolinol Containing Inhibitors of Hypoxanthine–Guanine–Xanthine Phosphoribosyltransferase: Rational Structure-Based Drug Design
Source: J Med Chem. 2024 Apr 23;67(9):7158–75. doi: 10.1021/acs.jmedchem.4c00021 (PMC11089518; doi:10.1021/acs.jmedchem.4c00021)

Supporting information

Development of Prolinol containing Inhibitors of Hypoxanthine-Guanine-Xanthine Phosphoribosyltransferase: Rational Structure-Based Drug Design

Dianne T. Keough,[a] Magdalena Petrová,[b] Gordon King,[c] Michal Kratochvíl,[b,d] Radek Pohl,[b] Eva Doleželová[e], Alena Zíková[e], Luke W. Guddat[a*] and Dominik Rejman[b*]

[a]*School of Chemistry and Molecular Biosciences, The University of Queensland, Brisbane, 4072 QLD, Australia*

[b]*Institute of Organic Chemistry and Biochemistry, Czech Academy of Sciences, Flemingovo nam. 2, CZ-166 10 Prague 6, Czech Republic*

[c]*The Centre for Microscopy and Microanalysis, The University of Queensland, Brisbane 4072, Australia*

[d]*University of Chemical Technology Prague, Technická 5, CZ-166 28 Prague 6, Czech Republic*

[e]*Institute of Parasitology, Biology Centre of the Czech Academy of Sciences, Branišovská 31, CZ-37005, České Budějovice, Czech Republic.*

Address for correspondence:

luke.guddat@uq.edu.au

dominik.rejman@uochb.cas.cz

Contents:

NMR spectra for final compounds

LC-MS traces for final compounds

[2*S*,4*R*] 4-Guanin-9-yl-2-hydroxymethyl-1-*N*-(3-phosphonopropionyl)pyrrolidine (1)

^{1}H NMR

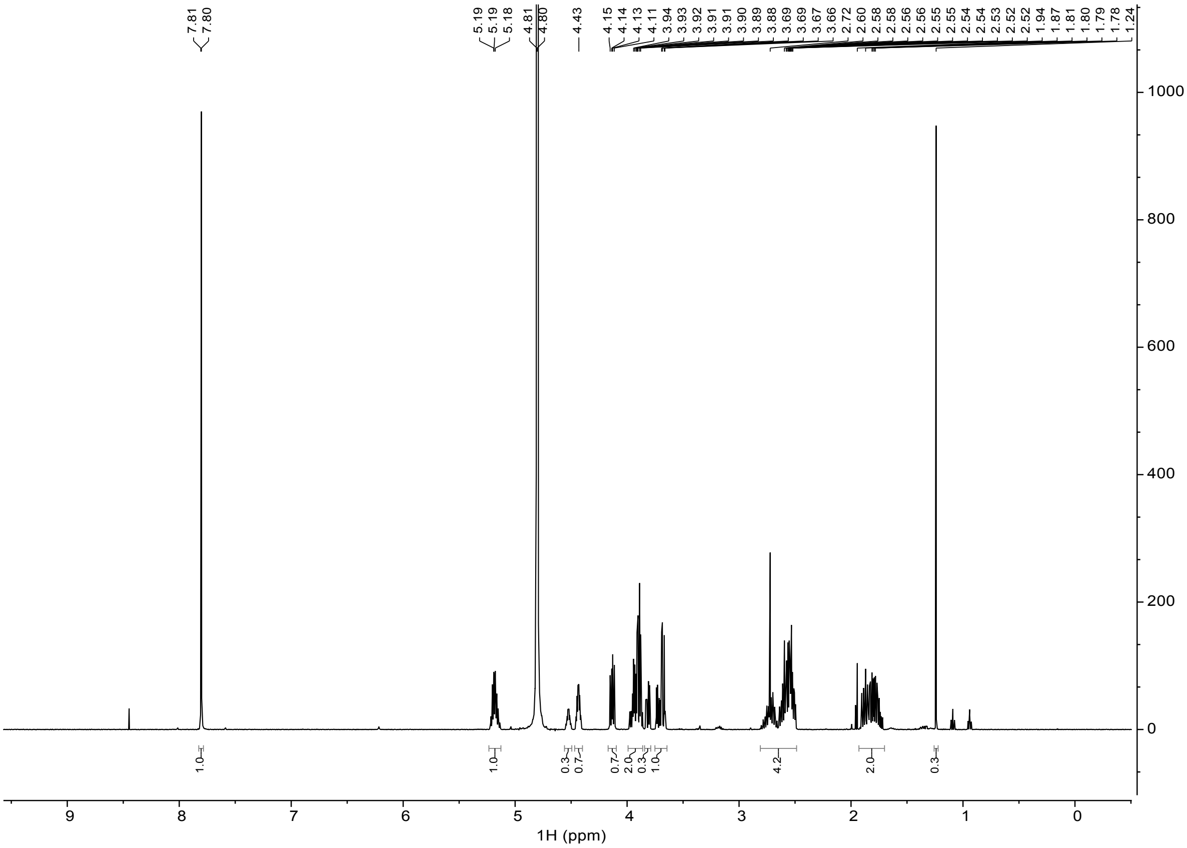

^{13}C NMR

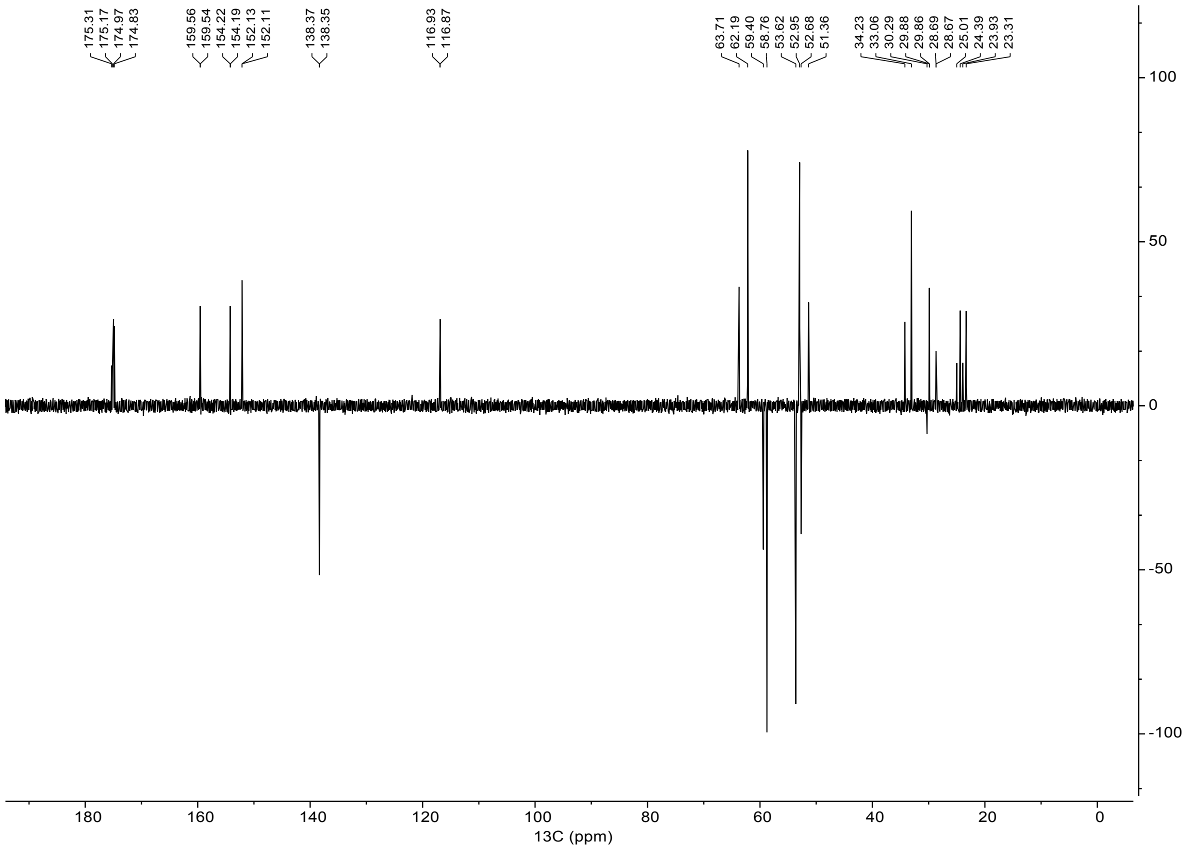

$^{31}P\{^1H\}$ NMR

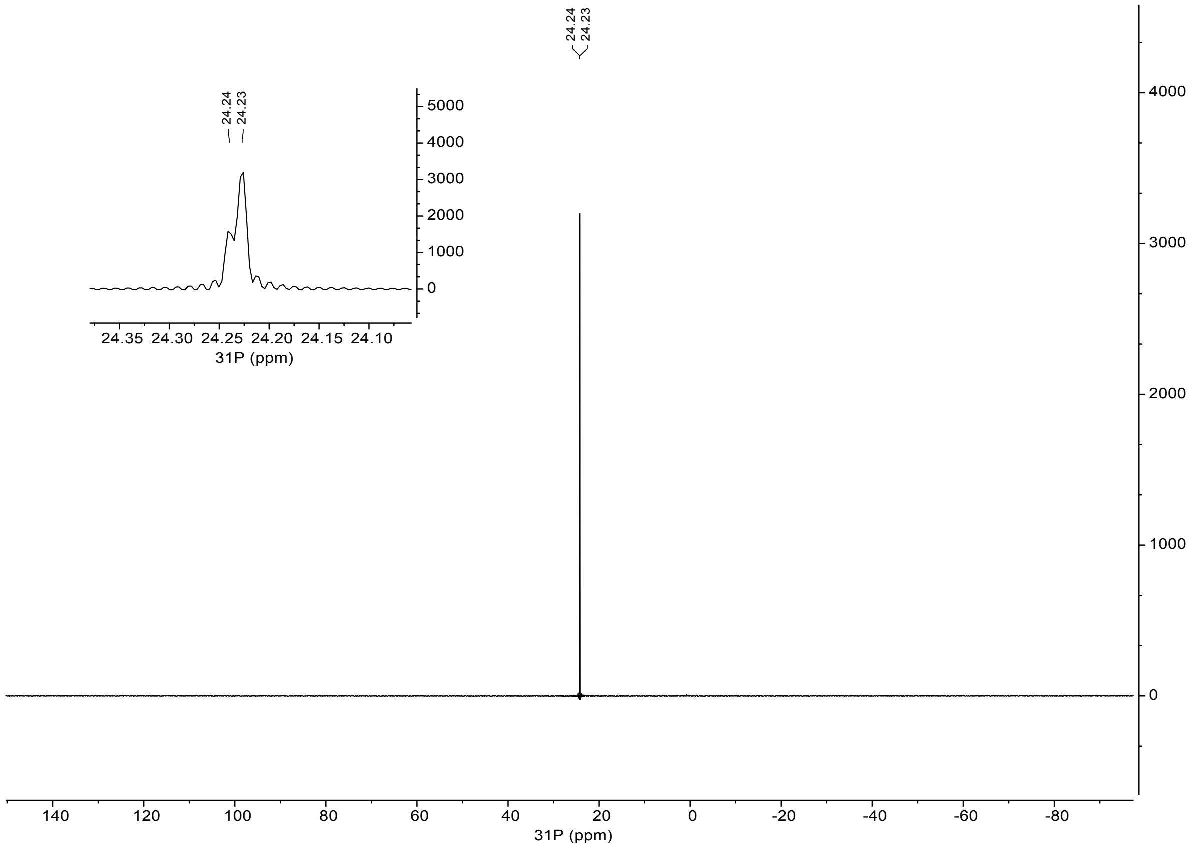

[2*R*,4*S*]-4-Guanin-9-yl-2-(2-phosphonoethoxymethyl)-1-*N*-(3-phosphonopropionyl)pyrrolidine (2)

^{1}H NMR

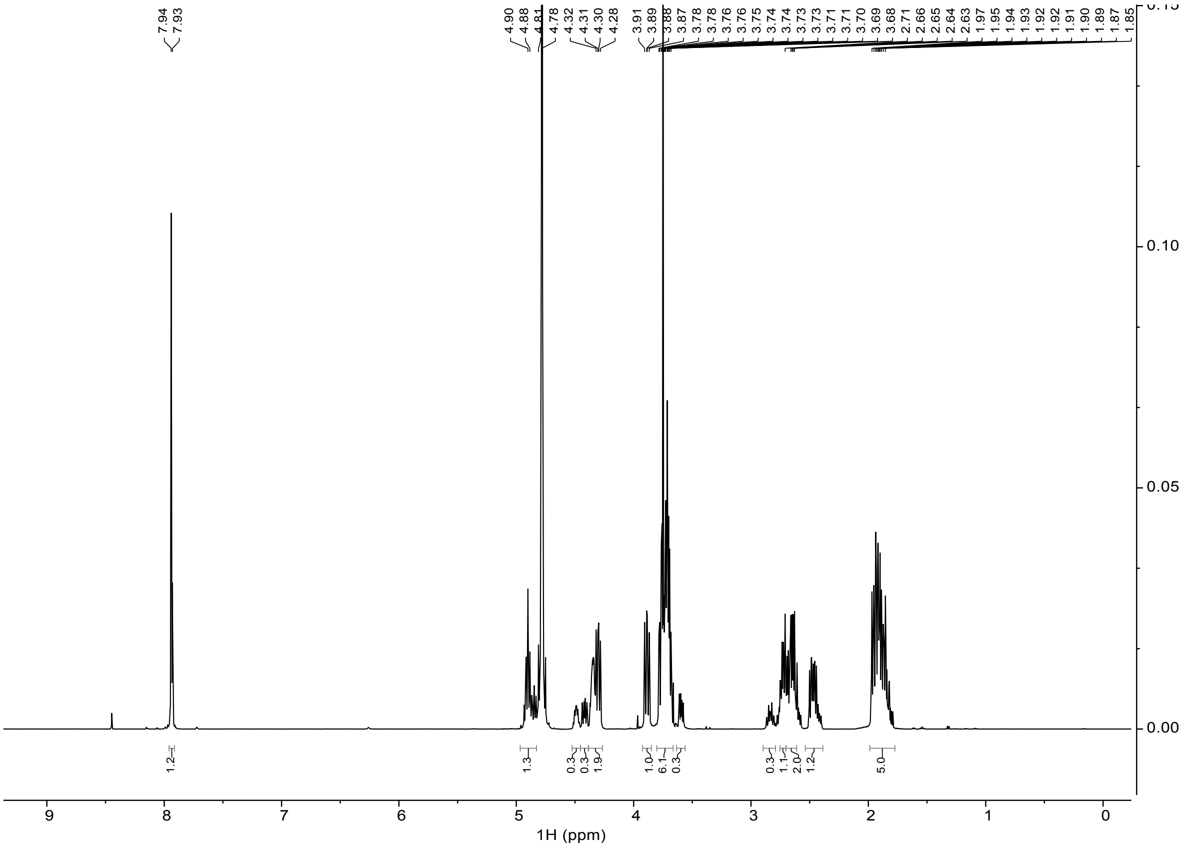

^{13}C NMR

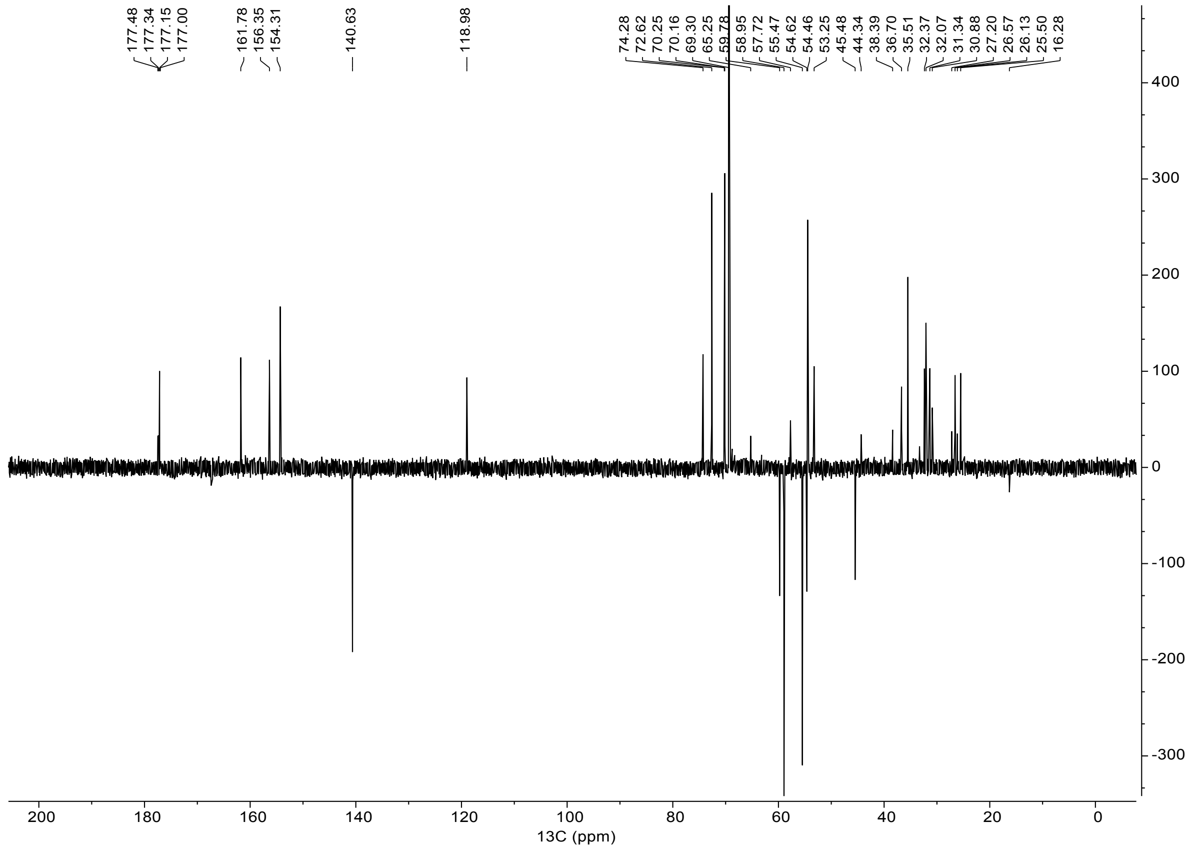

^{31}P{^{1}H} NMR

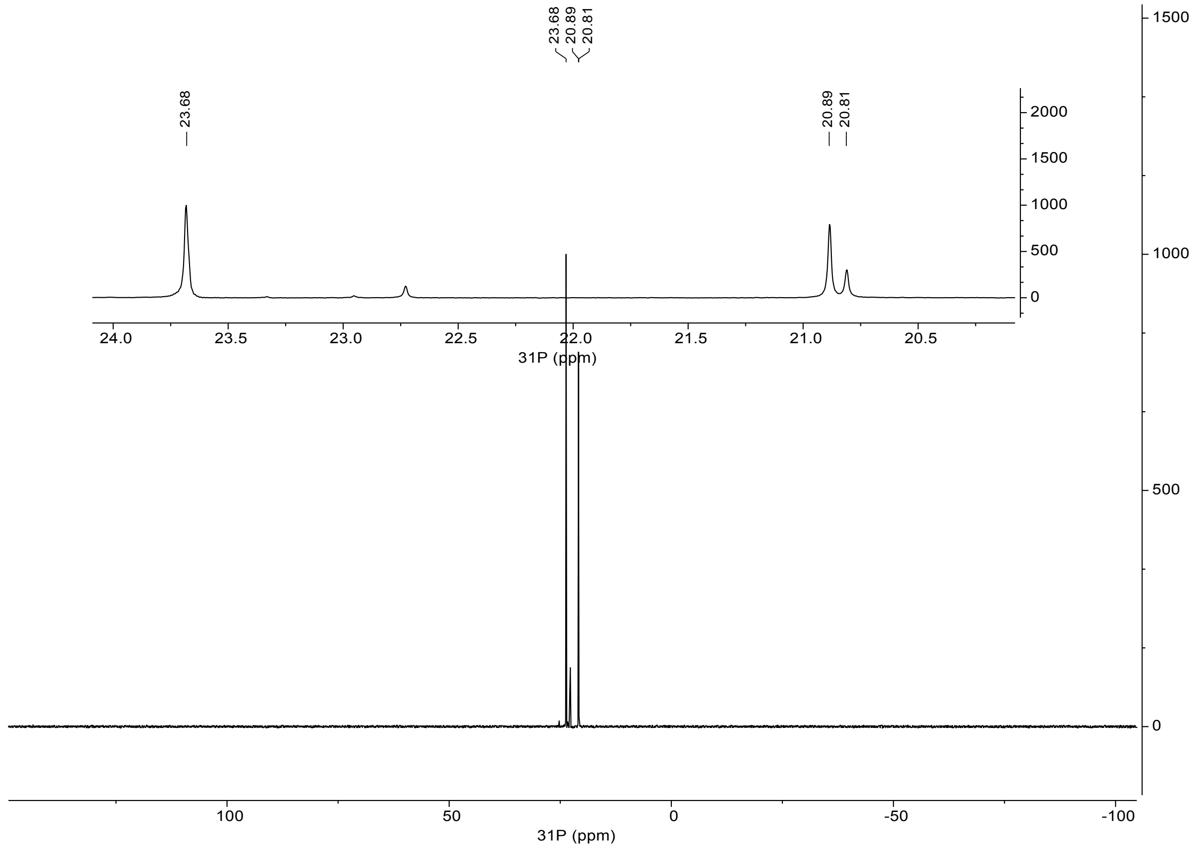

[2*R*,4*R*]-4-Guanin-9-yl-2-(2-phosphonoethoxymethyl)-1-*N*-(3-phosphonopropionyl)pyrrolidine (3)

^{1}H NMR

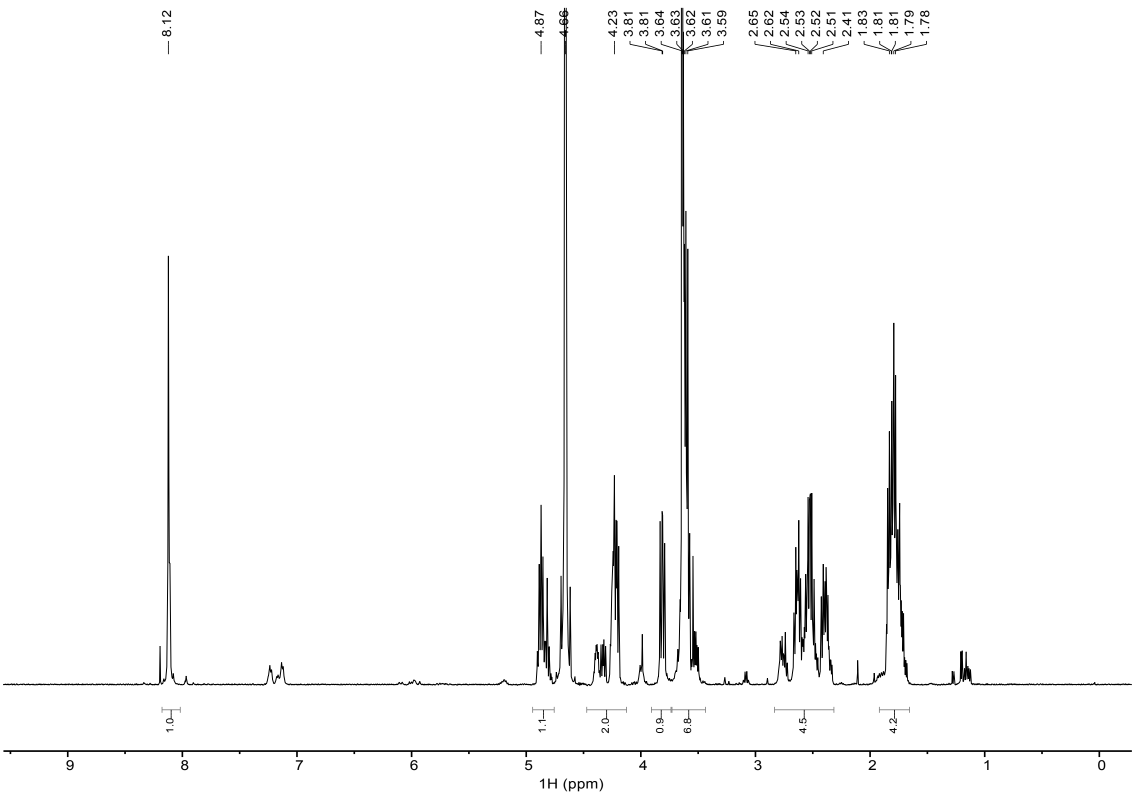

$^{31}P\{^{1}H\}$ NMR

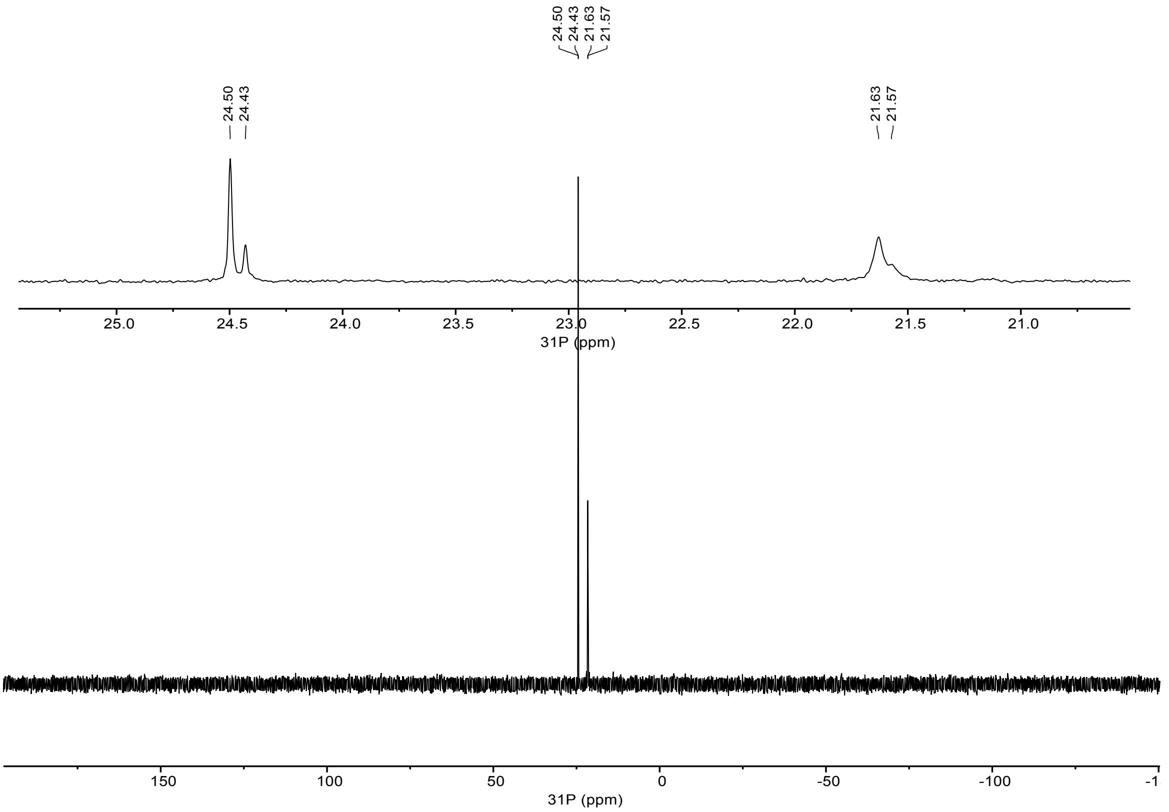
24.50
24.43
21.63
21.57
25.0
24.5
24.0
23.5
23.0
22.5
22.0
21.5
21.0
31P (ppm)
150
100
50
0
-50
-100
31P (ppm)

[2*S*,4*S*]-4-Guanin-9-yl-2-(2-phosphonoethoxymethyl)-1-*N*-(3-phosphonopropionyl)pyrrolidine (4)

^{1}H NMR

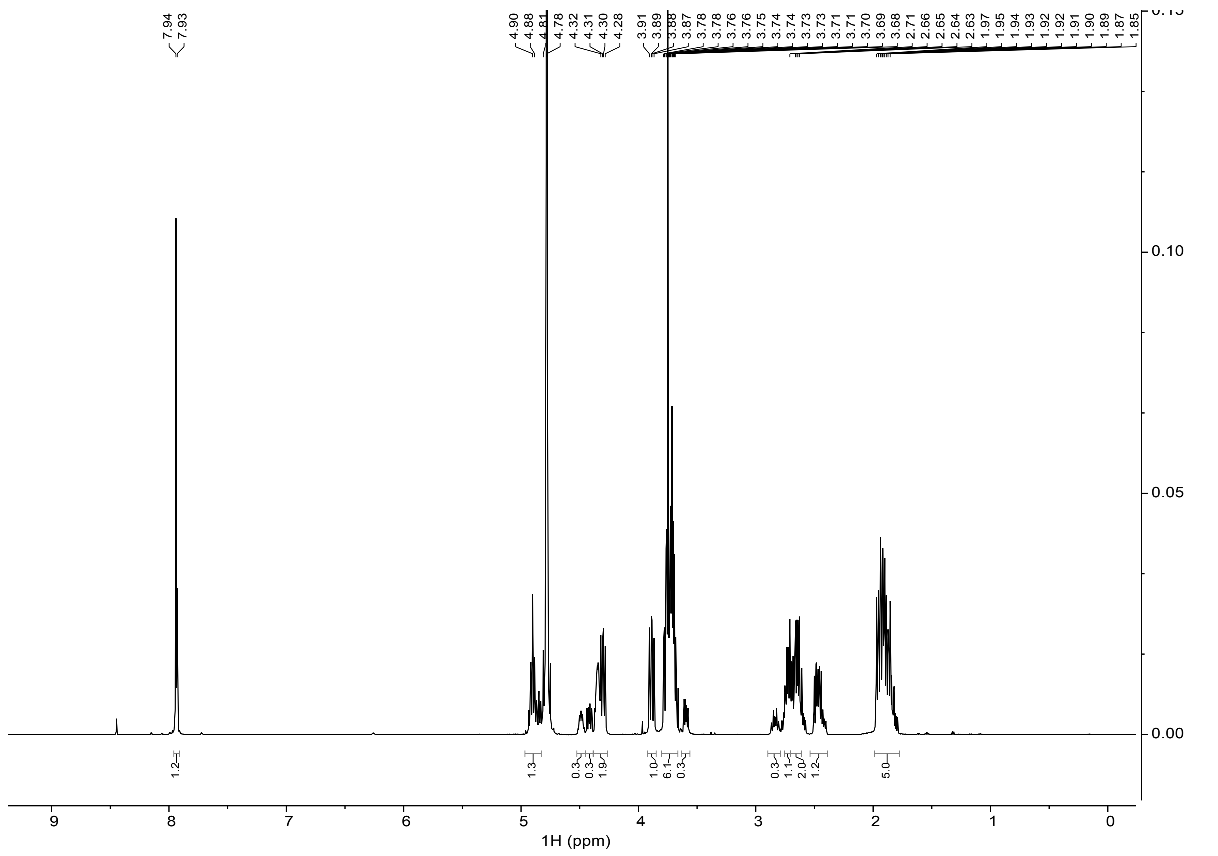

^{13}C NMR

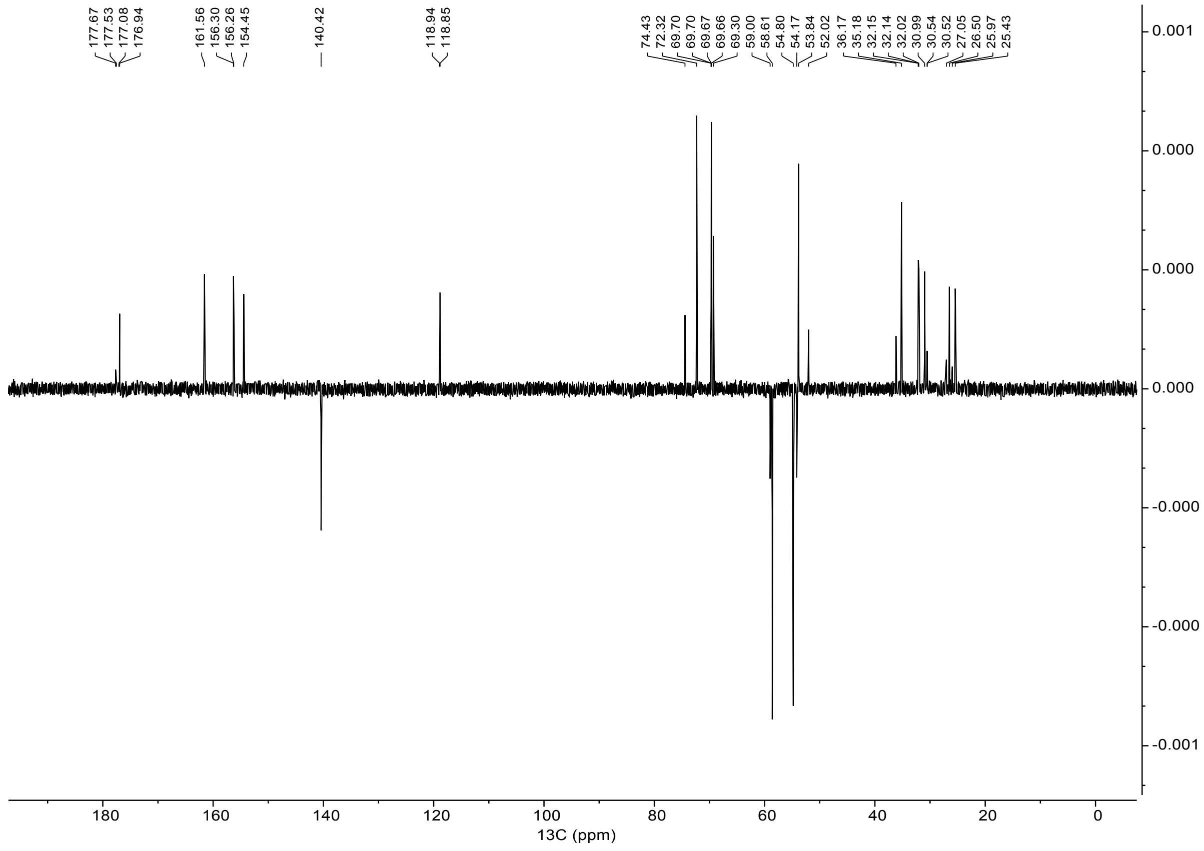

$^{31}P\{^{1}H\}$ NMR

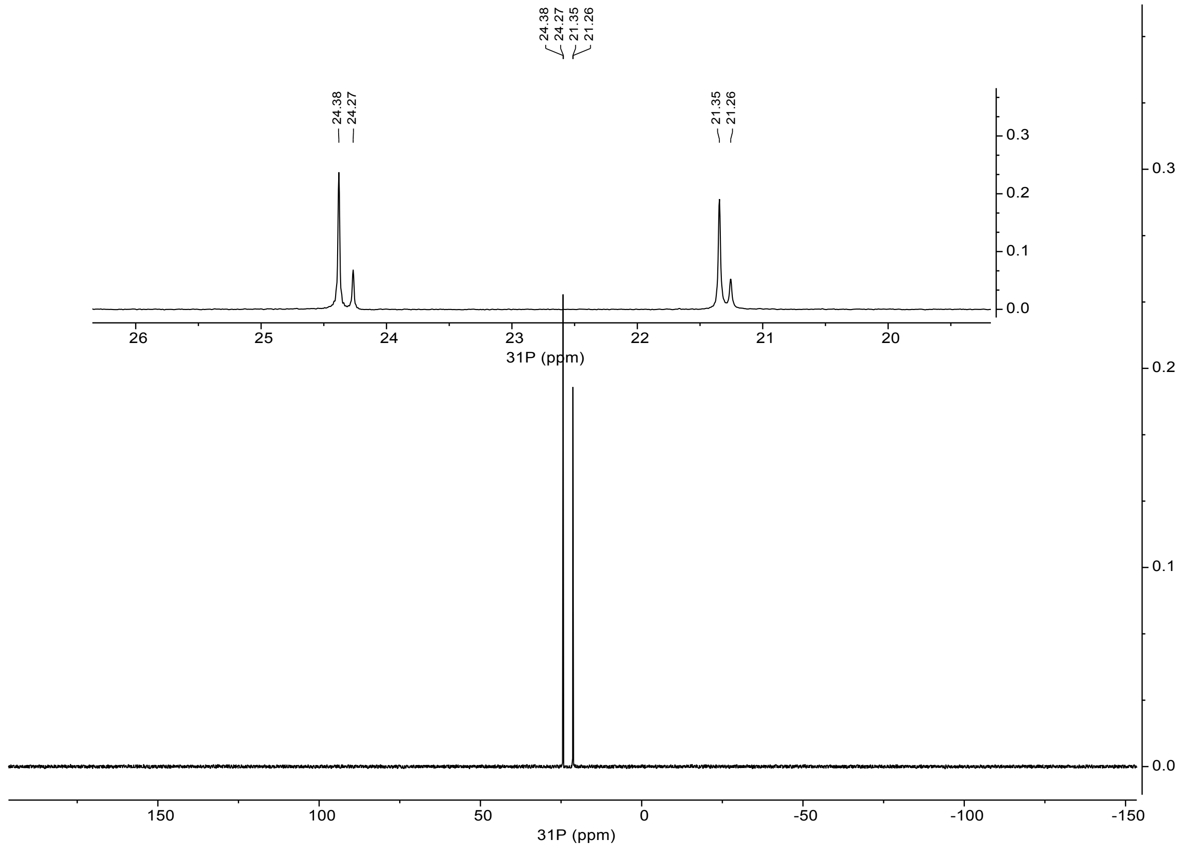

[2*S*,4*R*]-4-Guanin-9-yl-2-(2-phosphonoethoxymethyl)-1-*N*-(3-phosphonopropionyl)pyrrolidine (5)

^{1}H NMR

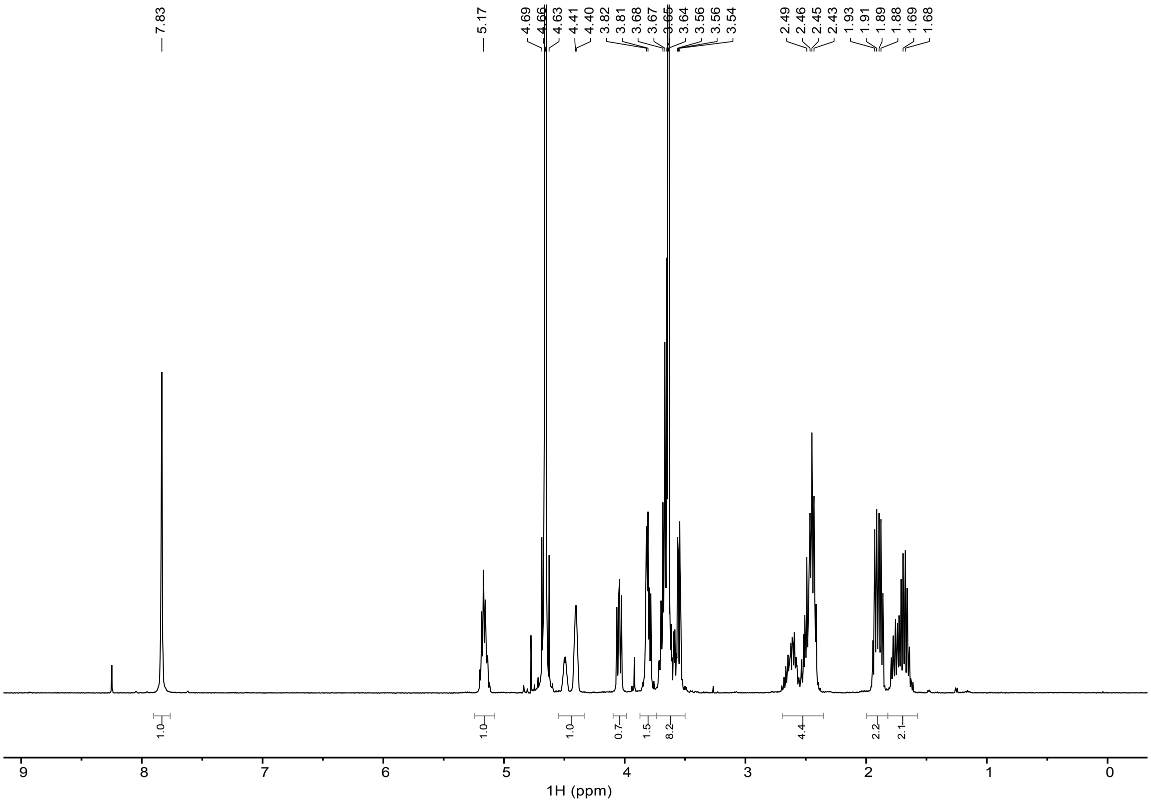

^{31}P{^{1}H} NMR

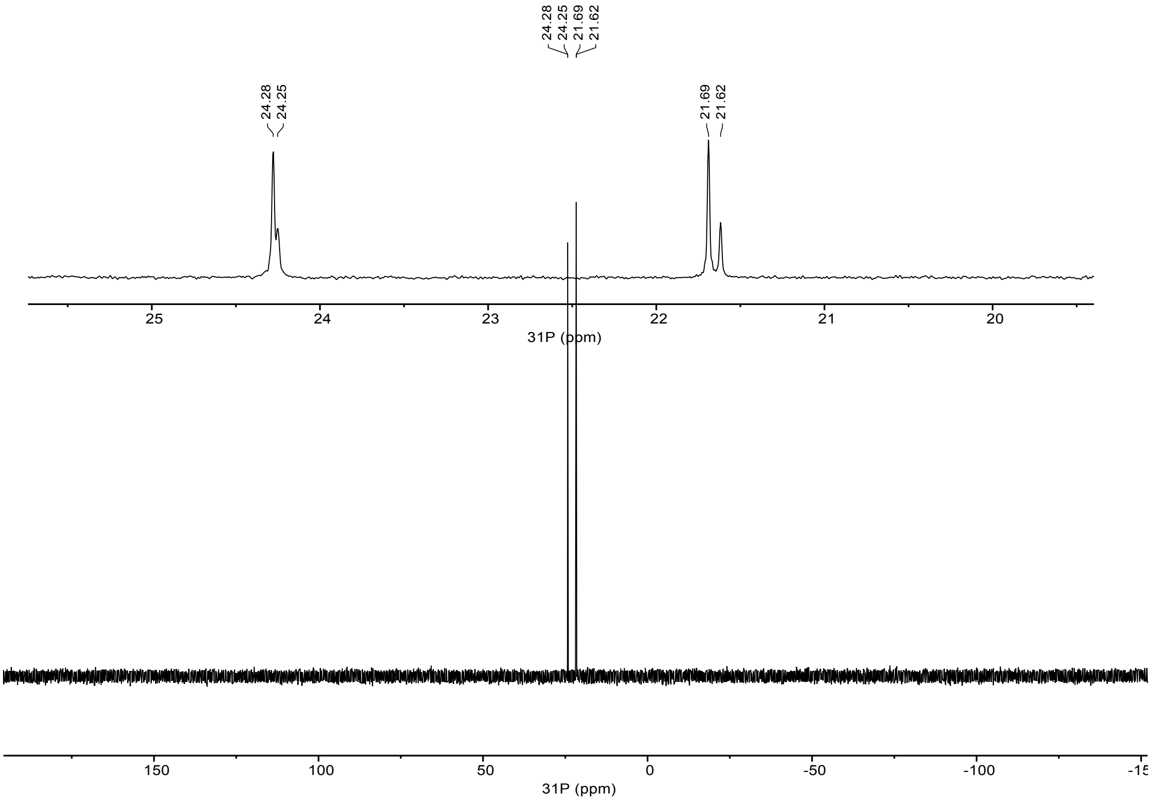

Bis-(L-phenylalanine ethyl ester) prodrug of [2*S*,4*R*] 4-Guanin-9-yl-2-hydroxymethyl-1-*N*-(3-phosphonopropionyl)pyrrolidine 14

^{1}H NMR

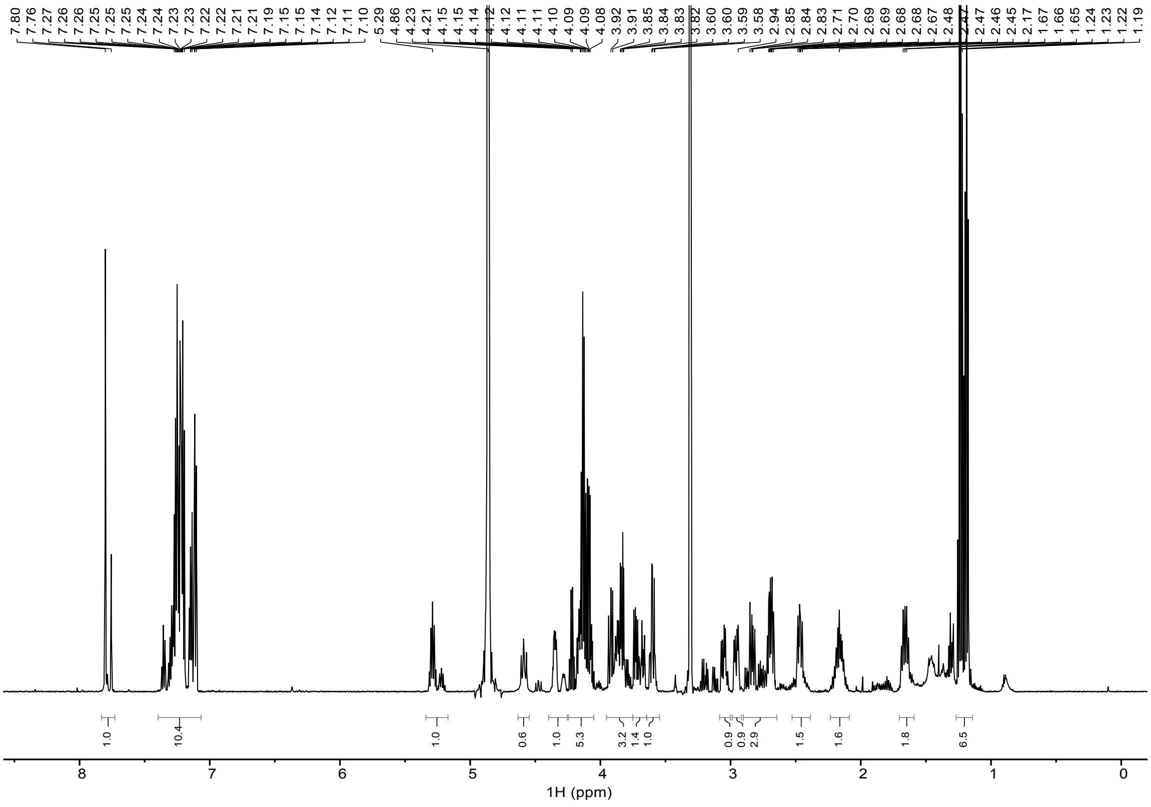

^{13}C NMR

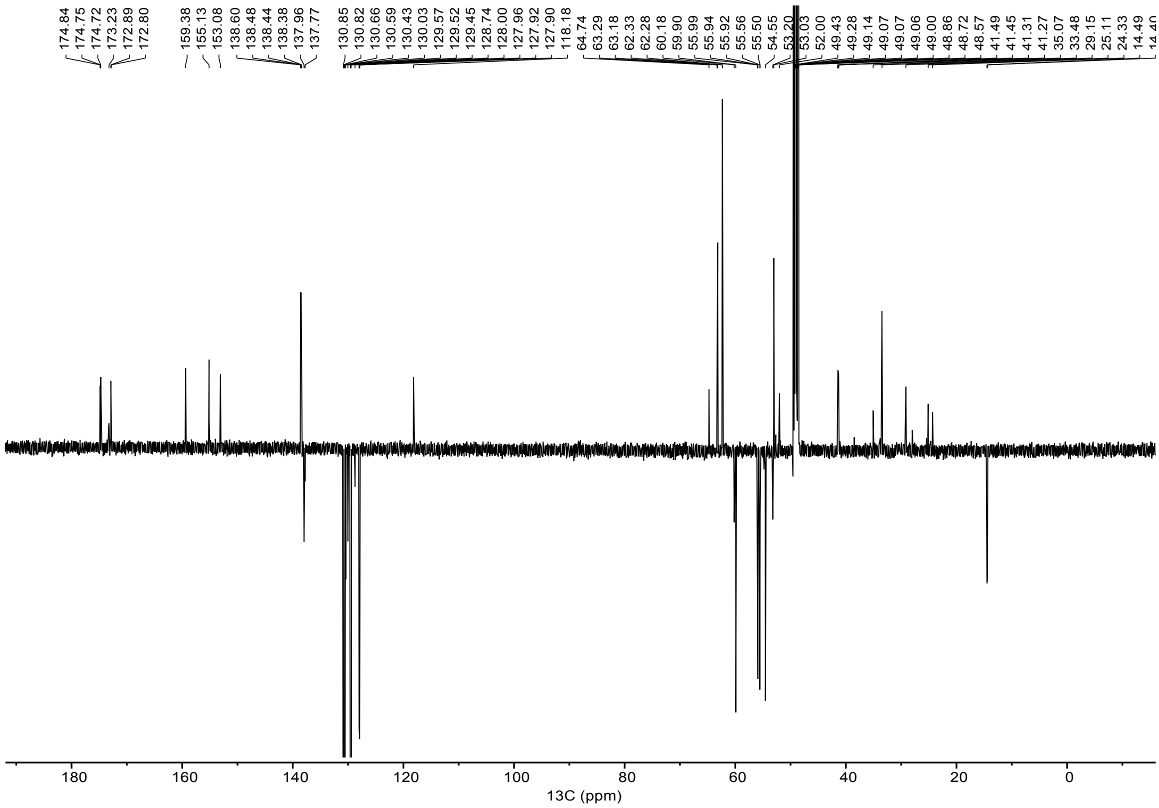
174.84
174.75
174.72
173.23
172.89
172.80
159.38
155.13
153.08
138.60
138.48
138.44
138.38
137.96
137.77
130.85
130.82
130.66
130.59
130.43
130.03
129.57
129.52
129.45
128.74
128.00
127.96
127.92
127.90
118.18
64.74
63.29
63.18
62.33
62.28
60.18
59.90
55.99
55.94
55.92
55.56
55.50
54.55
53.20
53.03
52.00
49.43
49.28
49.14
49.07
49.07
49.06
49.00
48.86
48.72
48.57
41.49
41.45
41.31
41.27
35.07
33.48
29.15
25.11
24.33
14.49
180
160
140
120
100
80
60
40
20
0
13C (ppm)

$^{31}P\{^{1}H\}$ NMR

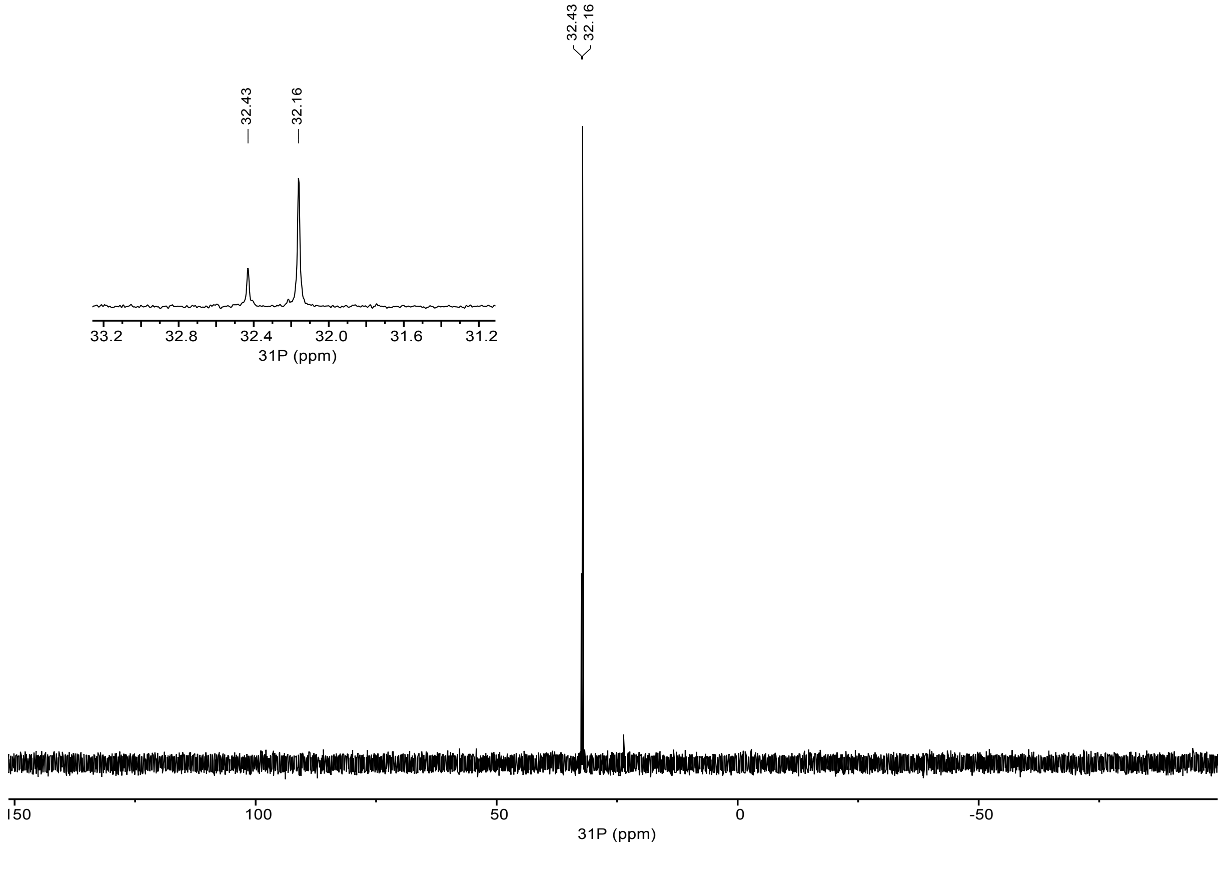
32.43
32.16
32.43
32.16
33.2
32.8
32.4
32.0
31.6
31.2
31P (ppm)
150
100
50
0
-50
31P (ppm)

Tetra-(L-phenylalanine ethyl ester) prodrug of [2*S*,4*R*]-4-Guanin-9-yl-2-(2-phosphonoethoxymethyl)-1-*N*-(3-phosphonopropionyl)pyrrolidine 15

^{1}H NMR

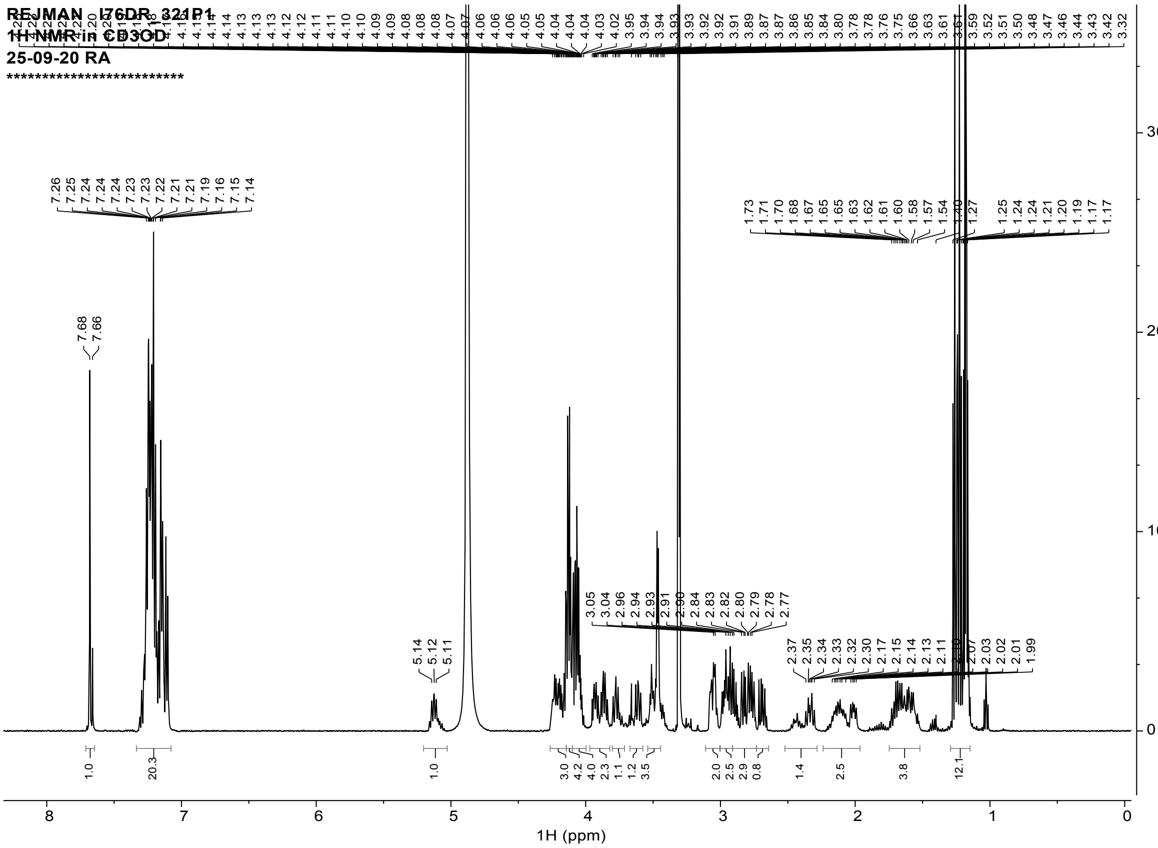

^{13}C NMR

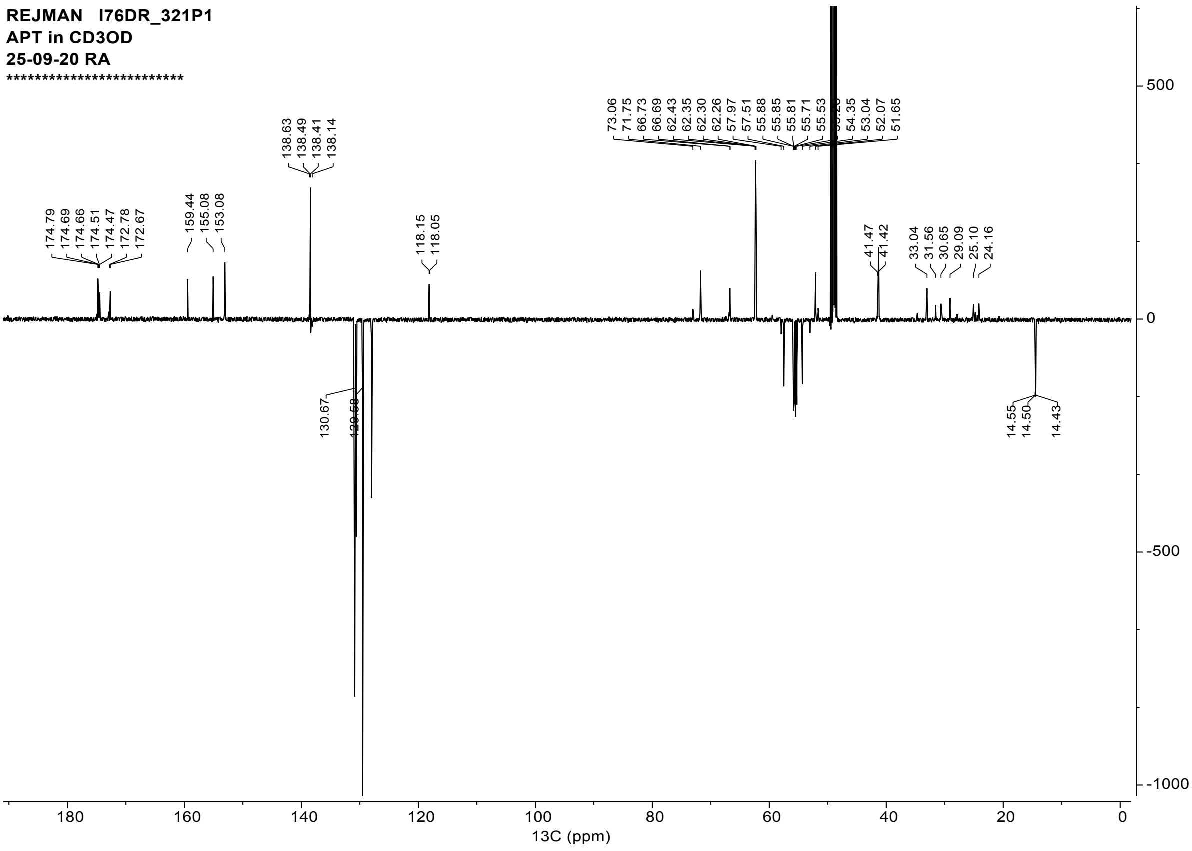
REJMAN I76DR_321P1
APT in CD3OD
25-09-20 RA
****
174.79
174.69
174.66
174.51
174.47
172.78
172.67
159.44
155.08
153.08
138.63
138.49
138.41
138.14
118.15
118.05
130.67
73.06
71.75
66.73
66.69
62.43
62.35
62.30
62.26
57.97
57.51
55.88
55.85
55.81
55.71
55.53
54.35
53.04
52.07
51.65
41.47
41.42
33.04
31.56
30.65
29.09
25.10
24.16
14.55
14.50
14.43
500
0
-500
-1000
180
160
140
120
100
80
60
40
20
0
13C (ppm)

$^{31}P\{^1H\}$ NMR

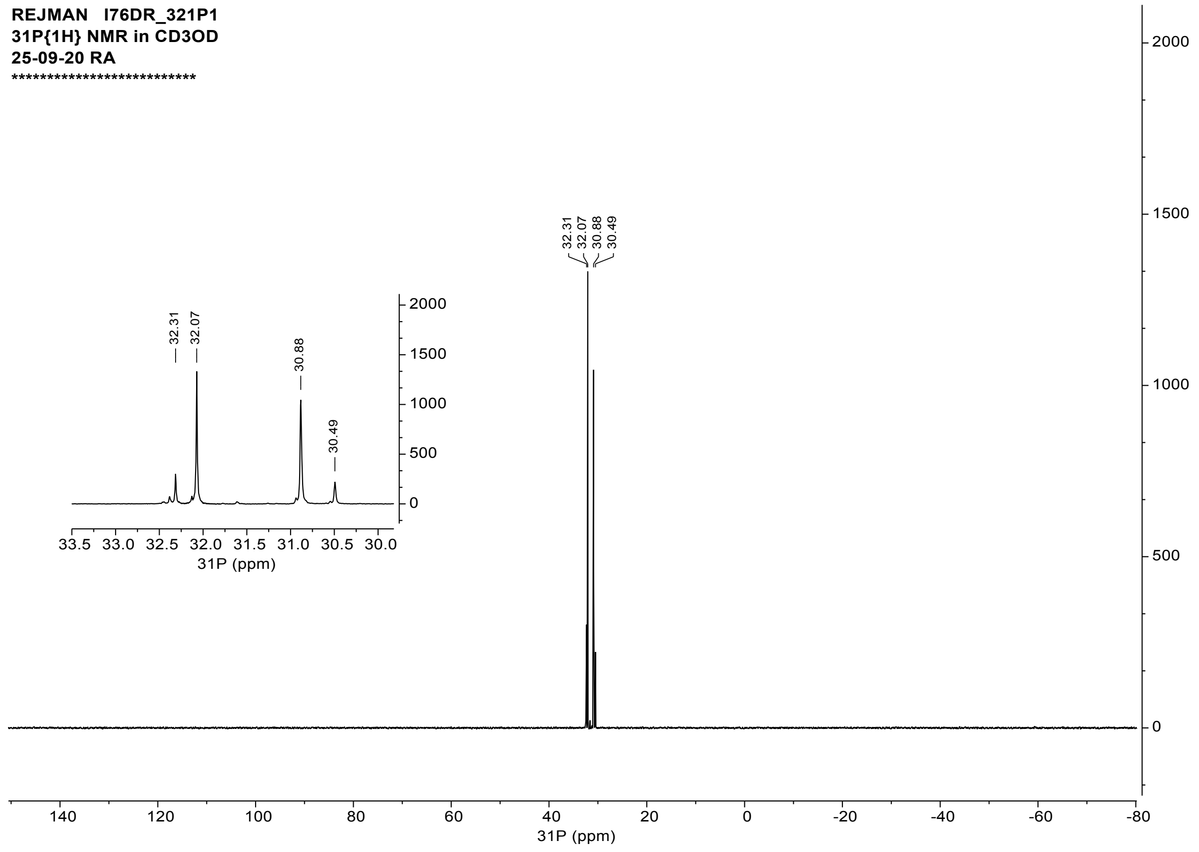
REJMAN I76DR_321P1
31P{1H} NMR in CD3OD
25-09-20 RA
****
32.31
32.07
30.88
30.49
2000
1500
1000
500
0
33.5 33.0 32.5 32.0 31.5 31.0 30.5 30.0
31P (ppm)
140 120 100 80 60 40 20 0 -20 -40 -60 -80
31P (ppm)

LC-MS traces for final compounds

[2*S*,4*R*] 4-Guanin-9-yl-2-hydroxymethyl-1-*N*-(3-phosphonopropionyl)pyrrolidine (1)

Purity >98%

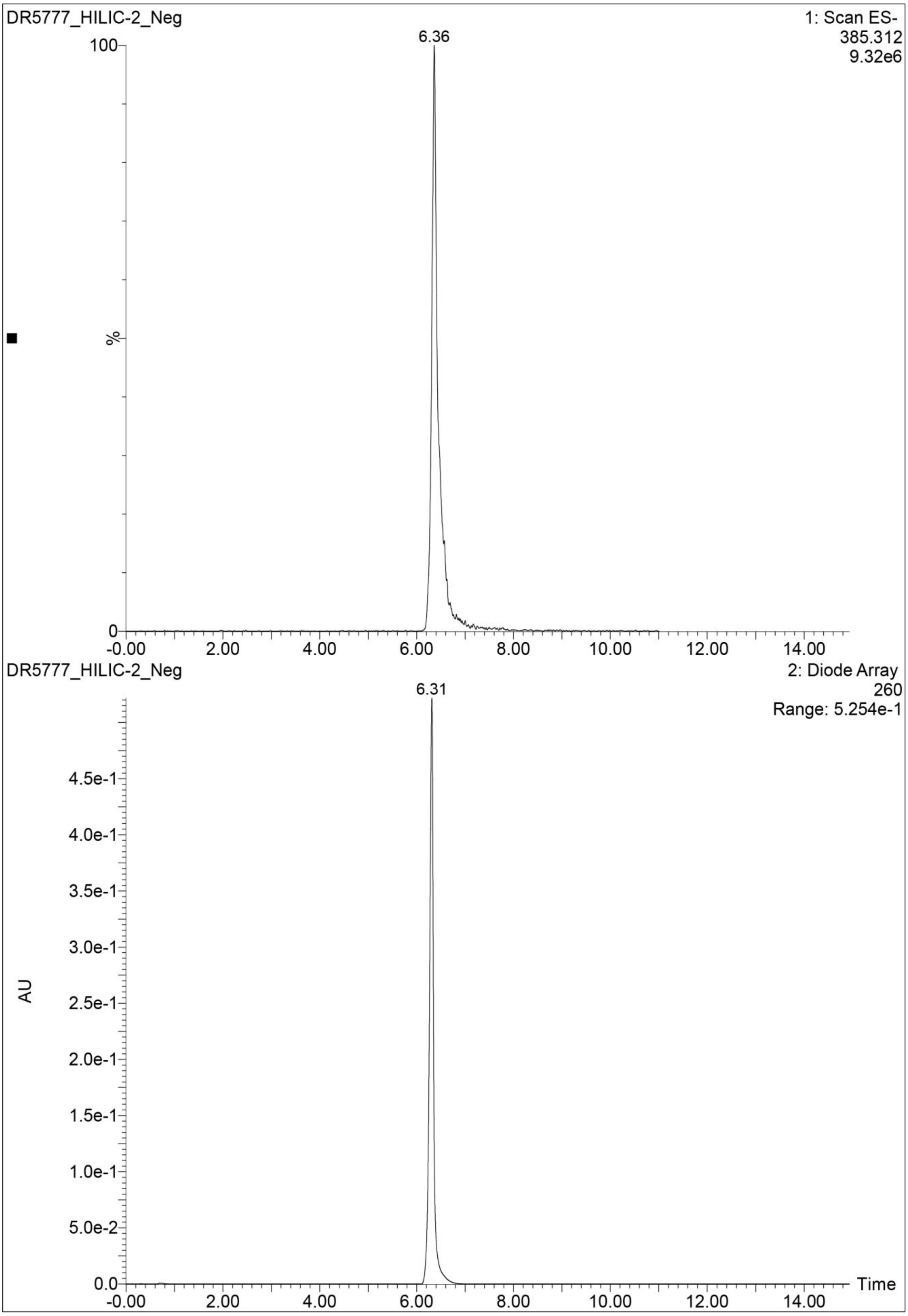

[2*R*,4*S*]-4-Guanin-9-yl-2-(2-phosphonoethoxymethyl)-1-*N*-(3-phosphonopropionyl)pyrrolidine (2) Purity >98%

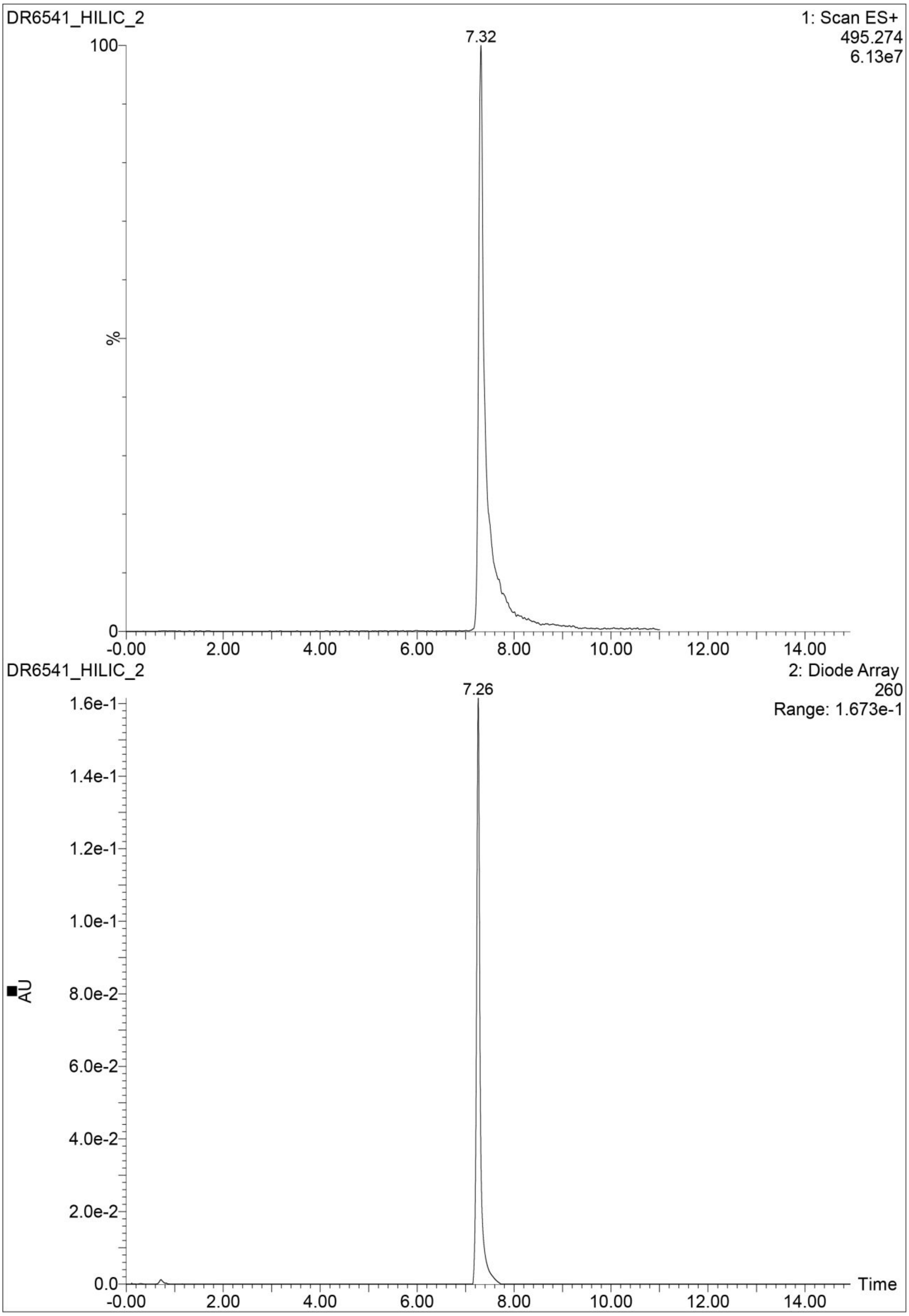

[2*R*,4*R*]-4-Guanin-9-yl-2-(2-phosphonoethoxymethyl)-1-*N*-(3-phosphonopropionyl)pyrrolidine (3) Purity >98%

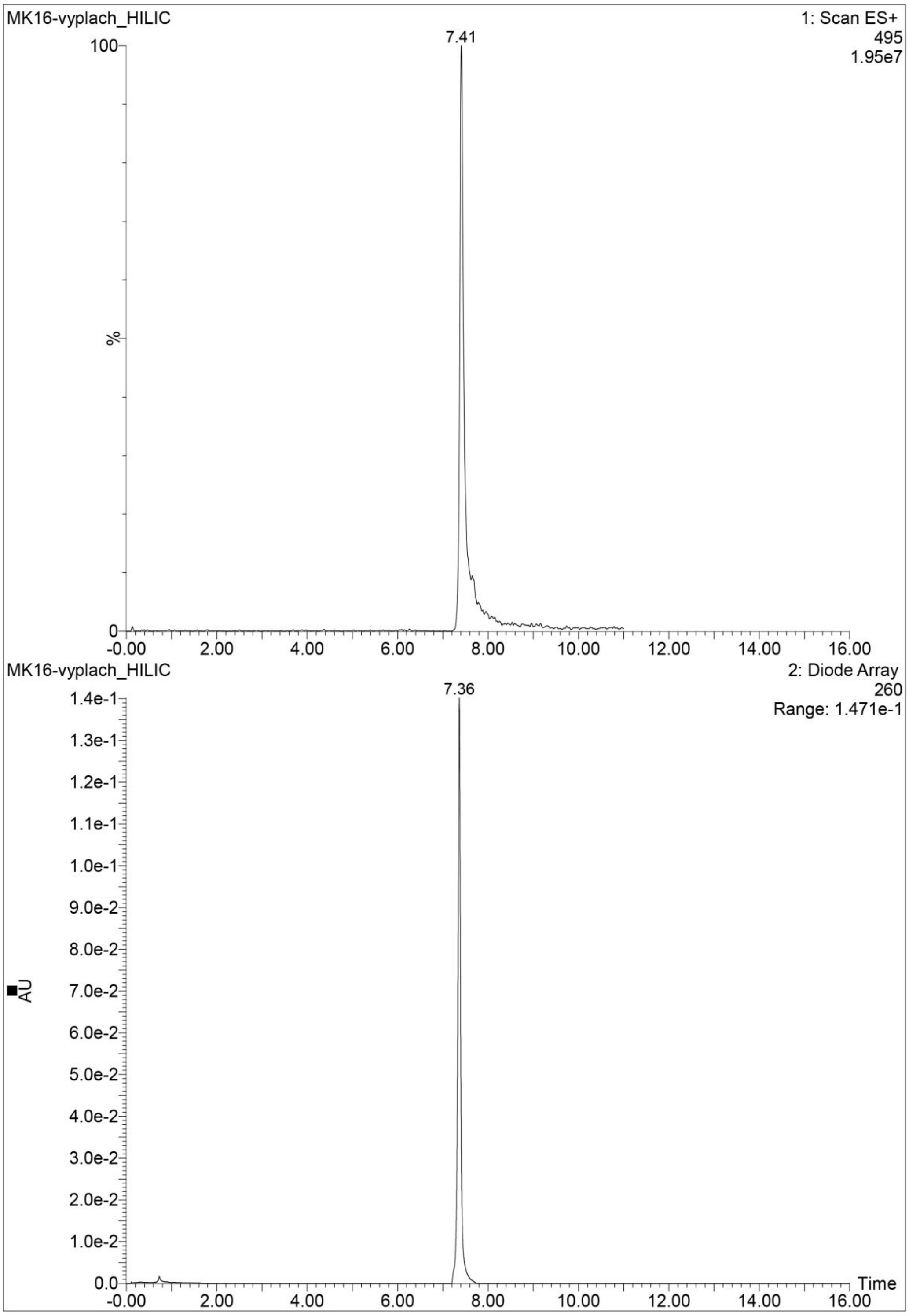

[2*S*,4*S*]-4-Guanin-9-yl-2-(2-phosphonoethoxymethyl)-1-*N*-(3-phosphonopropionyl)pyrrolidine (4) Purity >98%

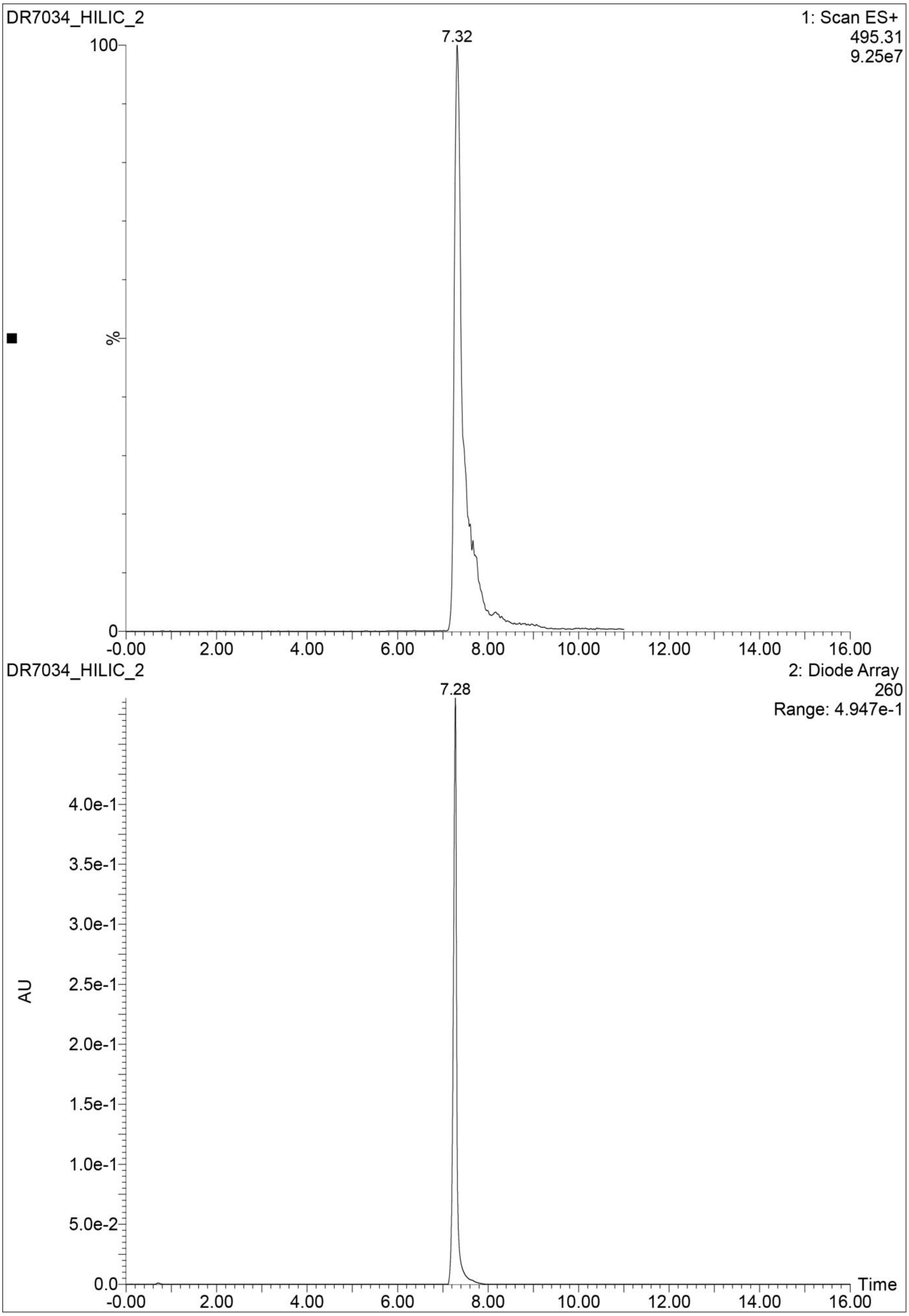

[2*S*,4*R*]-4-Guanin-9-yl-2-(2-phosphonoethoxymethyl)-1-*N*-(3-phosphonopropionyl)pyrrolidine (5) Purity >98%

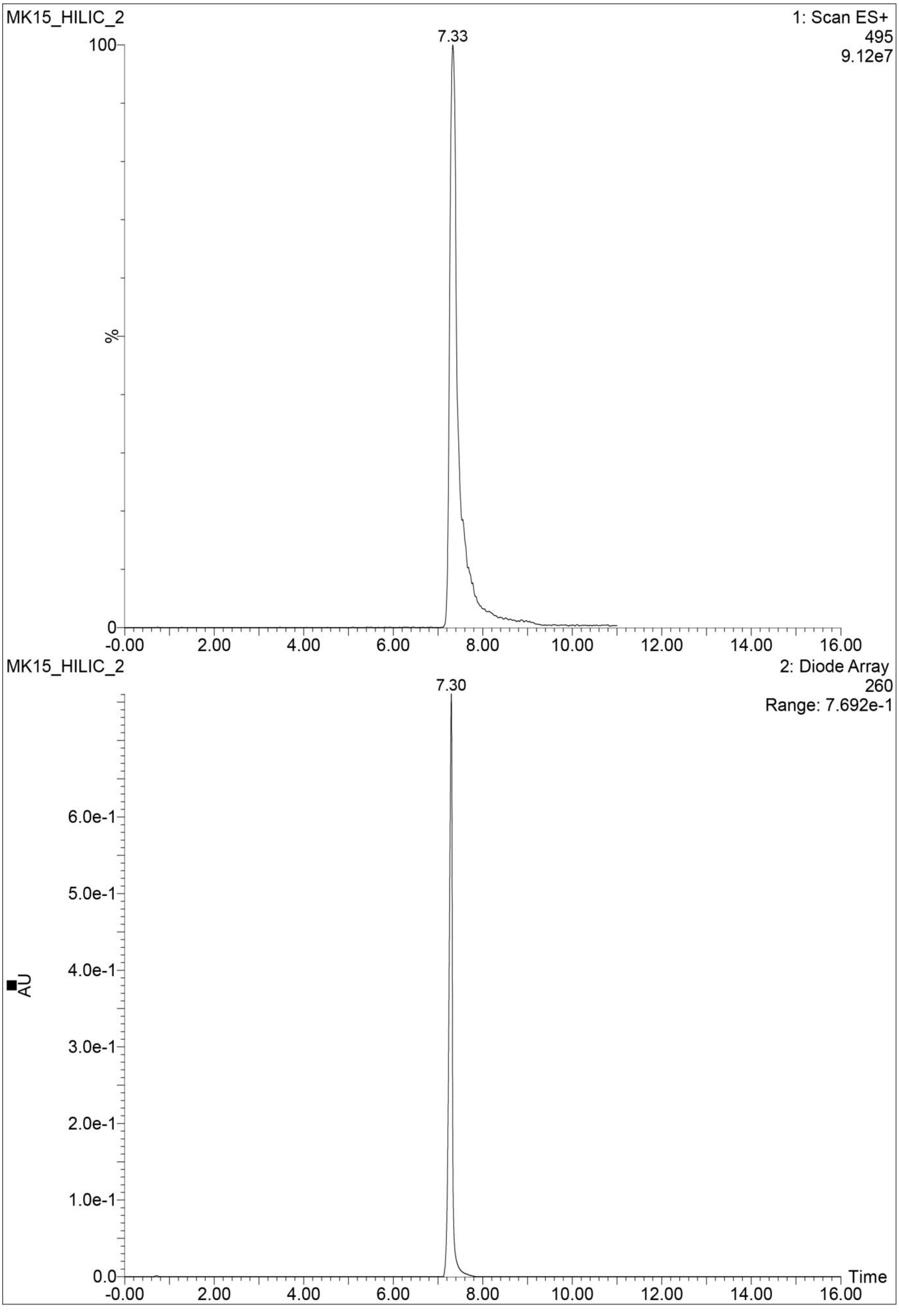

Bis-(L-phenylalanine ethyl ester) prodrug of [2*S*,4*R*] 4-Guanin-9-yl-2-hydroxymethyl-1-*N*-(3-phosphonopropionyl)pyrrolidine 14 Purity 97%

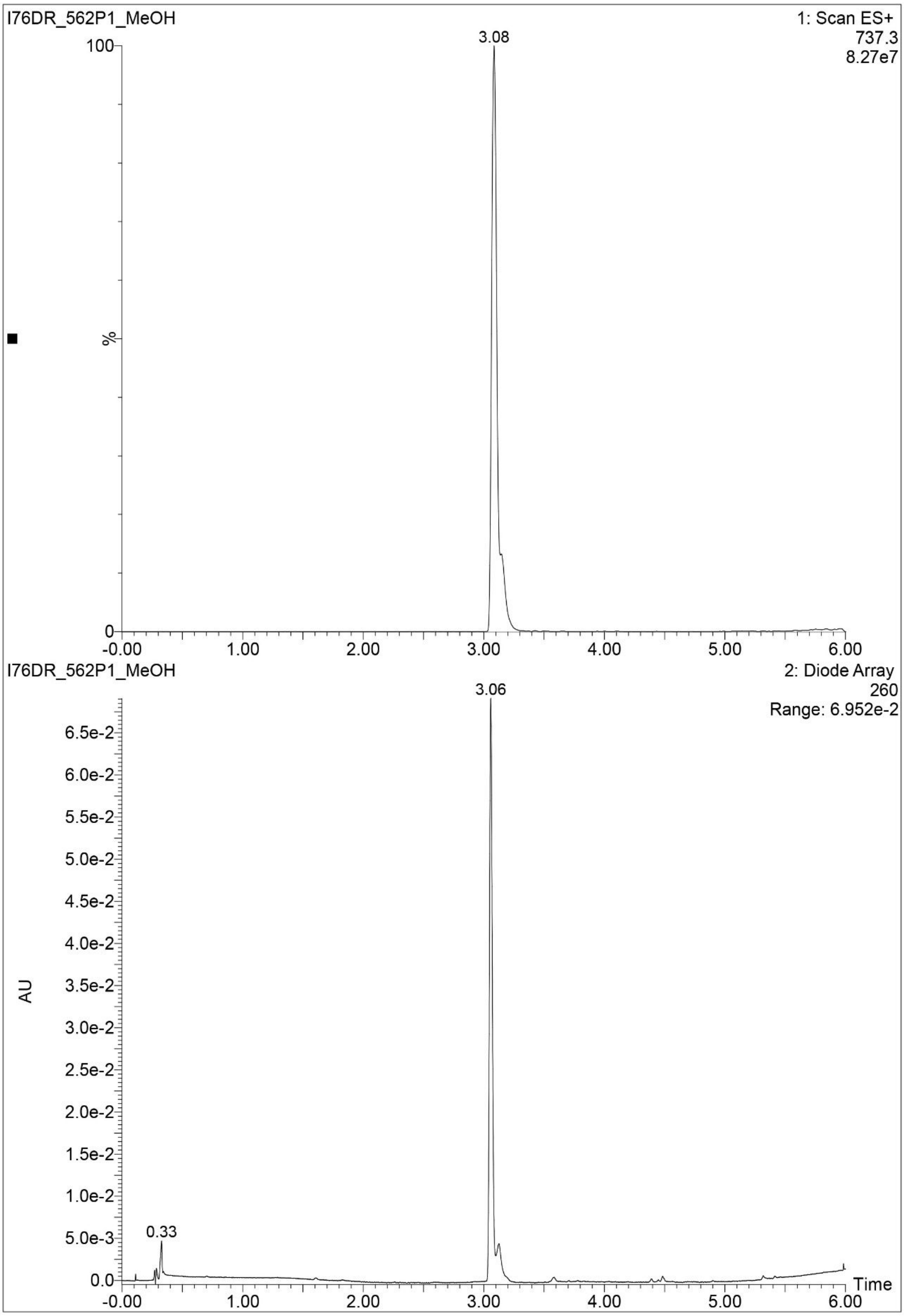

Tetra-(L-phenylalanine ethyl ester) prodrug of [2*S*,4*R*]-4-Guanin-9-yl-2-(2-phosphonoethoxymethyl)-1-*N*-(3-phosphonopropionyl)pyrrolidine 15 Purity 95%

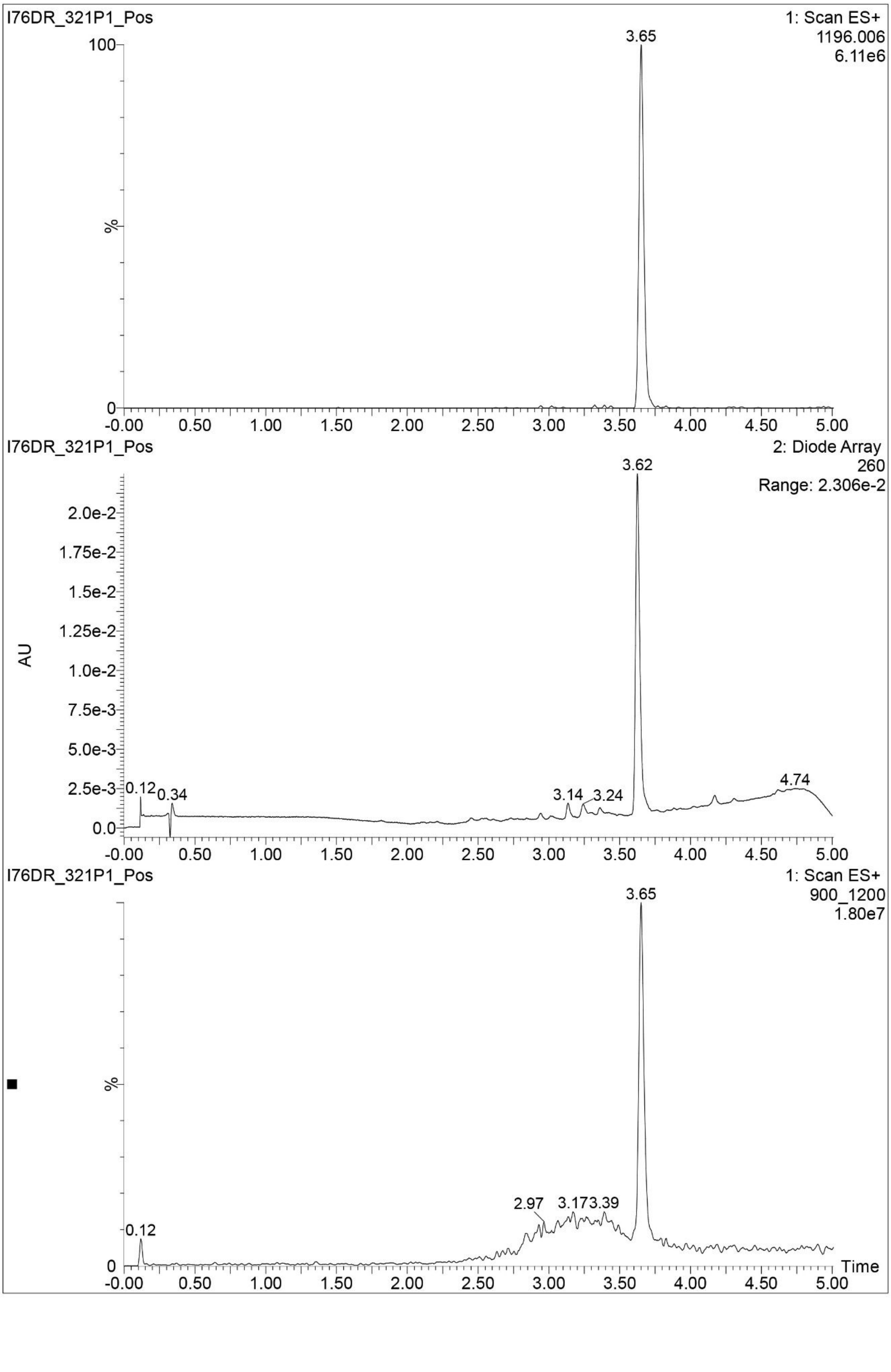

Supplement: Supplementary file 1 — jm4c00021_si_001.pdf [file jm4c00021_si_001.pdf]
